# Control of the assembly of a cyclic hetero[4]pseudorotaxane from a self-complementary [2]rotaxane†

**DOI:** 10.1039/d3sc00886j

**Published:** 2023-03-22

**Authors:** Adrian Saura-Sanmartin, Tomas Nicolas-Garcia, Aurelia Pastor, David Quiñonero, Mateo Alajarin, Alberto Martinez-Cuezva, Jose Berna

**Affiliations:** a Department of Organic Chemistry, Faculty of Chemistry, University of Murcia, Regional Campus of International Excellence “Campus Mare Nostrum” 30100 Murcia Spain ppberna@um.es; b Departamento de Química, Universidad de las Islas Baleares Crta de Valldemossa km 7.5 E-07122 Palma de Mallorca (Baleares) Spain

## Abstract

The synthesis of a ditopic interlocked building-block and its self-assembly into a cyclic dimer is reported herein. Starting from a thread with two recognition sites, a three-component clipping reaction was carried out to construct a bistable [2]rotaxane. A subsequent Suzuki cross-coupling reaction allowed the connection of a second ring to that of the rotaxane, affording a self-complementary ditopic system. NMR studies were carried out to identify a cyclic hetero[4]pseudorotaxane as the main supramolecular structure in solution. Its assembly is the result of a positive cooperativity operating in the hydrogen-bonding-driven assembly of this mechanically interlocked supramolecule, as revealed by computational studies. The increase of the polarity of the solvent allows the disruption of the intercomponent interactions and the disassembly of the hetero[4]pseudorotaxane into the two interlocked units. The disassembly of the cyclic dimer was also achieved through a Diels–Alder reaction over the fumaramide binding site of the thread, triggering the translational motion of the entwined macrocycle to an adjacent glycylglycine-based station and precluding the supramolecular dimerization. The competitive molecular recognition of a guest molecule by one of the self-templating counterparts of the dimer also led to the controlled disassembly of the hetero[4]pseudorotaxane.

## Abstract 2

The self-association of a ditopic [2]rotaxane with two macrocycles mainly leads to a [4]pseudorotaxane which can be reversibly disassembled by adding competitive binders, varying the solvent polarity and changing a binding site affinity.

## Graphical abstract

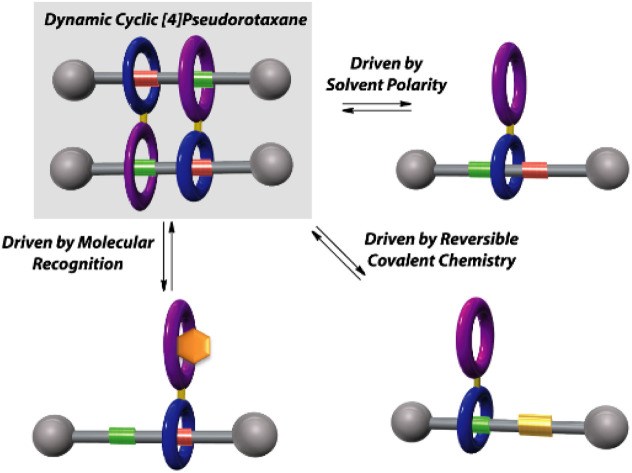

## 1. Introduction

The design of artificial molecular machines that mimic the bio-nanomachines controlling key biological processes attracts nowadays the attention of many research groups.^1,2^ Molecular architectures incorporating two or more components connected through a mechanical bond (mechanically interlocked molecules, MIMs) are considered ideal candidates for this goal.^3^ Stimuli responsive MIMs, whose dynamic properties can be controlled at will, are being incorporated into advanced materials.^4^ The precise arrangement of these components in the material matrix allows the amplification of the intramolecular motions at the macroscopic level.^5^ Hence, the efficient synthesis of MIMs with advanced structural complexity is still highly appreciated.^6^

In this line, compounds able to self-assemble and form supramolecular species or polymers through the formation of mechanical bonds are considered a breakthrough.^7^ Supramolecular chemistry provides powerful tools for the precise construction of these inspiring architectures, assembled through the establishment of intermolecular non-covalent interactions.^8^ In the most useful examples of these supramolecular architectures, changes in some key parameters (solvent polarity, concentration, temperature, …) allow to control its level of organization, as monomers ([1]rotaxanes)^9^ dimers (lineal or [*c*2]daisy chains),^10^ trimers^11^ or oligomers with more complex skeletons.^12^ The formation of [*c*2]daisy chains based on (pseudo)rotaxanes is the most abundant example of this type found in the bibliography and still considered as a prototype for the synthesis of artificial muscles^13^ and molecular actuators in polymeric materials.^14^ The most common strategies for the assembly of these systems lay in various non-covalent interactions such as (a) crown ether-based macrocycles and ammonium ions;^15^ (b) metal–ligand complexation;^16^ (c) cyclodextrin encapsulation;^17^ (d) anion template;^18^ and (e) π-donor/π-acceptor templation motifs,^19^ among others. In contrast, the number of examples reported to date based on the ability of polyamide-based macrocycles to interact by hydrogen-bonding with amide-containing templates (such as peptides) for the formation of such architectures is scarce.^20^

Higher structural complexities can be reached by the use of heterorotaxanes.^21^ These species contain more than one type of entwined macrocycles, a fact that makes them exceptional candidates for investigations on sequence isomerism and stereochemical complexity. Based on such architectures, Qu *et al.* developed the preparation of a hetero[4]rotaxane containing a [*c*2]daisy chain *via* an elegant self-sorting strategy.^22^

**Fig. 1:**
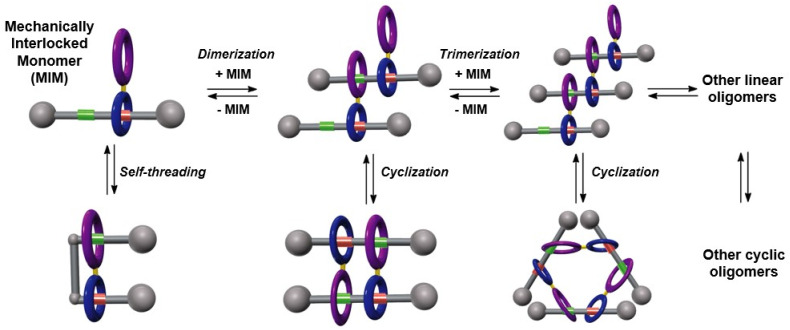
Evolution of an interlocked ditopic monomer towards their potential supramolecular self-assembled structures: molecular lasso, [*cn*]daisy chains, lineal dimers or trimers and oligomers.

Herein we report the design and synthesis of a ditopic amide-based [2]rotaxane, a mechanized monomer that might be involved in a plethora of chemical equilibria between different mechanically-interlocked supramolecular species in solution, as represented in Fig. 1. The main particularity of this system is the presence of a diamide macrocycle linked to the habitual Leigh-type tetraamide ring by means of an aryl–aryl bond, and with a thread bearing two binding sites. NMR experiments and MS data were decisive for unambiguously determining the main supramolecular structure formed in solution, a dimeric cyclic hetero[4]pseudorotaxane with a [*c*2]daisy chain substructure. Computational studies corroborated the formation of this cyclic pseudorotaxane by a cooperative self-assembly. Three different strategies for controlling the disassembly of this mechanically interlocked supramolecular species have been successfully achieved.

## 2. Results and discussion

### 2.1 Synthesis of the self-complementary [2]rotaxane

**Scheme 1:**
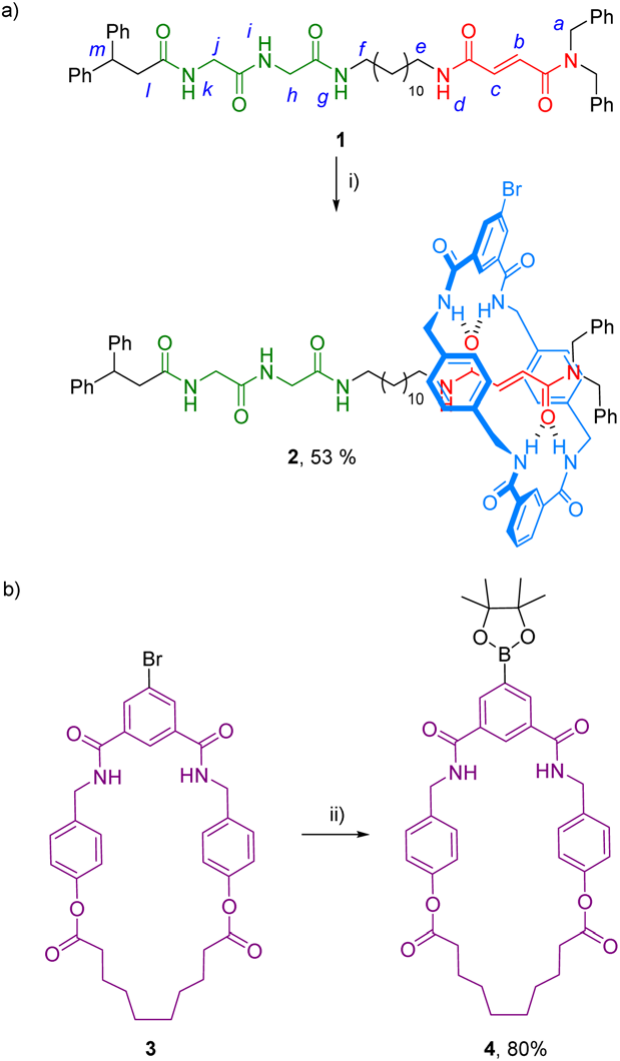
Synthesis of: (a) [2]rotaxane 2; and (b) macrocycle 4. Reaction conditions: (i) *N*^1^,*N*^3^-bis[4-(aminomethyl)benzyl]isophthalamide, 5-bromoisophthaloyl dichloride, Et_3_N, CHCl_3_, 25 °C, 4 h; (ii) bis(pinacolato)diboron, Pd(dppf)Cl_2_ (5 mol%), AcOK, dioxane, 90 °C, 24 h.

The synthesis of the target ditopic rotaxane was accomplished starting from thread 1, bearing two binding stations, a fumaramide^23^ and a glycylglycine (GlyGly) moieties,^24^ tethered by a lineal C-12 alkyl chain (see ESI† for complete synthetic procedures). The three-component clipping reaction between the thread 1, *N*^1^,*N*^3^-bis[4-(aminomethyl)benzyl]isophthalamide^25^ and 5-bromoisophthaloyl chloride in the presence of Et_3_N afforded the interlocked compound 2 in good yield (53%) accompanied by a small amount of the corresponding [3]rotaxane (4%) (Scheme 1a). In parallel the bromo-derived macrocyclic diamide 3 was transformed into the corresponding boronic acid pinacol ester-derived macrocycle 4 (Scheme 1b, see ESI† for further details). Diamide macrocycles similar to 4 have been shown to interact with GlyGly-based threads functionalized with diphenylmethyl groups as stoppers, analogous to that present in 1, forming pseudorotaxanes in a dynamic slippage process with high association constants (∼300 M^−1^).^26^ Thus, we first tested the bromo-substituted macrocycle 4 as receptor of thread 1 in a rotaxanation process. Titration experiments carried out in a solution of 4 (2 mM, CDCl_3_, 298 K) revealed a reasonable interaction with the peptide-based binding site of the thread 1 by forming a 1 : 1 complex with a calculated association constant of 418 M^−1^ (see ESI, Fig. S15 and S16†).

**Scheme 2:**
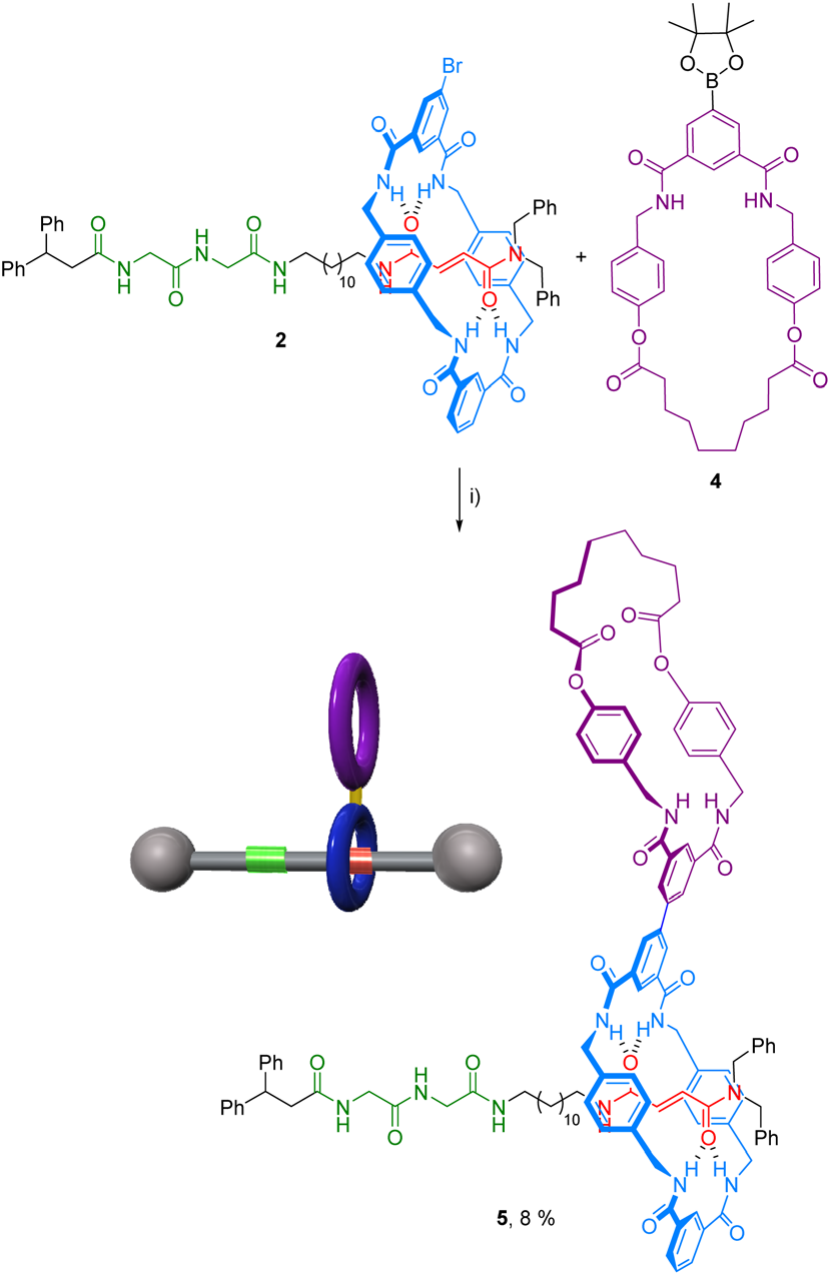
Synthesis of rotaxane 5. Reaction conditions: (i) Pd(dppf)Cl_2_ (20 mol%), K_3_PO_4_, dioxane, 100 °C, 24 h.

With the rotaxane 2 and macrocycle 4 in hand, its Pd-catalyzed cross-coupling reaction towards the formation of the ditopic rotaxane 5 was carried out (Scheme 2). Different reaction conditions were tested, including the variation of base (NaHCO_3_, Na_2_CO_3_, K_2_CO_3_), catalyst [Pd(PPh_3_)_4_ and Pd(dppf)Cl_2_ at different percentages], solvent (dimethylformamide, tetrahydrofuran), temperature or time. Although in all cases low yields of the desired system 5 were obtained, we could isolate pure heterorotaxane 5 in an 8% yield under the best reaction conditions [Pd(dppf)Cl_2_ (5 mol%), AcOK, dioxane, 90 °C, 24 h].

### 2.2 Studies on the self-assembly of rotaxane 5

**Fig. 2:**
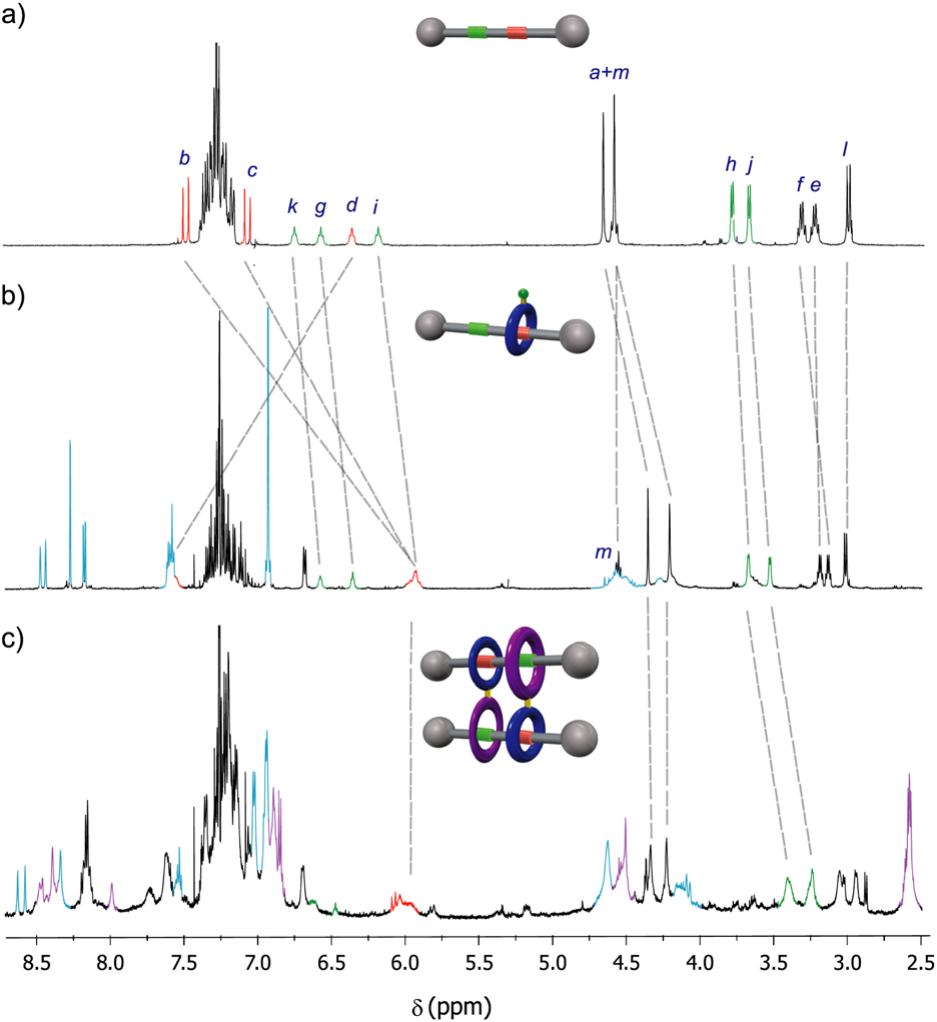
^1^H NMR spectra (400 MHz, CDCl_3_, 298 K) of: (a) thread 1; (b) [2]rotaxane 2; (c) hetero[4]pseudorotaxane 5·5. See lettering in Scheme 1. Signals related to the fumaramide station are highlighted in red. Signals related to the GlyGly station are highlighted in green. Signals related to the diamide macrocycle are highlighted in purple. Signals related to the tetraamide macrocycle are highlighted in light blue. See lettering in Scheme 1.

The ring location over the thread in rotaxanes 2 and 5 was scrutinized by means of ^1^H NMR experiments (Fig. 2). An initial comparison of the ^1^H NMR spectra of the thread 1 with rotaxane 2, recorded in CDCl_3_ (400 MHz, 298 K), allowed us to unequivocally determine the main position of the macrocycle along the thread in the interlocked system (Fig. 2a and b). Whereas the chemical shift of the signals ascribed to the fumaramide binding site (H_b_ and H_c_, red) are importantly upshifted to lower values (Δ*δ*_average_ = 1.32 ppm),^27^ the corresponding signals of the GlyGly station (H_h_ and H_j_, green) remained nearly unaffected. The signal referred to the NH_d_ of the fumaramide is shifted to higher chemical shift (Δ*δ* = 1.18 ppm), as result of the hydrogen-bonding interaction with the polyamide macrocycle (light blue). This scenario clearly points out that the macrocycle in rotaxane 2 is predominantly located over the fumaramide binding site, with the GlyGly station practically unoccupied. Interestingly, the ^1^H NMR spectrum recorded in CDCl_3_ of compound 5, with the second macrocycle attached to the first one, was very complex (Fig. 2c).

After a careful inspection we could assign the most significant signals. Apart from the new peaks corresponding to the novel diamide macrocycle (in purple) and the former tetraamide one (in light blue), the most relevant changes in the spectrum of 5 in CDCl_3_, when compared with that of rotaxane 2, is the displacement to lower chemical shift of the signals H_h_ and H_j_ (in green) part of the GlyGly binding site. This fact indicates that the GlyGly station is now placed inside the diamide macrocycle (in purple). The chemical shift of the signals of the fumaramide (H_b+c_) are not altered when the second macrocycle is attached, thus supporting that compound 5 is probably forming the supramolecular interlocked dimer, the hetero[4]rotaxane 5·5, *via* the self-slippage of the GlyGly station through the second diamide ring (see Fig. 1 for additional putative self-assembly routes of the mechanized monomer 5).

**Fig. 3:**
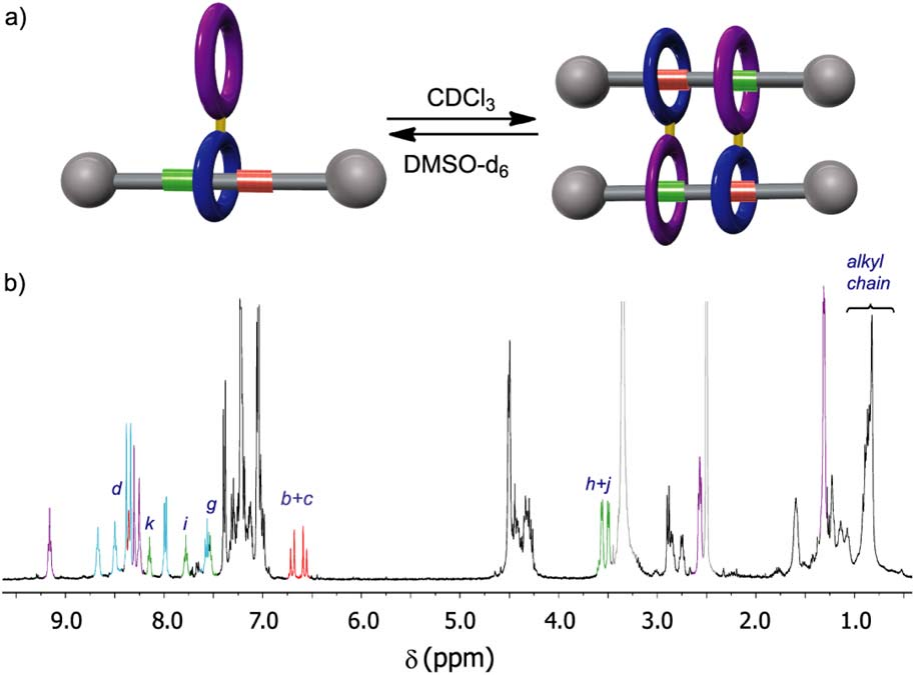
Solvent-dependent assembly–disassembly of the ditopic rotaxane 5: (a) schematic representations of the assembly and disassembly of 5; (b) ^1^H NMR spectra of 5 in DMSO-*d*_6_ (400 MHz, 298 K). See lettering in Scheme 1. Signals related to the fumaramide station are coloured in red. Signals related to the GlyGly station are coloured in green. Signals related to the diamide macrocycle are coloured in purple. Signals related to the tetraamide macrocycle are coloured in light blue. Signals of residual water and the DMSO-*d*_6_ coloured in light grey. See lettering in Scheme 1.

**Fig. 4:**
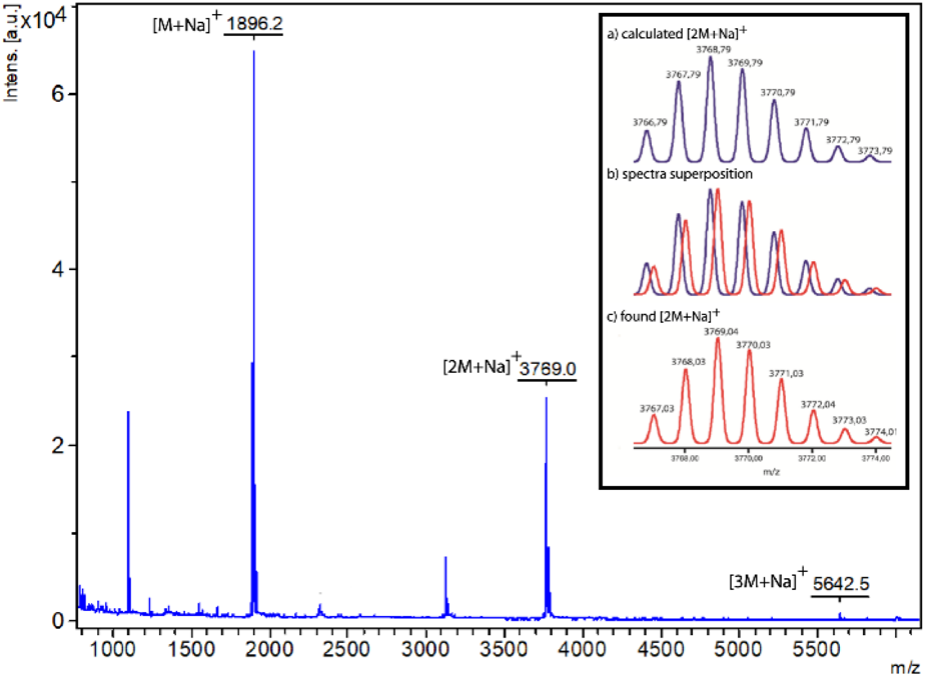
Mass spectra (MALDI-TOF) of ditopic rotaxane 5. Inset: (a) calculated; (b) superposition of calculated and experimental; (c) experimental of rotaxane 5 in its dimeric form [2M + Na]^+^.

Although the ^1^H-NMR spectrum of 5 in CDCl_3_ is notably complex, in sharp contrast its spectrum recorded in DMSO-*d*_6_ is much more simple, showing fewer and better-defined signals (Fig. 3b). The employment of DMSO, a highly polar solvent able to strongly compete for the establishment of hydrogen-bonding interactions,^28^ apparently precluded the assembly of the supramolecular aggregate 5·5 occurring in CDCl_3_. Thus, the formation or not of the supramolecular dimer can be controlled by selecting media of different polarity, the use of a very polar solvent such as DMSO completely precluding the assembly (Fig. 3a). The multiple possibilities in the self-assembly of the ditopic compound 5*via* threading of the GlyGly moiety through the diamide macrocycle made difficult the analysis of the ^1^H NMR spectra of 5 in CDCl_3_ solution. Consequently, we could not unambiguously distinguish between the presence in that solution of a monomer (linear or self-threaded), a dimer (cyclic or acyclic), trimeric species or larger assembled structures. Therefore, a MALDI-TOF experiment was performed in order to observe the formation of one or several of those stable supramolecular aggregates. Indeed, the obtained spectra showed up that compound 5 forms dimeric species in a significant amount, whilst identifying also the monomer. The experimental isotopic distribution of the peaks corresponding to the molecular ion of the 5·5 dimer was in total agreement with the calculated ones (Fig. 4, inset). The MALDI-TOF experiment also shows the formation of traces of trimeric supramolecular structures ([3M + Na]^+^ 5642.5).

### 2.3 NMR diffusion measurements of rotaxane 5

To estimate the molecular volume of the main molecular assembly of 5 in CDCl_3_ we measured its diffusion coefficient (*D*) by NMR experiments (Table 1, see ESI for experimental details, Fig. S1†).^29^

**Table 1:** Diffusion coefficients, *D* (m^2^ s^−1^) and hydrodynamic radii, *r*_H_ (Å) for 2 mM solutions at 298 K. Rotational radii, *r*_Rot_ (Å) calculated from molecular models and hypothetical diffusion coefficient *D*_prolate_ (m^2^ s^−1^) considering that the molecule has an ellipsoid shape

Ent.	Comp. (solv.)	*D* a/10^−10^	*r*_H_ b	*r*_Rot_	*D*_prolate_/10^−10^
1	3 (CDCl_3_)	7.33^c^	5.6	8.4	7.72
2	1 (CDCl_3_)	5.76^d^	7.1	7.6	—
3	2 (CDCl_3_)	4.76^e^	8.6	8.8	—
4	5 (CDCl_3_)	3.68^f^^,^^g^	11.1	17.8 (5·5)	3.30 (5·5)
				10.9 (5)	5.08 (5)
5	5 (DMSO-*d*_6_)	0.98^h^	11.2	—	—

a Experimental error: ±2.0%.

b Standard deviation: ±0.1 Å.

c At 8.12 and 2.57 ppm.

d At 4.64 and 3.77 ppm.

e At 8.26 and 4.36 ppm.

f At 2.58 ppm.

g The diffusion coefficients at 8.63, 8.16, 8.00 and 3.26 ppm are in the range of 3.65–4.14 × 10^−10^ m^2^ s^−1^ (*r*_H_ in the range of 9.8–11.2 Å).

h Measured at 7.99 and 1.59 ppm. *η* (CHCl_3_, 298 K) = 0.536 × 10^−3^ kg s^−1^ m^−1^; *η* (DMSO, 298 K) = 1.991 × 10^−3^ kg s^−1^ m^−1^.^37^

For comparison, we also measured the *D* values of macrocycle 3, thread 1 and [2]rotaxane 2 under the same conditions (Table 1, entries 1–3). As expected, the supramolecular assembly 5 showed the lowest diffusion coefficient (*D* = 3.68 × 10^−10^ m^2^ s^−1^, Table 1, entry 4). Diffusion coefficients and molecular radii are correlated by the Stokes–Einstein equation.^30^ It has been postulated^31^ that the volume of a molecular species, considering a relatively spherical shape, is proportional to its molecular weight. Thus, the equation can be linearized by taking the logarithm of both magnitudes.^32^ We established a calibration curve with *D* values of amide-based rotaxanes previously described in the literature^20^ and that of [2]rotaxane 2, showing a good correlation (see Fig. S2, S3 and Table S1†). The lack of a perfect coincidence can be attributed to changes in the global shape or solvent interactions.^33^ With this formula in our hands and the experimental diffusion coefficient value of the supramolecular assembly 5 in CDCl_3_ we obtained an approximate molecular weight of 2708.4 g mol^−1^, which turned out to be halfway between those of the monomer 5 and the dimer 5·5.

The hydrodynamic radii (*r*_H_) of macrocycle 3, thread 1, [2]rotaxane 2 and the supramolecular assembly 5 were calculated from their respective *D* values by using the Stokes–Einstein equation (Table 1).^30^ Next, the computed structures^34^ of 1–3, 5 and the hetero[4]rotaxane 5·5 were introduced into the Spartan'16 software and their rotational radii (*r*_Rot_)^35^ were estimated as being half of the longest distance within the relatively spherical molecules (see ESI, Fig. S4–S8†). The respective *r*_Rot_ values of thread 1 (7.6 Å) and [2]rotaxane 2 (8.8 Å) are in fairly good agreement with their hydrodynamic radii (7.1 Å and 8.6 Å, respectively) obtained from the *D* values (Table 1, entries 2–3). However, those corresponding to macrocycle 3 (8.4 Å) and [4]rotaxane 5·5 (17.8 Å) are far from being realistic due to the elliptical shapes shown by their computed structures. For that reason, we calculated the theoretical *D* values of macrocycle 3 (7.72 × 10^−10^ m^2^ s^−1^) and [4]rotaxane 5·5 (3.30 × 10^−10^ m^2^ s^−1^) considering these structures as prolates, which fit well with those experimentally determined (Table 1, entries 1 and 4).^36^ By contrast, the hypothetical diffusion coefficient of monomeric 5 when considering an elliptical shape (5.08 × 10^−10^ m^2^ s^−1^) is quite far from that obtained from the NMR experiments (Table 1, entry 4). Thus, we can conclude that the experimental diffusion data are consistent with the presence of a compact and elliptical shaped [4]rotaxane 5·5 as the major species in CDCl_3_.

To further investigate a potential equilibrium between 5 and 5·5, we also determined the diffusion coefficient in CDCl_3_ over the range of concentrations 2–10 mM (see ESI, Table S2†). The *D*-value of the supramolecular assembly 5 increases up to 10% on going from 10 mM (3.38 × 10^−10^ m^2^ s^−1^) to 2 mM (3.71 × 10^−10^ m^2^ s^−1^). However, we observed an analogous growth for the *D*-values of rotaxane 2, unable to self-assemble, at the same concentrations. These results seem to indicate that the assembly coming from 5 does not experience any change in this range of concentrations.

Finally, we measured the diffusion coefficient of 5 in DMSO-*d*_6_ where it exists as the monomer (Table 1, entry 5). In this solvent of higher viscosity, the *D* value of 5 is consistently smaller. However, its *r*_H_ calculated from the Stokes–Einstein equation turned out to be very similar to that determined in CDCl_3_. At first sight, the similar hydrodynamic radii of 5 in CDCl_3_ and DMSO-*d*_6_, seems to contradict our previous assumptions. However, it should be taken into account that the diffusion speed is very sensitive to molecular shape and solvation effects, that can be very different in both solvents.^38^ Thus, the obtained results are consistent with the presence of a compact elliptical dimer 5·5 as the major species in CDCl_3_ and a less compact and highly solvated monomer 5 in DMSO-*d*_6_.

### 2.4 Computational studies for the self-assembly of rotaxane 5

**Fig. 5:**
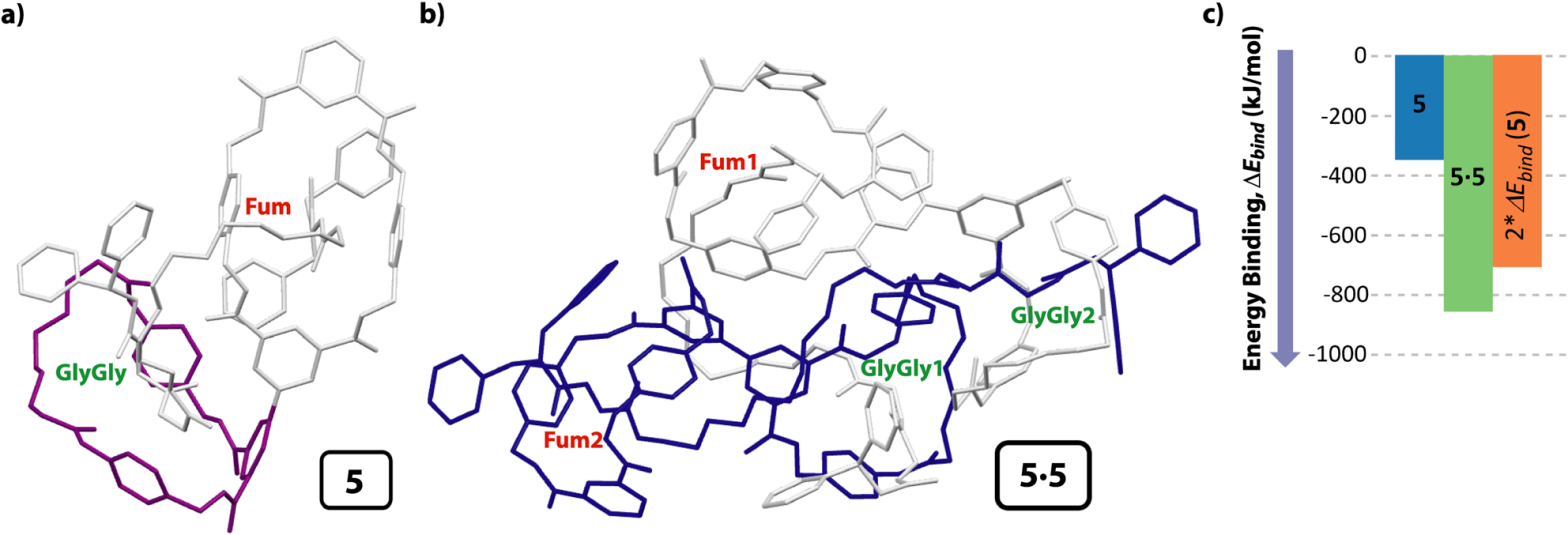
Computational models at DFT level in CHCl_3_ (BP86-D3/def2-SVP) of: (a) [2]rotaxane 5; (b) cyclic hetero[4]pseudorotaxane (dimer 5·5); (c) diagram for visually comparing the binding energies of the monomer 5, the cooperatively self-assembled dimer 5·5 and that of the double of binding energy of the monomer 5. Both calculated structures are the lowest energy ones; Δ*E*_bind_: variation of the binding energy calculated as the difference between the energy of the complex minus the energy of all separated molecules that formed it (of the conformer with the lowest energy). For clarity the hydrogen atoms have been removed. Note that in the cyclic dimer 5·5, one of the molecules is colored in grey and the second one in dark blue. For clarity the diamide macrocycle is colored in purple in monomer 5.

In order to find further evidences concerning the formation of the cyclic [4]pseudorotaxane in solution, we also carried out computational studies. After a deep conformational study of the individual components present in system 5 (thread 1 and the unthreaded tetraamide and diamide macrocycles) by molecular mechanics (MMFF94), we calculated the potential intermolecular and intramolecular interactions that can be established when the supramolecular aggregates are formed, both in the gas phase and in solution (CHCl_3_), optimizing their geometries at a DFT level (BP86-D3/def2-SVP). After analyzing a range of the possible assemblies of 5 (*i.e.* molecular lasso, lineal dimers, *etc.*), we found that the formation of the [*c*2]daisy chain was the energetically most favorable structure. Moreover, the computations show that the encapsulation of the two GlyGly stations inside the cavity of the two diamide macrocycles (one of each molecule) to form the cyclic dimer 5·5 is neatly favorable *via* a cooperativity phenomenon. Thus, the difference of the binding energy between the components to form the monomer of 5 was only −353.5 kJ mol^−1^ (Fig. 5a), whereas the binding energy of the cyclic dimer 5·5, having the two GlyGly stations inside the cavity of the diamide rings, was considerably higher (−864.1 kJ mol^−1^) (Fig. 5b). Note that the GlyGly station in the monomer 5 is interacting with the diamide macrocycle (highlighted in purple) but not inside its cavity, thus not forming a molecular lasso (Fig. 5a). As the binding energy of 5·5 is lower than twice that of the monomer 5 we assume that some kind of cooperativity should be operating in the formation of the doubly interlocked dimer 5·5 (Fig. 5c). Thus, these computations show that the formation of the cyclic hetero[4]pseudorotaxane is favored over other supramolecular architectures of lower level of complexity. Moreover, the computed structure of 5·5 (Fig. 5b) is in accord with that deduced from the NMR experiments (Fig. 2), with the tetraamide macrocycle (in blue) located over the fumaramide binding site (in red) and the diamide ring (in purple) over the GlyGly function (in green) (see colours in Scheme 2).

### 2.5 Studies on the stimuli-responsive disassembly of the [4]pseudorotaxane

**Fig. 6:**
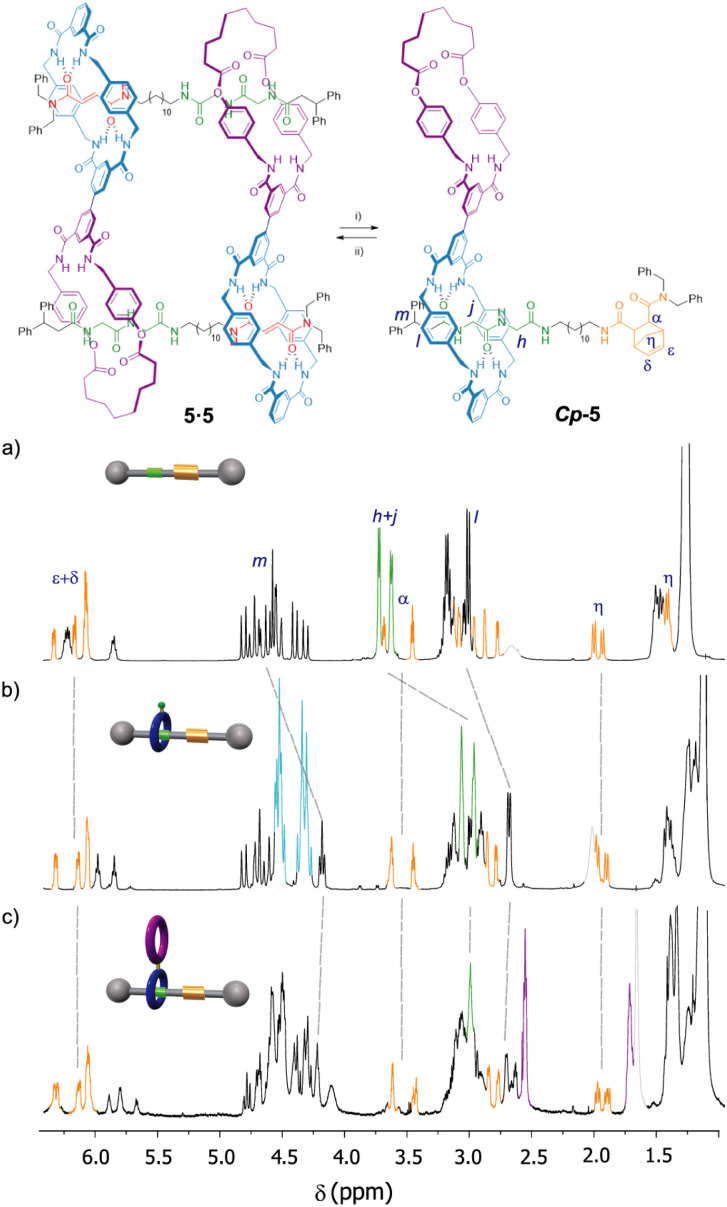
Controlled disassembly and assembly of the dimer 5·5 by a reversible chemical modification. Partial spectra (600 MHz, CDCl_3_, 298 K) of: (a) thread *Cp*-1; (b) bromo-derived rotaxane *Cp*-2; (c) rotaxane *Cp*-5. Reaction conditions: (i) cyclopentadiene, DMSO, 80 °C, 16 h, 48%; (ii) 235 °C, 0.5 mm Hg, 30 min, ∼41%. Signals related to the Diels–Alder adduct are coloured in orange (for clarity, only one diastereoisomer is drawn). Signals related to the GlyGly station are coloured in green. Signals related to the diamide macrocycle are coloured in purple. Signals related to the tetraamide macrocycle are coloured in light blue.

At this point we wondered how to inhibit the self-assembly of the monomer 5. We envisaged that the formation of the cyclic dimer 5·5 could be dampened by converting the fumaramide binding site of 5 into another functionality with negligible affinity for the tetraamide macrocycle, forcing in this way the translation of this ring to the nearby GlyGly station and thus precluding its dimerization. The control of the translational motion in fumaramide-containing rotaxanes have been extensively investigated due to the versatile switchability of this function by means of isomerization by light irradiation^39^ or chemical transformations. The Diels–Alder reaction of a diene (typically, cyclopentadiene) with the fumaramide function at the thread of a bistable rotaxane has been reported to efficiently promote the shuttling of its entwined polyamide macrocycle towards an adjacent station.^40^ With this precedent in mind, we carried out the Diels–Alder reaction between 5 and cyclopentadiene affording the expected adduct *Cp*-5 in 48% yield^41^ as a 1 : 1 mixture of two diastereoisomers (Fig. 6). A deep analysis of the NMR spectra of *Cp*-5, by comparing with the spectra of similar Diels–Alder adducts of the thread *Cp*-1 and the bromo-derived rotaxane *Cp*-2 (see ESI† for synthetic details), allowed us to assess that *Cp*-5 is not a cyclic dimer (Fig. 6). The signals of the new station (bicycloheptene Diels–Alder product, in orange) appears at similar chemical shifts in the three adducts indicating that, in the case of the rotaxanes *Cp*-5 and *Cp*-2, there is not interaction of the new stations with the tetraamide ring. In addition, the signals due to the GlyGly function (green colour in Fig. 6) are shifted to higher field when comparing to those of the thread *Cp*-1 and the rotaxanes *Cp*-2 and *Cp*-5, indicating that the respective tetraamide macrocycle is in fact interacting with the GlyGly station and, as a consequence, precluding the self-assembly of the doubly interlocked structure. Moreover, the position of the macrocycle at this side of the thread is also evidenced by a drastic shielding of the signals related to the adjacent stopper (H_l_ and H_m_). Remarkably, the retro-Diels–Alder reaction of *Cp*-5 under thermal and high vacuum conditions, resulted in the re-assembly of the cyclic dimer 5·5 (see ESI† for further synthetic details).

**Fig. 7:**
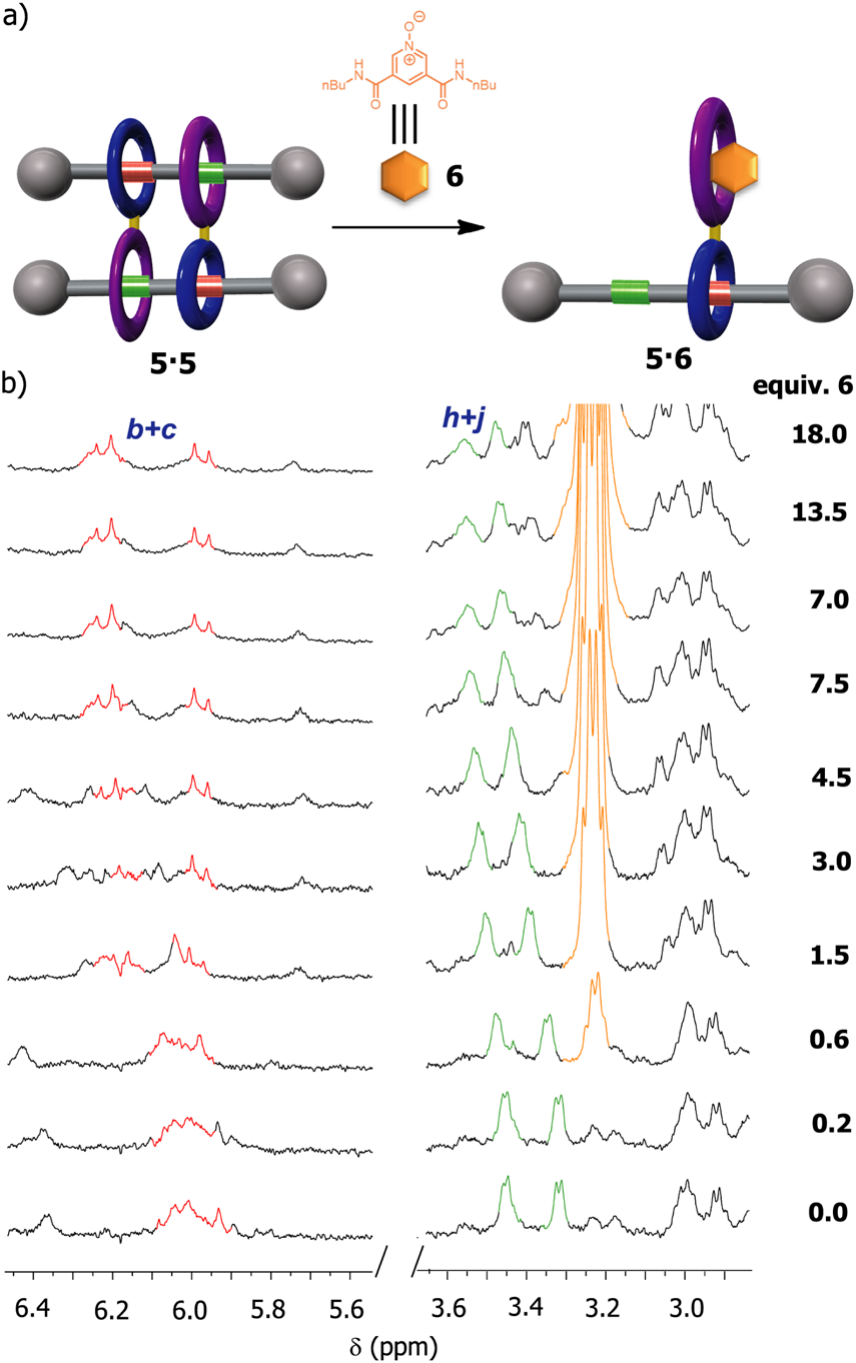
Disassembly of the dimer 5·5 by competitive recognition events: (a) schematic representations of the disassembly of 5·5 in the presence of the *N*-oxide derivative 6; (b) partial ^1^H NMR spectra (400 MHz, CDCl_3_, 298 K) of 5 during the addition of an increasing amount of the *N*-oxide 6. See lettering in Scheme 1. Signals related to the fumaramide station are coloured in red. Signals related to the GlyGly station are coloured in green. Signals related to the *N*-oxide 6 are coloured in orange.

We were also intrigued by the possibility of using an external guest as a competitor for the occupation of the diamide macrocycle present in 5, precluding the formation of the supramolecular dimer. Pyridine *N*-oxide derivatives have been proven to interact with diamide macrocycles, analogous to that in 5, by forming stable pseudorotaxanes.^42^ Thus, we expected that the addition of the pyridine *N*-oxide derivative 6, with butyl groups at the ends (see ESI for further details, Fig. S17 and S18†), to a solution of 5 in CDCl_3_ could prevent the formation of the cyclic [4]rotaxane (Fig. 7). First, we performed titration experiments by adding pyridine *N*-oxide 6 to a solution of the bromo-substituted macrocycle 3 (1 mM, CDCl_3_, 298 K), calculating an association constant of 518 M^−1^ for the 1 : 1 complex (see ESI† for further details), a higher value than that measured for the complex thread 1: macrocycle 3 (418 M^−1^). Next, we proved that the addition of up to 18 equiv. of 6 to a solution of 5 (2 mM, CDCl_3_, 298 K) was necessary for avoiding the formation of the supramolecular dimer as result of the putative assembly of the new pseudorotaxane 5·6. Having in mind that the association constant between the macrocycle 3 and the *N*-oxide 6 was same order of that of 3 with thread 1 (518 M^−1^*vs.* 418 M^−1^), the disassembly of the dimer 5·5 required an excess of *N*-oxide 6 for totally disrupting the formation of the supramolecular dimer. In this regard, it must be also considered that this process is even made more difficult by the cooperativity phenomenon behind the assembly of 5·5.

## 3. Conclusions

The synthesis of a ditopic amide-based mechanically interlocked system has been satisfactorily addressed. The capacity of this system for self-assembly has been deeply studied by experimental and computational techniques. Solutions of the complementary rotaxane in a noncompetitive solvent allows the hydrogen-bonding-driven assembly of a novel cyclic hetero[4]pseudorotaxane, with a [*c*2]daisy chain substructure. The survival of this supramolecular architecture has been proven by NMR and MS analysis, and also by computational methods, supporting the positive cooperativity phenomenon that assists its assembly. Remarkably, the controlled disassembly of the supramolecular architecture can be achieved by different methods, such as increasing the polarity of the solvent, by addition of a competitive organic molecule and by performing a reversible chemical transformation that induces an internal translational movement of the ring along the thread. All these methods reduce the level of association of the systems up to the monomer. The synthetic methodology shown here might be used in the preparation of other mechanically interlocked systems displaying intricated structures and enabled for switching functions.

## Data availability

Experimental procedures, characterization data, titration experiments, computational data and Cartesian coordinates are available in the ESI.†

## Author contributions

The manuscript was written through contributions of all authors. All authors have given approval to the final version of the manuscript.

## Conflicts of interest

There are no conflicts to declare.

## Supplementary Material

SC-014-D3SC00886J-s001
