# Supplementary material for: Control of the assembly of a cyclic hetero[4]pseudorotaxane from a self-complementary [2]rotaxane
Source: Chem Sci. 2023 Mar 22;14(15):4143–51. doi: 10.1039/d3sc00886j (PMC10094293; doi:10.1039/d3sc00886j)
Supplement: SC-014-D3SC00886J-s001 [file SC-014-D3SC00886J-s001.pdf]

Electronic Supplementary Material (ESI) for Chemical Science.
This journal is © The Royal Society of Chemistry 2023

Control of the assembly of a cyclic hetero[4]pseudorotaxane from a self-complementary [2]rotaxane

Adrian Saura-Sanmartin,[a] Tomás Nicolas-Garcia,[a] Aurelia Pastor,[a] David Quiñonero,[b] Mateo Alajarin,[a] Alberto Martinez-Cuezva,[a] Jose Berna*,[a]

[a] Departamento de Química Orgánica, Facultad de Química, Regional Campus of International Excellence "Campus Mare Nostrum", Universidad de Murcia, E-30100, Murcia, Spain

[b] Departamento de Química, Universidad de las Islas Baleares, Crta de Valldemossa km 7.5, E-07122, Palma de Mallorca (Baleares), Spain.

E-mail: ppberna@um.es

Table of Contents.. S2

1. General experimental section.. S3

2. Synthesis of thread 1 .... S4

3. Synthesis of macrocycle 3 .... S6

4. Synthesis of macrocycle 4 .... S7

5. Synthesis of [2]rotaxane 2 and [3]rotaxane S4 .... S8

6. Synthesis of [2]rotaxane 5 .... S10

7. Controlled disassembly and assembly of 5·5 through DA and retro-DA reactions ... S11

8. Synthesis of pyridine *N*-oxide derivative 6 .... S14

9. DOSY NMR experiments S14

10. MALDI-TOF spectra of compound 5 .... S22

11. Titration experiments S23

12. Disassembly of dimer 5·5 by addition of the pyridine *N*-oxide 6 .... S26

13. ^{1}H and ^{13}C NMR spectra of synthesized compounds S27

14. Computational Studies... S39

15. References S55

1. General Experimental Section

Unless stated otherwise, all reagents were purchased from Aldrich Chemicals and used without further purification. HPLC grade solvents (Scharlab) were nitrogen saturated and were dried and deoxygenated using an Innovative Technology Inc. Pure-Solv 400 Solvent Purification System. Column chromatography was carried out using silica gel (60 Å, 70-200 μm, SDS) as stationary phase, and TLC was performed on precoated silica gel on aluminum cards (0.25 mm thick, with fluorescent indicator 254 nm, Fluka) and observed under UV light. All melting points were determined on a Kofler hot-plate melting point apparatus and are uncorrected. ^{1}H- and ^{13}C-NMR spectra were recorded at 298 K on a Bruker Avance 300, 400 and 600 MHz instruments. ^{1}H-NMR chemical shifts are reported relative to Me_4Si and were referenced via residual proton resonances of the corresponding deuterated solvent whereas ^{13}C NMR spectra are reported relative to Me_4Si using the carbon signals of the deuterated solvent. Signals in the ^{1}H- and ^{13}C-NMR spectra of the synthesized compounds were assigned with the aid of DEPT, or two-dimensional NMR experiments (COSY, NOESY, ROESY, HMQC and HMBC). Abbreviations of coupling patterns are as follows: br, broad; s, singlet; d, doublet; t, triplet; q, quadruplet; m, multiplet. Coupling constants (*J*) are expressed in Hz. High-resolution mass spectra (HRMS) were obtained using a time-of-flight (TOF) instrument equipped with electrospray ionization (ESI). Matrix-Assisted Laser Desorption/Ionization (MALDI) mass spectra were acquired on a Bruker AutoFLEX III TOF/TOF mass spectrometer on reflector mode, using dithranol as matrix. The spectra were calibrated by using poliethylenglycol as standard.

Abbreviation list:
THF: tetrahydrofuran
DMSO: dimethylsulfoxide
DMAP: dimethylaminopyridine
EDCI·HCl: *N*-(3-Dimethylaminopropyl)-*N'*-ethylcarbodiimide hydrochloride
TFA: trifluoroacetic acid
dppf: 1,1'-bis(diphenylphosphino)ferrocene

2. Synthesis of thread 1

Fragment S1

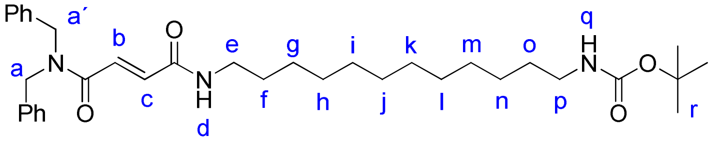

To a suspension of (*E*)-4-(dibenzylamine)-4-oxobut-2-enoic acid[1] (2.95 g, 10.00 mmol), *tert*-butyl (12-aminododecyl)carbamate (3.30 g, 11.00 mmol) and DMAP (1.34 g, 11.00 mmol) in dry dichloromethane (50 mL) at 0 °C was added EDCI·HCl (2.11 g, 11 mmol) under nitrogen atmosphere. The reaction mixture was stirred at room temperature for 36 hours and, after this time, washed with an aqueous solution of 1M HCl (2 x 75 mL), a saturated solution of $NaHCO_3$ (2 x 75 mL) and brine (100 mL). The organic phase was dried over anhydrous $MgSO_4$ and concentrated under reduced pressure. The resulting solid was purified by column chromatography using a mixture hexane/ethyl acetate 6/4 (v/v) as eluent. The product was obtained as a white solid (**S1**, 4.34 g, 7.7 mmol, 77 %); **m. p.** 81 – 83 °C; **^{1}H-NMR** (300 MHz, $CDCl_3$): δ (ppm) 7.47 (d, J = 14.7 Hz, $1H_b$), 7.40 – 7.27 (m, $6H_{Ar}$), 7.23 – 7.10 (m, $5H_{Ar+c}$), 6.38 (t, J = 5.6 Hz, $1H_d$), 4.65 (s, $2H_a$), 4.56 (s, $2H_{a'}$), 4.49 (s, $1H_q$), 3.27 (dd, J = 13.3, 6.8 Hz, $2H_e$), 3.13 – 3.05 (m, $2H_p$), 1.53 – 1.41 (m, $13H_{f+o+r}$), 1.29 – 1.21 (m, $16H_{g-n}$); **^{13}C-NMR** (75 MHz, $CDCl_3$): δ (ppm) 166.2 (C=O), 164.2 (C=O), 156.2 (C=O), 136.7 (C), 136.1 (CH), 136.0 (C), 129.7 (CH), 129.1 (CH), 128.9 (CH), 128.3 (CH), 128.1 (CH), 127.8 (CH), 126.9 (CH), 79.2 (C), 50.3 (CH_2), 48.6 (CH_2), 40.9 (CH_2), 40.0 (CH_2), 30.2 (CH_2), 29.6 (CH_2), 29.6 (CH_2), 29.4 (CH_2), 29.4 (CH_2), 28.6 (CH_3), 27.0 (CH_2), 26.9 (CH_2); **HRMS (ESI)** calcd for $C_{35}H_{52}N_3O_4$ $[M + H]^+$ 578.3952, found 578.3956.

(3,3-diphenylpropanoyl)glycylglycine (S2)

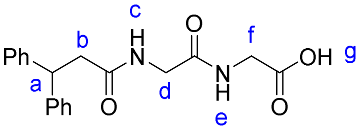

To a solution of glycylglycine (2.56 g, 19.38 mmol) in a 1.5 M NaOH aqueous solution (13 mL) at 0 °C was added dropwise a solution of 3,3-diphenylpropanoyl chloride (2.16 g, 8.83 mmol) in acetone (20 mL) under nitrogen atmosphere, and the mixture stirred for 30 minutes. A solution of 1 M HCl was added until pH 1 was reached. The solvent was concentrated under reduced pressure and the precipitate was filtered under vacuum, washed exhaustively with water, diethyl ether and hexane, and dried. The product was isolated as a white solid (**S2**, 2.70 g, 7.95 mmol, 90 %); **m. p.** 95 – 97 °C; **^{1}H-NMR** (400 MHz, DMSO-d_6): δ (ppm) 12.64 (s, $1H_g$), 8.19 (t, J = 5.7 Hz, $1H_e$), 7.83 (t, J = 5.8 Hz, $1H_c$), 7.30 – 7.20 (m, $8H_{Ar}$), 7.18 – 7.12 (m, $2H_{Ar}$), 4.49 (t, J = 7.9 Hz, $1H_a$), 3.70 (d, J = 5.8 Hz, $2H_d$), 3.63 (d, J = 5.7 Hz, $2H_f$), 2.94 (d, J = 7.9 Hz, $2H_b$); **^{13}C-NMR** (100 MHz, DMSO-d_6): δ (ppm)

171.0 (C=O), 170.5 (C=O), 169.2 (C=O), 144.4 (C), 128.3 (CH), 127.5 (CH), 126.1 (CH), 46.5 (CH), 41.7 (CH_2), 40.9 (CH_2), 40.5 (CH_2); **HRMS (ESI)** calcd for $C_{19}H_{21}N_2O_4$ $[M + H]^+$ 341.1496, found 341.1491.

Thread 1

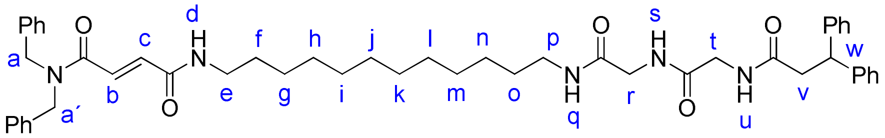

To a solution of fragment **S1** (2.00 g, 3.56 mmol) in chloroform (30 mL) was added TFA (2.70 mL, 35.60 mmol). The reaction mixture was stirred under nitrogen atmosphere for 12 hours. After this time, the solvent was removed under reduced pressure. The resulting residue was dissolved in a mixture of dichloromethane/methanol 1/1 (v/v) (30 mL) and neutralized by using Amberlyst® A21. After 1 hour, the solution was filtered, and the solvent removed under reduced pressure.

To a solution of the resulting residue (1.68 g, 3.52 mmol), carboxylic acid **S2** (1.45 g, 4.27 mmol), Et_3N (1.50 mL, 10.68 mmol) and DMAP (0.58 g, 4.27 mmol) in dry dichloromethane (100 mL) at 0 ºC was added EDCI·HCl (0.82 g, 4.27 mmol) under nitrogen atmosphere. The reaction mixture was stirred at room temperature for 24 hours. After this time the reaction mixture was washed with 1 M HCl (2 x 50 mL), a saturated solution of $NaHCO_3$ (2 x 50 mL) and brine (75 mL). The organic phase was dried over anhydrous $MgSO_4$ and concentrated under reduced pressure. The residue was purified by column chromatography using a mixture chloroform/methanol 95/5 (v/v) as eluent. The product was obtained as a white solid (**1**, 2.25 g, 2.82 mmol, 80%); **m.p.** 58 – 60 ºC; **^{1}H-NMR** (400 MHz, $CDCl_3$): δ (ppm) 7.47 (d, J = 14.7 Hz, $1H_b$), 7.37 – 7.14 (m, $20H_{Ar}$), 7.10 (d, J = 14.7 Hz, $1H_c$), 6.95 (t, J = 5.4 Hz, $1H_u$), 6.78 (t, J = 5.6 Hz, $1H_q$), 6.44 (t, J = 5.7 Hz, $1H_d$), 6.21 (t, J = 6.0 Hz, $1H_s$), 4.64 (s, $2H_a$), 4.60 – 4.54 (m, $3H_{a'+w}$), 3.77 (d, J = 5.4 Hz, $2H_t$), 3.65 (d, J = 6.0 Hz, $2H_r$), 3.33 – 3.26 (m, $2H_e$), 3.24 – 3.27 (m, $2H_p$), 2.99 (d, J = 8.2 Hz, $2H_v$), 1.58 – 1.44 (m, $4H_{f+o}$) 1.28 – 1.24 (m, $16H_{g-n}$); **^{13}C-NMR** (100 MHz, $CDCl_3$): δ (ppm) 172.6 (C=O), 169.6 (C=O), 168.7 (C=O), 166.2 (C=O), 164.2 (C=O), 143.4 (C), 136.6 (CH), 136.5 (C), 135.9 (C), 129.2 (CH), 128.9 (CH), 128.8 (CH), 128.1 (CH), 128.0 (CH), 127.8 (CH), 126.9 (CH), 126.9 (CH), 50.4 (CH_2), 48.7 (CH_2), 47.7 (CH), 43.7 (CH_2), 43.3 (CH_2), 42.6 (CH_2), 40.0 (CH_2), 39.8 (CH_2), 29.4 (CH_2), 29.3 (CH_2), 29.2 (CH_2), 29.1 (CH_2), 26.9 (CH_2), 26.9 (CH_2); **HRMS (ESI)** calcd for $C_{49}H_{62}N_5O_5$ $[M + H]^+$ 800.4745, found 800.4763.

3. Synthesis of macrocycle 3

Fragment S3

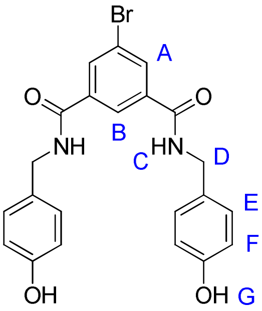

To a solution 4-hydroxybenzylamine (6.00 g, 48.88 mmol) and Et_3N (15.00 mL, 107.62 mmol) in dry THF (150 mL) was added dropwise a solution of 5-bromoisophthaloyl chloride (5.96 g, 24.44 mmol) in dry THF (20 mL). The reaction mixture was stirred for 12 hours under nitrogen atmosphere. After this time, the solution was filtered through Celite® and the solvent was concentrated under reduce pressure. The resulting mixture was dissolved in ethyl acetate (150 mL) and washed with a solution 1M HCl (4 x 80 mL). The organic phase was dried over anhydrous $MgSO_4$ and concentrated under reduced pressure. The residue was purified by column chromatography using a mixture $CHCl_3$/acetone 9/1 (v/v) as eluent. The product was obtained as a white solid (**S3**, 8.10 g, 17.84 mmol, 73 %); **m.p.** 83 – 84 ºC; **^{1}H-NMR** (400 MHz, DMSO-*d*$_6$): δ (ppm) 9.30 (s, 2H_G), 9.14 (t, *J* = 5.9 Hz, 2H_C), 8.35 (t, *J* = 1.5 Hz, 1H_B), 8.17 (d, *J* = 1.5 Hz, 2H_A), 7.11 (d, *J* = 8.5 Hz, 4H_E), 6.69 (d, *J* = 8.5 Hz, 4H_F), 4.34 (d, *J* = 5.9 Hz, 4H_D); **^{13}C-NMR** (100 MHz, DMSO-*d*$_6$): δ (ppm) 164.1 (C=O), 156.4 (C), 136.7 (C), 132.1 (CH), 129.4 (C), 128.8 (CH), 125.7 (CH), 121.6 (C), 115.0 (CH), 42.4 (CH_2); **HRMS (ESI)** calcd for $C_{22}H_{20}BrN_2O_4$ $[M + H]^+$ 455.0601 and 457.0583, found 455.0597 and 457.0579.

Macrocycle 3

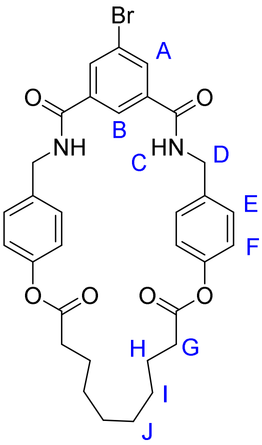

To a solution of compound **S3** (2.00 g, 4.40 mmol) and Et_3N (1.40 mL, 10.04 mmol) in dry THF (1 L) was added dropwise for 2 hours a solution of sebacoyl chloride (0.94 mL, 4.40 mmol) in dry THF (19 mL). The reaction mixture was stirred at room temperature for 24 hours. The resulting solution was filtered through Celite® and concentrated under reduced pressure. The mixture was dissolved in a mixture 3/1 chloroform/isopropanol 3/1 (v/v) (50 mL) and washed with a solution of 1M HCl (3 x 40 mL), a saturated solution of $NaHCO_3$ (3 x 40 mL) and brine (1 x 40 mL). The organic phase was dried over anhydrous $MgSO_4$, filtered and concentrated under reduced pressure. The residue was purified by column chromatography by using a mixture dichloromethane/ethyl acetate 8/2 (v/v) as eluent. The product was obtained as a white solid (**3**, 0.41 g, 0.66 mmol, 15%); **m.p.** 219 – 221 ºC; **^{1}H-NMR** (400 MHz, $CDCl_3$): δ (ppm) 8.12 (s, $2H_A$), 7.67 (s, $1H_B$), 7.33 (d, *J* = 8.3 Hz, $4H_E$), 7.05 (d, *J* = 8.3 Hz, $4H_F$), 6.48 (s, $2H_C$), 4.56 (d, *J* = 5.3 Hz, $4H_D$), 2.59 – 2.54 (m, $4H_G$), 1.74 – 1.69 (m, $4H_H$), 1.45 – 1.33 (m, $8H_{I+J}$); **^{13}C-NMR** (100 MHz, $CDCl_3$): δ (ppm) 173.1 (C=O), 166.1 (C=O), 151.2 (C), 137.4 (C), 135.9 (C), 134.7 (CH), 130.4 (CH), 124.6 (C), 123.3 (CH), 122.9 (CH), 44.9 (CH_2), 34.9 (CH_2), 30.1 (CH_2), 29.3 (CH_2), 25.8 (CH_2); **HRMS (ESI) calcd for** $C_{32}H_{34}BrN_2O_6$ $[M + H]^+$ 621.1600 and 623.1575, found 621.1586 and 623.1572.

4. Synthesis of macrocycle 4

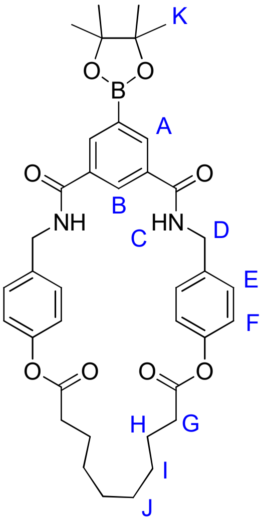

To a solution of macrocycle **3** (0.37 g, 0.60 mmol), bis(pinacolato)diboron (0.23 g, 0.90 mmol) and potassium acetate (0.18 g, 1.83 mmol) in dry dioxane (5.00 mL) under nitrogen atmosphere was added Pd(dppf)Cl_2 (0.03 g, 5% mmol). The reaction mixture was stirred for 24 hours at 90 ºC. After this time, chloroform (25 mL) was added and the solution was filtered through Celite®. The filtrate was washed with brine (2 x 15 mL). The organic phase was dried over anhydrous $MgSO_4$, filtered and concentrated under reduced pressure. The resulting solid was washed several times with pentane to obtain a white solid (**4**, 0.32 g, 0.48 mmol, 80%); **m.p.** 45 – 47 ºC; **^{1}H-NMR** (300 MHz, $CDCl_3$): δ (ppm) 8.38 (d, *J* = 1.7 Hz, $2H_A$), 7.93 (t, *J* = 1.7 Hz, $1H_B$), 7.33 (d, *J* = 8.5 Hz, $4H_E$), 7.04 (d, *J* = 8.5 Hz, $4H_F$), 6.48 (t, *J* = 5.4 Hz, $2H_C$), 4.56 (d, *J* = 5.4 Hz, $4H_D$), 2.58 – 2.51(m, $4H_G$), 1.77–

1.67 (m, $4H_H$), 1.35 (s, $12H_K$), 1.28 – 1.23 (m, $8H_{I+J}$); **^{13}C-NMR** (75 MHz, $CDCl_3$): δ (ppm) 172.3 (C=O), 166.9 (C=O), 150.4 (C), 136.5 (CH), 135.7 (C), 134.5 (C), 129.5 (CH), 127.4 (CH), 122.1 (CH), 84.6 (C), 43.9 (CH_2), 34.2 (CH_2), 29.8 (CH_2), 29.6 (CH_2), 28.8 (CH_2), 28.2 (CH_2), 25.0 (CH_3); **HRMS (MS)** calcd for $C_{38}H_{46}BN_2O_8$ $[M + H]^+$ 668.3378, found 668.3395.

NOTE: Macrocycle **4** showed to be unstable when submitted under column chromatography conditions.

5. Synthesis of [2]rotaxane 2 and [3]rotaxane S4

To a solution of thread **1** (0.50 g, 0.63 mmol), *N*[1],*N*[3]-bis(4-(aminomethyl)benzyl)isophthalamide[2] (0.89 g, 2.20 mmol) and Et_3N (2.00 mL, 14.35 mmol) in chloroform (300 mL) under nitrogen atmosphere was added dropwise a solution of 5-bromoisophtaloyl dichloride (0.62 g, 2.20 mmol) in chloroform (20 mL) for a period of 5 hours. The reaction mixture was stirred for additional 24 hours and then filtered through Celite® and washed with water (2 x 200 mL), 1M NaOH (2 x 200 mL), 1M HCl (2 x 200 mL) and brine (200 mL). The organic phase was dried over anhydrous $MgSO_4$, filtered and concentrated under reduced pressure. The resulting residue was purified by column chromatography using a mixture of chloroform/methanol 95/5 (v/v) as eluent, obtaining [3]rotaxane **S4** (0.051 g, 0.025 mmol, 4%) and [2]rotaxane **2** (0.48 g, 0.33 mmol, 53%) as white solids.

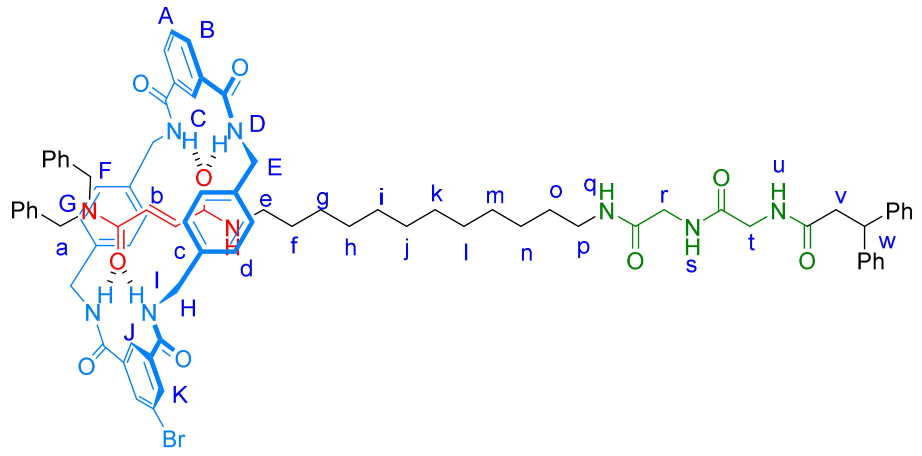

__[2]rotaxane 2__: **m.p.** 92 – 94 °C; **^{1}H-NMR** (400 MHz, $CDCl_3$): δ (ppm) 8.46 (s, $1H_C$), 8.42 (s, $1H_J$), 8.26 (d, *J* = 1.1 Hz, $2H_K$), 8.17 (dd, *J* = 7.8, 1.3 Hz, $2H_B$), 7.73 (s, $1H_d$), 7.65 – 7.55 (m, $5H_{A+D+I}$), 7.36 – 7.08 (m, $18H_{Ar}$), 6.99 – 6.91 (m, $8H_{F+G}$), 6.74 – 6.66 (m, $3H_{Ar+u}$), 6.46 (s, $1H_q$), 6.07 – 5.93 (m, $2H_{b+c+s}$), 4.65 – 4.15 (m, $13H_{E+H+a+w}$), 3.68 (d, *J* = 5.0 Hz, $2H_t$), 3.55 (d, *J* = 5.7 Hz, $2H_r$), 3.23 – 3.11 (m, $4H_{e+p}$), 3.02 (d, *J* = 8.1 Hz, $2H_v$), 1.58 – 1.44 (m, $4H_{f+o}$), 1.40 – 1.19 (m, $16H_{g-n}$); **^{13}C-NMR** (100 MHz, $CDCl_3$): δ (ppm) 172.8 (C=O), 169.5 (C=O), 168.4 (C=O), 166.7 (C=O), 165.9 (C=O), 165.4 (C=O), 164.5 (C=O), 143.1 (C), 137.6 (C), 137.2 (C), 135.9 (C), 135.4 (C), 134.8 (CH), 134.5 (C), 133.7 (CH), 131.8 (CH), 129.3 (CH), 129.2 (CH), 129.1 (CH), 129.9 (CH), 128.9 (CH), 128.7 (CH), 128.4 (CH), 127.8 (CH), 126.9 (CH), 125.5 (CH), 124.2 (CH), 123.8 (CH), 122.0 (CH), 51.2 (CH_2), 50.8 (CH_2), 47.6 (CH), 44.2 (CH_2), 43.6 (CH_2), 43.2 (CH_2), 42.7 (CH_2), 40.5 (CH_2), 39.8 (CH_2),

29.9 (CH_2), 29.3 (CH_2), 29.1 (CH_2), 29.0 (CH_2), 28.8 (CH_2), 26.9 (CH_2), 26.5 (CH_2); **HRMS (MS)** calcd for $C_{81}H_{89}BrN_9O_9$ $[M + H]^+$ 1410.5961 and 1412.5966, found 1410.5985 and 1412.5993.

[3]rotaxane S4:

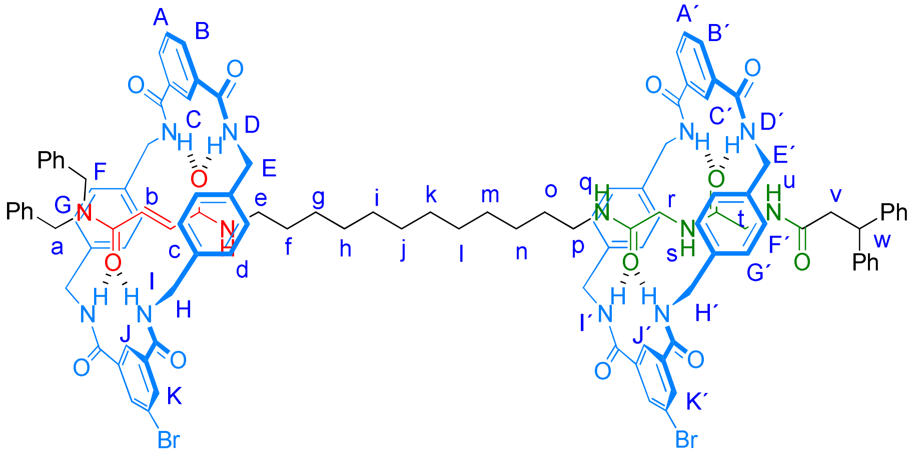

m.p. 146 – 148 ºC; **^{1}H-NMR** (400 MHz, $CDCl_3$): δ (ppm) 8.40 (s, $1H_d$), 8.35 (s, $2H_{C+C'}$), 8.22 – 8.18 (m, $6H_{J+J'+K+K'}$), 8.08 – 8.02 (m, $4H_{B+B'}$), 7.87 (t, *J* = 4.7 Hz, $2H_D$), 7.77 (t, *J* = 4.5 Hz, $2H_{D'}$), 7.61 – 7.55 (m, $4H_{I+I'}$), 7.52 – 7.45 (m, $2H_{A+A'}$), 7.34 –7.03 (m, $28H_{Ar+F'+G'+q+u}$), 6.99 – 6.87 (m, $8H_{F+G}$), 6.89 (s, $1H_s$), 6.65 (d, *J* = 7.3 Hz, $2H_{Ar}$), 6.06 (d, *J* = 14.7 Hz, $1H_b$), 5.93 (d, *J* = 14.7 Hz, $1H_c$), 4.60 – 4.21 (m, $21H_{a+E+H+E'+H'+w}$), 3.31 (q, *J* = 5.8 Hz, $2H_e$), 3.15 (d, *J* = 3.7 Hz, $2H_t$), 3.02 (d, *J* = 2.7 Hz, $2H_r$), 2.87 – 2.77 (m, $4H_{p+v}$), 1.64 – 1.56 (m, $2H_f$), 1.44 – 1.35 (m, $2H_o$), 1.60 – 1.17 (m, $16H_{g-n}$); **^{13}C-NMR** (100 MHz, $CDCl_3$): δ (ppm) 172.6 (C=O), 170.0 (C=O), 168.3 (C=O), 166.9 (C=O), 166.4 (C=O), 166.03 (C=O), 165.7 (C=O), 165.5 (C=O), 164.6 (C=O), 143.4 (C), 137.4 (C), 137.2 (C), 135.9 (C), 135.8 (C), 135.2 (C), 134.6 (CH), 134.4 (C), 134.3 (CH), 134.0 (CH), 133.9 (C), 133.5 (C), 131.6 (CH), 131.3 (CH), 129.4 (CH), 129.2 (CH), 129.1 (CH), 128.9 (CH), 128.8 (CH), 128.6 (CH), 128.5 (CH), 128.4 (CH), 127.7 (CH), 126.8 (CH), 126.0 (CH), 125.6 (CH), 124.8 (CH), 123.9 (CH), 123.7 (CH), 123.5 (CH), 122.1 (CH), 51.4 (CH_2), 51.0 (CH_2), 47.2 (CH), 44.5 (CH_2), 44.1 (CH_2), 43.5 (CH_2), 43.2 (CH_2), 42.5 (CH_2), 42.1 (CH_2), 40.5 (CH_2), 39.8 (CH_2), 29.8 (CH_2), 29.5 (CH_2), 29.4 (CH_2), 29.4 (CH_2), 29.3 (CH_2), 29.3 (CH_2), 29.0 (CH_2), 27.2 (CH_2), 26.8 (CH_2); **HRMS (ESI):** calcd for $C_{113}H_{116}Br_2N_{13}O_{13}$ $[M + H]^+$ 2020.7177, found 2020.7278.

6. Synthesis of [2]rotaxane 5

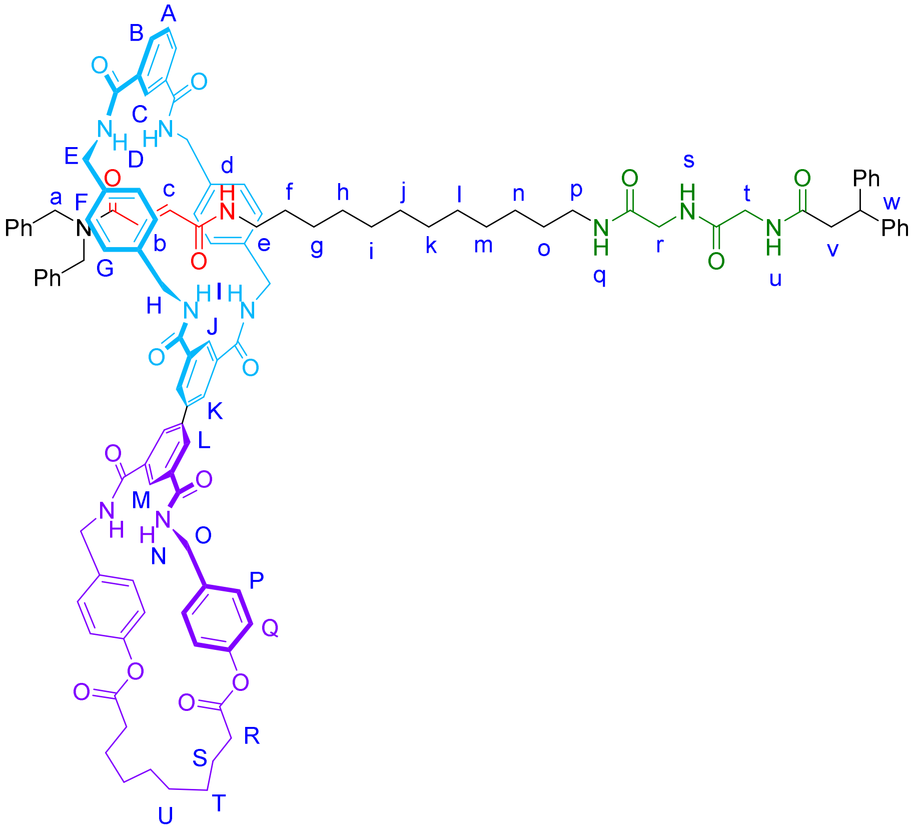

A solution of [2]rotaxane **2** (22.00 mg, 0.016 mmol), macrocycle **4** (16.00 mg, 0.024 mmol), K_3PO_4 (8.50 mg, 0.04 mmol) and $Pd(PPh_3)_4$ (3.70 mg, 20% mmol) in dry dioxane (0.6 mL) was stirred under nitrogen atmosphere at 100 ºC for 24 hours. After this time, chloroform (15 mL) was added and the mixture was filtered through Celite®. The filtrate was washed with water (2 x 10 mL) and brine (10 mL). The organic phase was dried over anhydrous $MgSO_4$, filtered and concentrated under reduced pressure. The resulting residue was purified by column chromatography using a mixture of chloroform/ethanol 95/5 (v/v) as eluent, where the aliquots containing the desired compound **5** were collected, mixed with other byproducts. This fraction was then submitted to a size exclusion chromatography with TOPOYEARL HMW Size Exclusion Sampler (HW-55, 65, 75F) as stationary phase using a mixture of chloroform/methanol 1/1 (v/v) as eluent. The product was obtained as a white solid (**5**, 2.43 mg, 0.0013 mmol, 8%); **m.p.** 150 – 152 ºC; **^{1}H-NMR** (400 MHz, DMSO-d_6): δ (ppm) 9.16 (t, J = 5.6 Hz, 2H_N), 8.67 (t, J = 5.5 Hz, 2H_D), 8.50 (t, J = 5.4 Hz, 2H_I), 8.39-8.23 (m, 8$H_{C+J+K+L+M+d}$), 8.15 (t, J = 5.5 Hz, 1H_u), 7.99 (d, J = 7.6 Hz, 2H_B), 7.78 (t, J = 5.3 Hz, 1H_s), 7.59-7.51 (m, 2H_{A+q}), 7.39 (d, J = 8.5 Hz, 4H_P), 7.34-7.10 (m, 20H_{Ar}), 7.07-6.97 (m, 12H_{F+G+Q}), 6.70 (d, J = 14.8 Hz, 1H_b), 6.57 (d, J = 14.8 Hz, 1H_c), 4.55-4.25 (m, 17$H_{E+H+O+a+w}$), 3.56 (d, J = 5.5 Hz, 2H_t), 3.50 (d, J = 5.3 Hz, 2H_r), 2.89 (d, J = 7.9 Hz, 2H_v), 2.88-2.82 (m, 2H_e), 2.78-2.72 (m, 2H_p), 2.60-2.54 (m, 4H_R), 1.63-1.56 (m, 4H_S), 1.35-1.27 (m, 12H_{T-U}), 1.16-1.06 (m, 4H_{f+o}), 0.93-0.79 (m, 16H_{g-n}); **^{13}C-NMR** (100 MHz, DMSO-d_6): δ (ppm) 172.0 (C=O), 170.9 (C=O), 169.3 (C=O), 168.3 (C=O), 166.1 (C=O), 165.6 (C=O), 165.3 (C=O), 164.1 (C=O) , 149.5 (C), 144.4 (C), 139.3 (C), 137.5 (C), 137.2

(C), 136.8 (C), 135.8 (C), 135.3 (C), 135.0 (C), 134.3 (C), 130.2 (CH), 129.1 (CH), 128.6 (CH), 128.5 (CH), 128.4 (CH), 128.2 (CH), 127.5 (CH), 127.4 (CH), 126.1 (CH), 121.7 (CH), 50.4 (CH_2), 49.4 (CH_2), 46.5 (CH), 43.0 (CH_2), 42.6 (CH_2), 42.2 (CH_2), 41.9 (CH_2), 33.2 (CH_2), 30.8 (CH_2), 29.0 (CH_2), 28.8 (CH_2), 28.7 (CH_2), 27.9 (CH_2), 26.4 (CH_2), 26.3 (CH_2), 24.4 (CH_2); **HRMS (ESI)** calcd for $C_{113}H_{122}N_{11}O_{15}$ [M + H]$^+$ 1872.9116, found 1872.9122.

7. Controlled disassembly and assembly of 5·5 through DA and retro-DA reactions

A solution of the corresponding thread or rotaxane (1 eq) in 0.5 mL of DMSO was treated with freshly cracked and distilled cyclopentadiene (10 equiv.) under N_2 atmosphere. The mixture was heated at 80 ºC for 16 h. After this time, the reaction mixture was extracted with $CHCl_3$ and washed with a saturated NaCl solution. The resulting residue was purified by column chromatography using a mixture of chloroform/methanol 97/3 (v/v).

Thread *Cp*-1

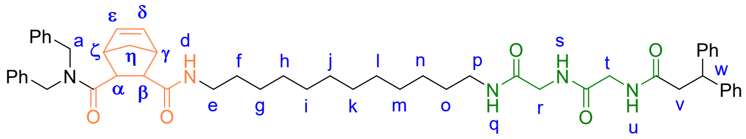

Thread ***Cp*-1** was synthesized following the general procedure described above using thread **2** (26 mg, 0.03 mmol) and cyclopentadiene (19.8 mg, 0.30 mmol), obtaining the tittle product as a white solid (23 mg, 88%) after purification by preparative TLC using a mixture chloroform/methanol 98/2 (v/v) as eluent; **m.p.** 71 – 73 ºC; **^{1}H-NMR** (400 MHz, $CDCl_3$): δ (ppm) 7.37 – 7.11 (m, 42H, $H_{Ar+Ar'}$+NH), 6.72 – 6.66 (m, 2H, NH), 6.33 (dd, *J* = 5.5, 3.1 Hz, 1H, H_{δ}), 6.28 – 6.19 (m, 2H, NH), 6.17 (dd, *J* = 5.6, 3.1 Hz, 1H, $H_{\delta'}$), 6.10 – 6.05 (m, 3H, $H_{\varepsilon+\varepsilon'}$+$NH_d$), 5.90 – 5.80 (m, 1H, $NH_{d'}$), 4.82 – 4.30 (m, 10H, $H_{a+a'+w+w'}$), 3.72 (d, *J* = 5.1 Hz, 4H, $H_{t+t'}$), 3.70 – 3.67 (m, 2H, H_{β}), 3.62 (d, *J* = 5.8 Hz, 4H, $H_{r+r'}$), 3.48 – 3.43 (m, 2H, $H_{\beta'}$), 3.25 – 2.76 (m, 18H, $H_{e+e'+p+p'+v+v'+\zeta+\zeta'+\gamma+\gamma'+\alpha+\alpha'}$), 2.00 (d, *J* = 8.3 Hz, 1H, H_{η}), 1.93 (d, *J* = 8.4 Hz, 1H, $H_{\eta'}$), 1.51 – 1.13 (m, 42 H, $H_{f\text{-}o+f'\text{-}o'+n+n'}$); **^{13}C-NMR** (100 MHz, $CDCl_3$): δ (ppm) 13C NMR (101 MHz, CDCl3) δ 175.0 (CO), 174.3 (CO), 173.7 (CO), 172.9 (CO), 172.8 (CO), 169.4 (CO), 168.7 (CO), 143.3 (C), 138.0 (C), 137.3 (C), 137.2 (C), 136.7 (C), 136.5 (C), 135.1 (C), 133.8 (C), 129.1 (CH), 129.0 (CH), 128.8 (CH), 127.9 (CH), 127.8 (CH), 127.5 (CH), 127.0 (CH), 126.9 (CH), 50.3 (CH_2), 49.9 (CH_2), 49.5 (CH), 48.9 (CH), 48.8 (CH), 48.6 (CH), 48.5 (CH_2), 48.2 (CH_2), 48.0 (CH_2), 47.7 (CH), 47.6 (CH_2), 46.8 (CH), 46.7 (CH), 46.0 (CH), 44.8 (CH), 43.7 (CH_2), 43.1 (CH_2), 42.5 (CH_2), 39.9 (CH_2), 39.8 (CH_2), 39.7 (CH_2), 29.7 (CH_2), 29.5 (CH_2), 29.4 (CH_2), 29.3 (CH_2), 26.9 (CH_2); **HRMS (ESI)** calcd for $C_{54}H_{68}N_5O_5$ [M + H]$^+$ 866.5215, found 866.5223.

Rotaxane *Cp*-2

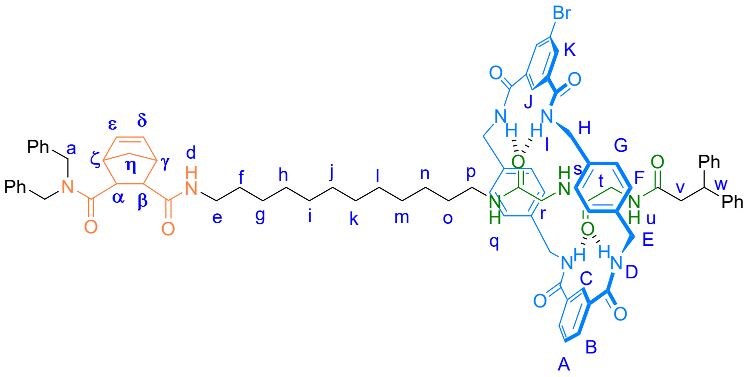

Rotaxane ***Cp*-2** was synthesized following the general procedure described above using rotaxane **2** (40 mg, 0.028 mmol) and cyclopentadiene (19 mg, 0.28 mmol), obtaining the tittle product as a white solid (30 mg, 72%) after purification by preparative TLC using a mixture chloroform/methanol 98/2 (v/v) as eluent; **m.p.** 116 – 118 ºC; **^{1}H-NMR** (400 MHz, $CDCl_3$): δ (ppm) 8.31 – 8.27 (m, 4H, $H_{C+C'+J+J'}$), 8.22 (s, 4H, $H_{K+K'}$), 8.07 (d, *J* = 7.7 Hz, 4H, $H_{B+B'}$), 7.95 – 7.73 (m, 8H, $H_{D+D'+I+I'}$), 7.50 (t, *J* = 7.7 Hz, 2H, $H_{A+A'}$), 7.40 – 6.96 (m, 58H, $H_{F+F'+G+G'+Ar+Ar'}$+ NH), 6.80 (s, 2H, NH), 6.50 (s, 2H, NH), 6.35 – 6.28 (m, 1H, H_{δ}), 6.17 – 6.11 (m, 1H, $H_{\delta'}$), 6.09 – 6.03 (m, 2H, $H_{\varepsilon+\varepsilon'}$), 5.98 (t, *J* = 5.4 Hz, 1H, NH_d), 5.85 (t, *J* = 5.5 Hz, 1H, NH_d), 4.84 – 4.23 (m, 24H, $H_{a+a'E+E'+H+H'}$), 4.18 (t, *J* = 7.7 Hz, 2H, $H_{w+w'}$), 3.65 – 3.61 (m, 2H, H_{β}), 3.47 – 3.43 (m, 2H, $H_{\beta'}$), 3.23 – 2.83 (m, 22H, $H_{r+r'+t+t'+e+e'+p+p'+\alpha+\alpha'+\gamma+\gamma'+\zeta'+\zeta}$), 2.68 (d, *J* = 7.7 Hz, 4H, $H_{v+v'}$), 1.97 (d, *J* = 8.3 Hz, 1H, H_{η}), 1.90 (d, *J* = 8.4 Hz, 1H, $H_{\eta'}$), 1.48 – 1.04 (m, 42 H, $H_{f-o+f'-o'+\eta+\eta'}$); **^{13}C NMR** (100 MHz, $CDCl_3$) δ 175.0 (CO), 174.5 (CO), 173.5 (CO), 173.1 (CO), 172.6 (CO), 169.9 (CO), 168.2 (CO), 166.9 (CO), 165.5 (CO), 143.3 (C), 138.0 (CH), 137.5 (C), 137.4 (CH), 137.3 (C), 137.2 (C), 136.7 (C), 136.5 (C), 135.9 (C), 135.1 (CH), 134.3 (CH), 134.1 (C), 133.9 (CH), 131.3 (CH), 129.1 (CH), 129.1 (CH), 129.0 (CH), 128.9 (CH), 128.8 (CH), 128.7 (CH), 128.0 (CH), 127.9 (CH), 127.8 (CH), 127.7 (CH), 127.6 (CH), 127.5 (CH), 127.0 (CH), 126.8 (CH), 125.0 (CH), 123.6 (CH), 123.5 (C), 50.3 (CH_2), 49.9 (CH_2), 49.6 (CH), 48.9 (CH), 48.7 (CH), 48.6 (CH_2), 48.6 (CH_2), 48.3 (CH_2), 48.0 (CH_2), 47.6 (CH_2), 47.2 (CH), 46.7 (CH), 46.6 (CH), 46.3 (CH), 44.9 (CH), 44.5 (CH_2), 44.4 (CH_2), 43.2 (CH_2), 42.5 (CH_2), 42.1 (CH_2), 39.9 (CH_2), 39.8 (CH_2), 39.7 (CH_2), 29.7 (CH_2), 29.5 (CH_2), 29.3 (CH_2), 29.2 (CH_2), 27.0 (CH_2), 26.9 (CH_2); **HRMS (ESI)** calcd for $C_{86}H_{95}BrN_9O_9$ $[M + H]^+$ 1476.6431 and 1478.6438, found 1476.6427 and 1478.6442.

Rotaxane *Cp*-5

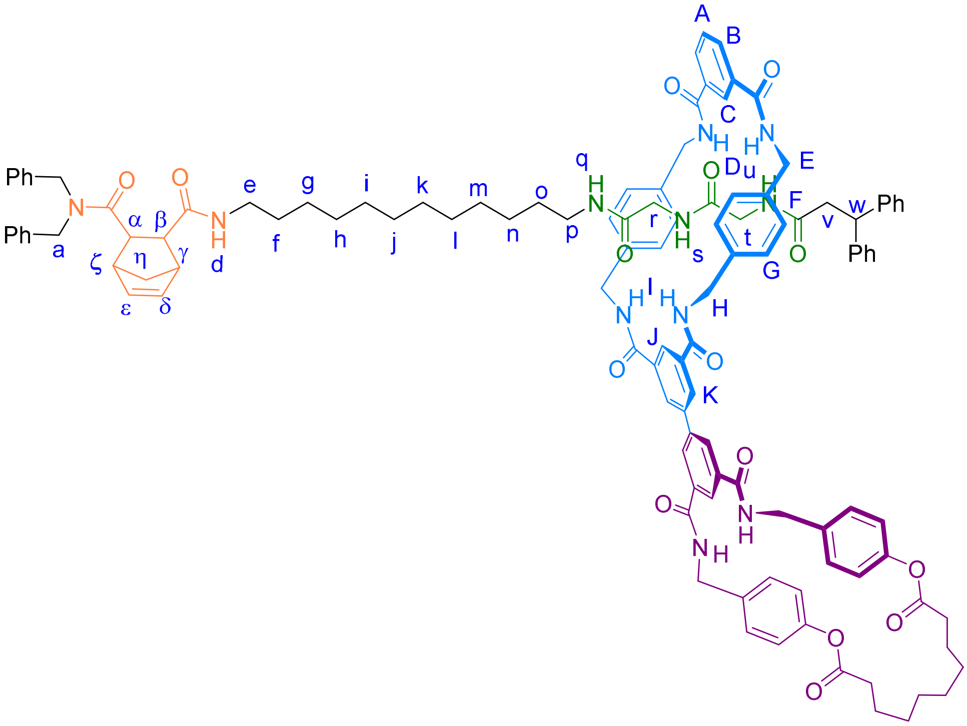

Rotaxane ***Cp*-5** was synthesized following the general procedure described above using rotaxane **5** (12 mg, 0.006 mmol) and cyclopentadiene (3.9 mg, 0.06 mmol), obtaining the tittle product as a white solid (6 mg, 48%) after purification by preparative TLC using a mixture chloroform/methanol 98/2 (v/v) as eluent. By using 1,1,2,2-tetrachloroethane as solvent ***Cp*-5** was also obtained but it needed a heating time of three days to afford the title product in 41% yield. **M.p.** 191 – 193 ºC; **^{1}H-NMR** (400 MHz, $CDCl_3$): δ (ppm) 8.47 (s, 1H, H_{M}), 8.29 – 8.08 (m, 12H, $H_{C+C'+J+M'+Q+Q'}$), 7.87 – 7.53 (m, 14H, $H_{J'+Ar+Ar'+A+A'+B+B'}$), 7.35 – 6.76 (m, 76H, $H_{Ar+Ar'+F+F'+G+G'+P+P'+I+I'+u+u'+D+D'+s+s'}$), 6.32 – 5.67 (m, 8H, $H_{q+q'+d+d'+\delta+\delta'+\varepsilon+\varepsilon'}$), 4.78 – 4.11 (m, 34H, $H_{E+E'+R+R'+H+H'+O+O'+w+w'}$), 3.65 – 2.54 (m, 28H, $H_{r+r'+t+t'+e+e'+p+p'+a+a'+\gamma+\gamma'+\zeta'+\zeta}$), 1.99 – 1.96 (m, 1H, H_{α}), 1.91 – 1.97 (m, 1H, H_{β}), 1.75 – 1.09 (m, 77H, $H_{S+T+U+S'+U'+T'+\alpha'+\beta'+f-o+f'-o'+\eta+\eta'+N+N'}$); **^{13}C NMR** (100 MHz, $CDCl_3$) δ 175.0 (CO), 174.5 (CO), 172.4 (CO), 169.8 (CO), 166.9 (CO), 166.5 (CO), 150.3 (C), 143.5 (C), 137.4 (C), 134.2 (CH), 131.5 (C), 129.79 (CH), 129.4 (CH), 129.1 (CH), 128.8 (CH), 128.1 (CH), 127.7 (CH), 127.5 (CH), 127.1 (CH), 126.9 (CH), 122.0 (CH), 70.6 (C), 50.3 (CH_2), 49.9 (CH_2), 49.0 (CH_2), 48.6 (CH_2), 47.2 (CH_2), 46.6 (CH_2), 46.4 (CH_2), 44.4 (CH_2), 43.8 (CH_2), 42.9 (CH_2), 42.5 (CH_2), 42.2 (CH_2), 39.9 (CH_2), 39.8 (CH_2), 34.2 (CH_2), 31.1 (CH_2), 29.8 (CH_2), 29.7 (CH_2), 29.5 (CH_2), 29.3 (CH_2), 28.8 (CH_2), 26.9 (CH_2), 25.2 (CH_2), 19.3 (CH_2); **HRMS (ESI)** calcd for $C_{118}H_{126}N_{11}O_{15}$ $[M - H]^{-}$ 1936.9440, found 1936.9367.

The retro-Diels-Alder reaction of rotaxane ***Cp*-5** was accomplished in FVP equipment, heating a solid sample of ***Cp*-5** up to 235 °C during 30 min, keeping a vacuum of 0.5 Torr. The corresponding rotaxane **5** was isolated in moderate yield (41%) after purification by preparative TLC using a mixture

chloroform/methanol 98/2 (v/v) as eluent. This reaction condition was optimized for minimizing the degradation products observed at higher temperatures and shorter times.

8. Synthesis of pyridine *N*-oxide derivative 6

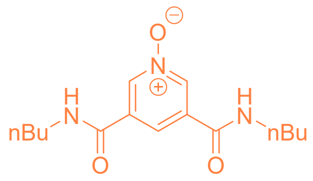

Pyridine *N*-oxide derivative **6** was synthesized following the procedure described in bibliography and showed identical spectroscopic data as those reported therein.[3]

9. DOSY NMR experiments

NMR diffusion measurements were performed on a 600 MHz Bruker AVANCE spectrometer. The samples were not spinned and the airflow was disconnected. The *D*-values were determined from the slope of the regression line $\ln(I/I_o)$ vs G^2, according to the following equation:[4]

$$ln(I/I_o) = -(\gamma\delta)^2 G^2(\Delta-\delta/3)D$$

I/I_o = observed spin echo intensity/intensity without gradients, G = gradient strength, Δ = delay between the midpoints of the gradients, D = diffusion coefficient, δ = gradient length of the pulse, γ = magnetogyric ratio.

The calibration of the gradients was carried out via a diffusion measurement of HDO in D_2O (D_{HDO} = $1.9\cdot10^{-9}$ m^2 s^{-1}).[5] The values reported in Table 1 are the average of three different measurements (Δ = 50, 150 and 400 ms), which yielded *D*-values within max. ±2.0% of the reported one. All the measurements were carried out using the ^{1}H resonances. The gradient length was set in the range of 1.2 and 10.9 ms. In regard to the values reported in Table S2, they were obtained with Δ = 150 and 400 ms (except data from entries 6 and 7 which are the result of one measurement with Δ = 150 ms), which yielded *D*-values within max. ± 4.0% of the reported one. The gradient length was set in the range of 1.5 and 3.0 ms. For all the experiments the number of scans was 32 and the experimental time was ca. 90 min. All the observed data leading to the reported *D*-values afforded lines whose correlation coefficients were above 0.999.

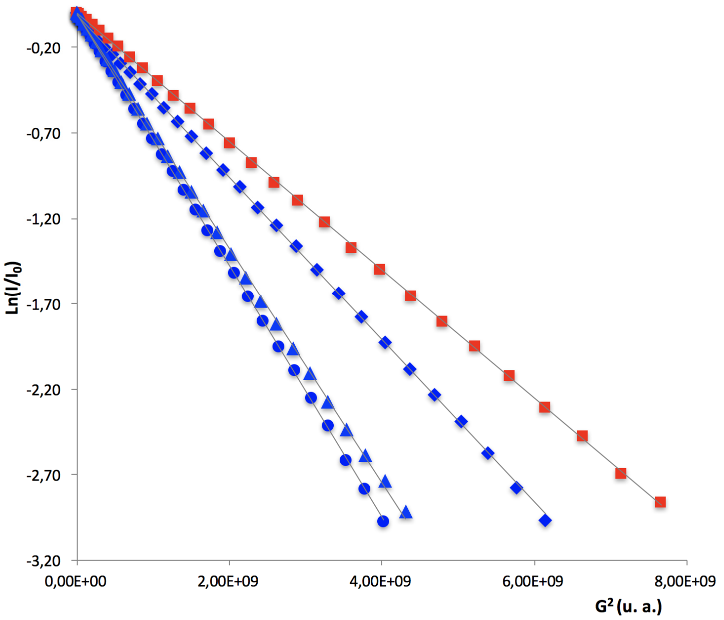

Figure S1. *Plot of the observed intensity changes ln(I/I_0) as a function of G^2 (arbitrary units) showing the different translation rates depending on their molecular sizes for the NMR diffusion measurements of solutions of macrocycle* ***3*** *(●), thread* ***1*** *(▲), rotaxane* ***2*** *(◆) and rotaxane* ***5*** *(■). The calculated lines have been adjusted to compensate the different values of Δ and δ (600 MHz, $2x10^{-3}$ M, $CDCl_3$, 298 K).*

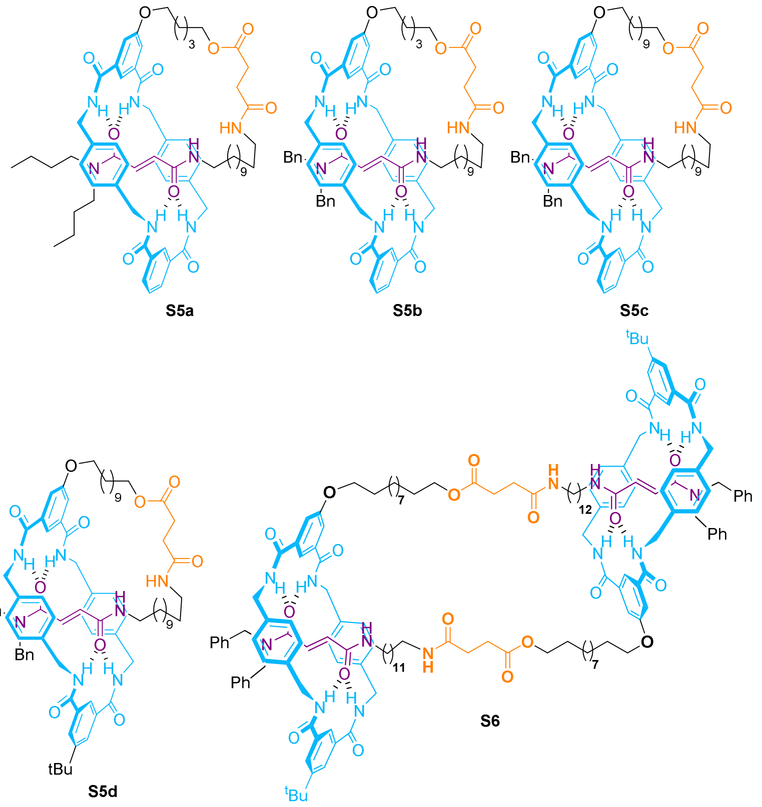

Figure S2. *Structures of described molecular lassos* ***S5a-d*** *and daisy chain* ***S6***.[6]

Table S1. Diffusion coefficients, **D** ($m^2 s^{-1}$) for 2 x 10^{-3} M solutions of selected compounds in $CDCl_3$ at 298 K and their corresponding molecular weights, Mw ($g{\cdot}mol^{-1}$).

Entry	**Compound**	**M_W**	**D /10^{-10}**	**log Mw**	**log D**
1	**2**	1411.6	4.76	3.1497116	-9.3223930
2	**S5a**	1126.5	5.67[a]	3.0517312	-9.2464169
3	**S5b**	1194.5	5.31[a]	3.0771862	-9.2749055
4	**S5c**	1278.6	5.10[a]	3.1067347	-9.2924298
5	**S5d**	1334.8	5.04[b]	3.1254162	-9.2975695
6	**S6**	2669.5	3.74[b]	3.4264299	-9.4271284

[a] *Previously described in the literature (see ref. 6a).* [b] *Previously described in the literature (see ref. 6b).*

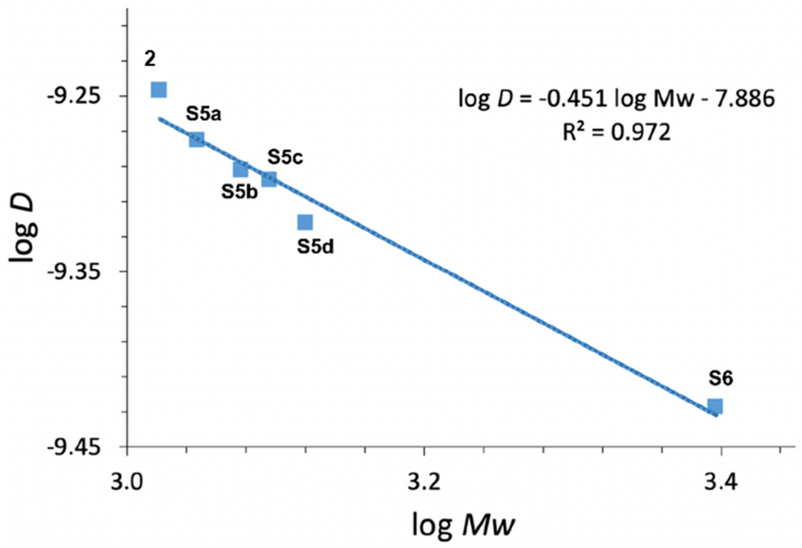

Figure S3. *Plot of the logarithm of the molecular weights of rotaxane* ***2*** *and the previously described hydrogen-bonded rotaxanes* ***S5a-d*** *and* ***S6*** *versus the logarithm of their respective diffusion coefficients.*[6]

The rotational radius (r_{Rot}) of macrocycle **3** was calculated from the minimized structure (BP86-D3/def2-SVP theoretical level) by using the Spartan'16 software, obtaining a value of 8.4 Å.

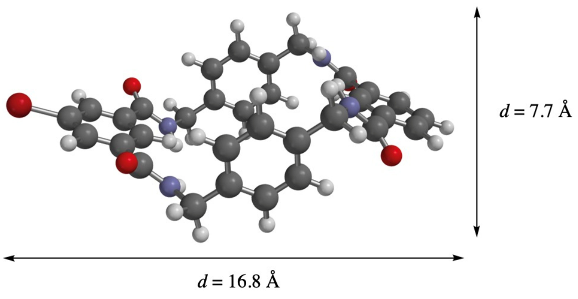

Figure S4. *Computed structure of macrocycle* ***3****.*

The rotational radius (r_{Rot}) of thread **1** was calculated from the minimized structure (BP86-D3/def2-SVP theoretical level) by using the Spartan'16 software, obtaining a value of 7.6 Å.

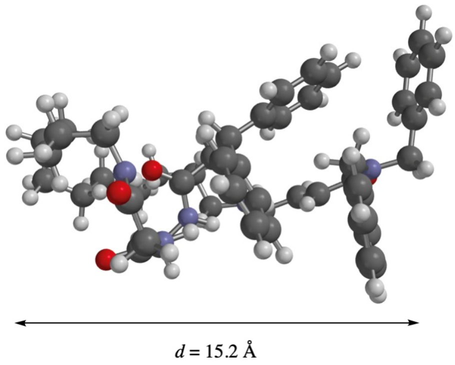

Figure S5. *Computed structure of thread **1**.*

The rotational radius (r_{Rot}) of [2]rotaxane **2** was calculated from the minimized structure (BP86-D3/def2-SVP theoretical level) by using the Spartan'16 software, obtaining a value of 8.8 Å.

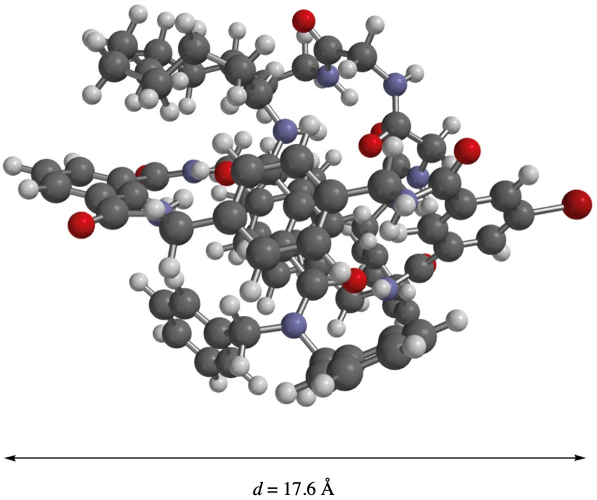

Figure S6. *Computed structure of [2]rotaxane **2**.*

The rotational radius (r_{Rot}) of [2]rotaxane **5** was calculated from the minimized structure (BP86-D3/def2-SVP theoretical level) by using the Spartan'16 software, obtaining a value of 10.9 Å.

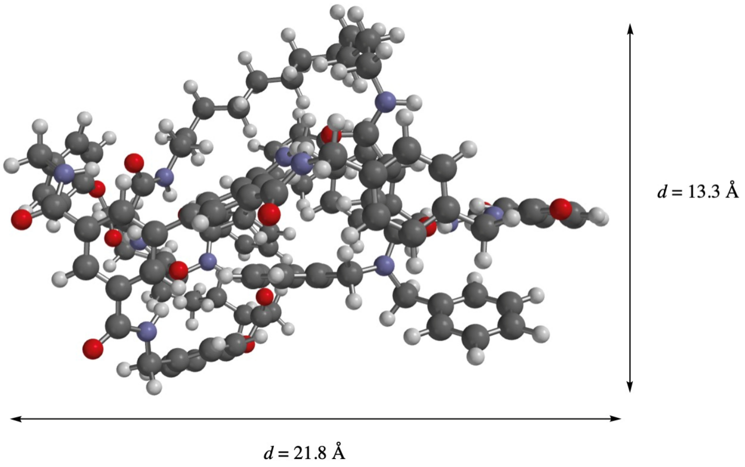

Figure S7. *Computed structure of [2]Rotaxane **5**.*

The rotational radius (r_{Rot}) of [4]rotaxane **5·5** was calculated from the minimized structure (BP86-D3/def2-SVP theoretical level) by using the Spartan'16 software, obtaining a value of 17.8 Å.

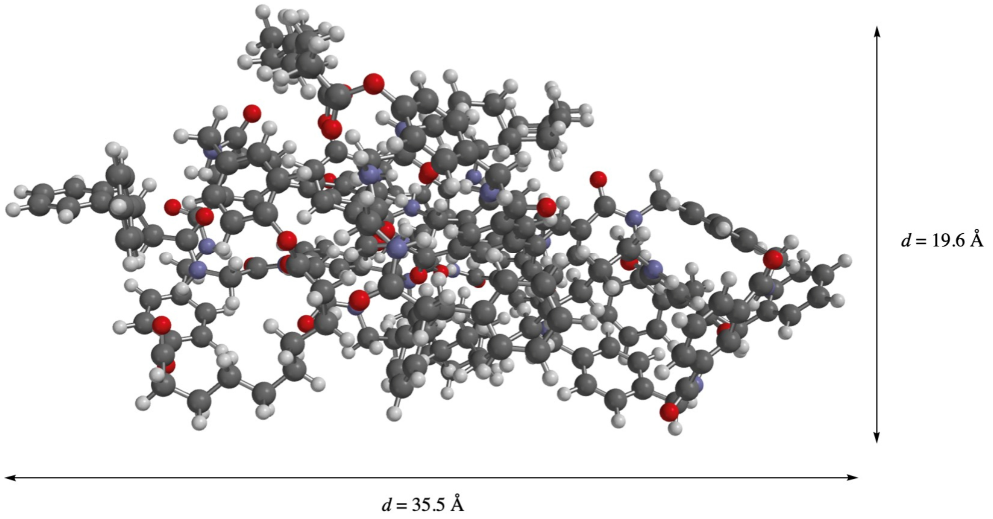

Figure S8. *Computed structure of [4]Rotaxane **5·5**.*

Table S2. Diffusion coefficients, D ($m^2 s^{-1}$) for solutions of **2** and **5** at different concentrations ($CDCl_3$, 298 K).

Entry	**Compound**	**Concentration (M)**	**δ (ppm)**	**D [a]/10^{-10}**
1	**2**	0.010	8.26, 4.36	4.32[b]
2	**2**	0.002	8.26, 4.36	4.76[c]
3	**5**	0.010	8.16, 2.58	3.38[b,d]
4	**5**	0.008	8.16, 2.58	3.49[b,d]
5	**5**	0.006	8.16, 2.58	3.55[b,d]
6	**5**	0.004	8.16, 2.58	3.62[d,e]
7	**5**	0.002	8.16, 2.58	3.71[d,e]

[a] Experimental error: ± 4.0%. [b] The diffusion coefficient was the average of two different measurements. [c] The diffusion coefficient is the same value shown in Table 1. [d] The formation of a tiny amount of a white precipitate (less than1%) was observed over time, probably due to the formation of polymeric species. [e] The diffusion coefficient was determined by only one measurement.

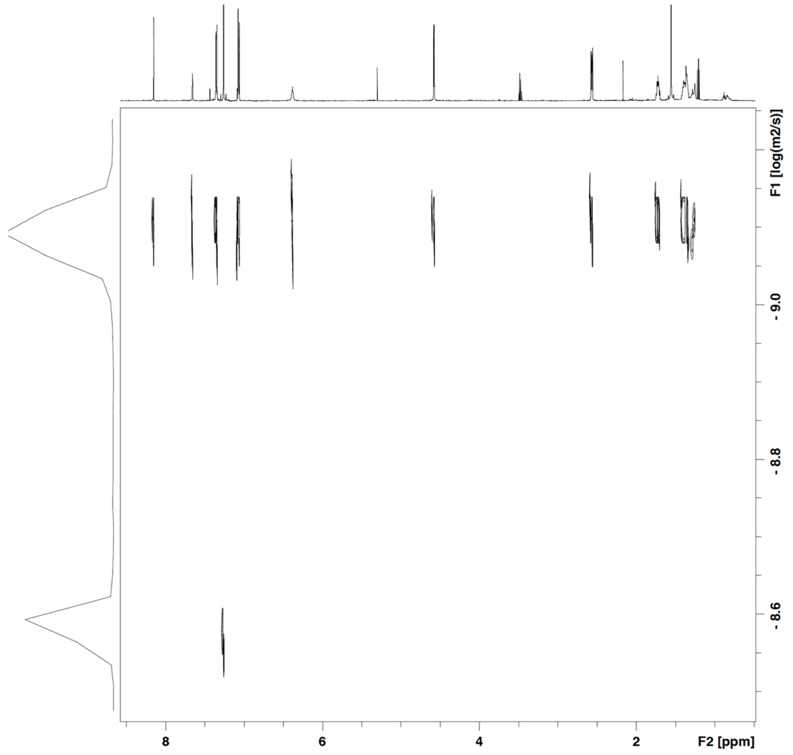

Figure S9. *DOSY 2D (600 MHz, 2 x 10^{-3} M, $CDCl_3$, 298 K) of macrocycle* ***3****.*

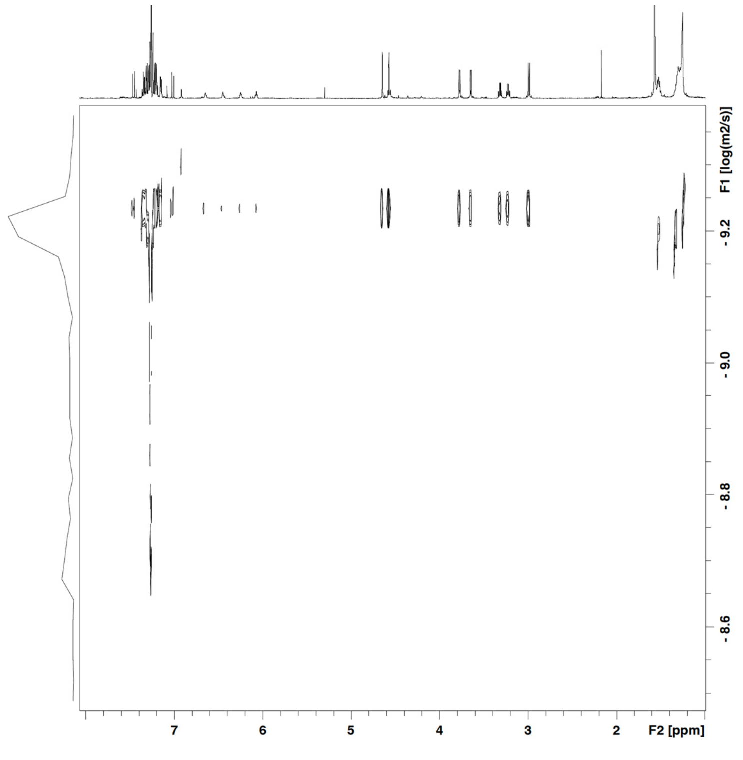

Figure S10. *DOSY 2D (600 MHz, 2 x 10^{-3} M, $CDCl_3$, 298 K) of thread* ***1****.*

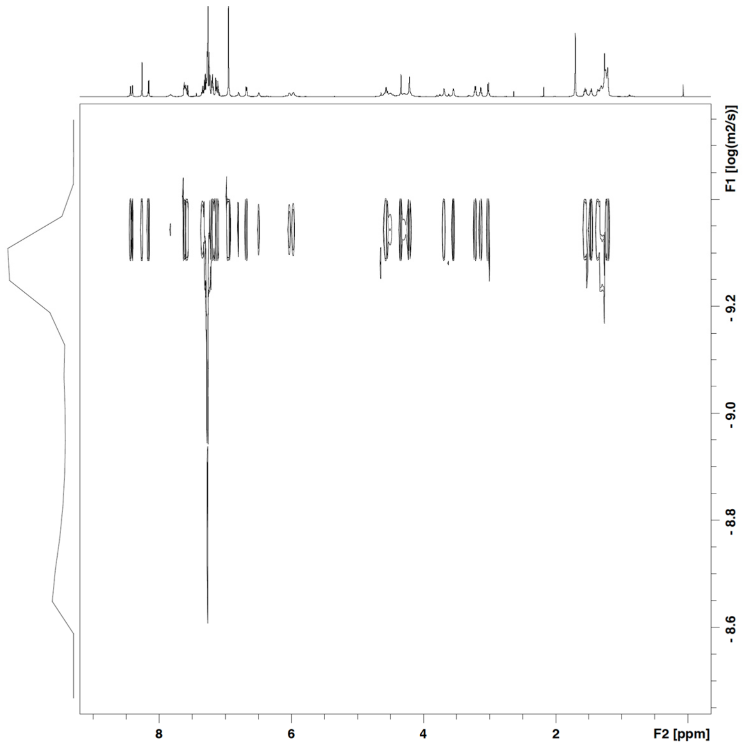

Figure S11. *DOSY 2D (600 MHz, 2 x 10^{-3} M, $CDCl_3$, 298 K) of [2]rotaxane* ***2*** *in $CDCl_3$.*

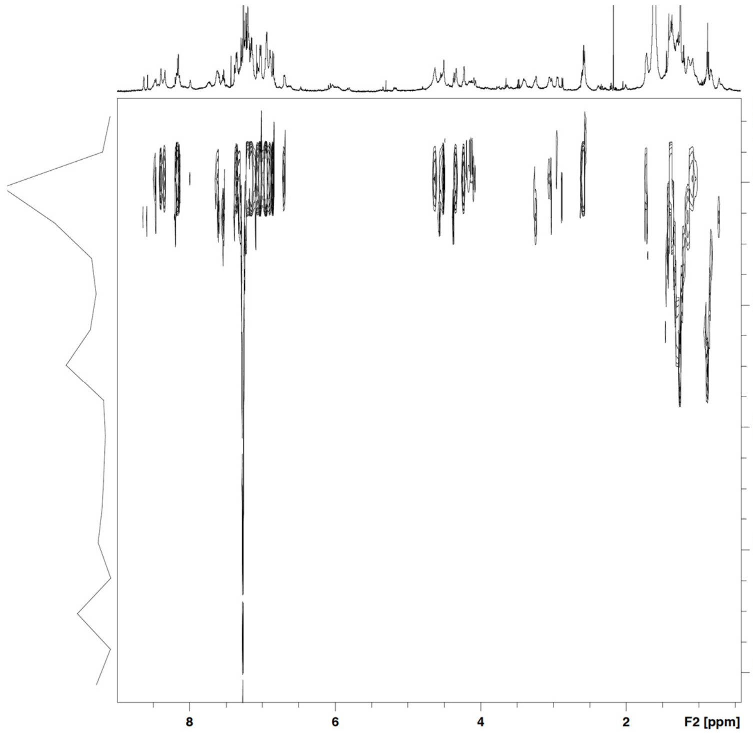

Figure S12. *DOSY 2D (600 MHz, 2 x 10^{-3} M, $CDCl_3$, 298 K) of* ***5****.*

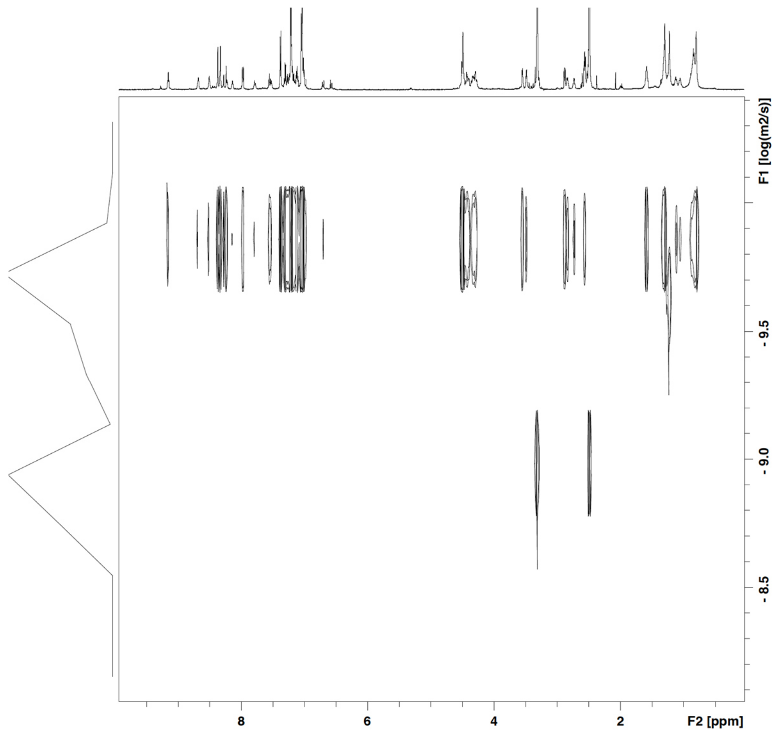

Figure S13. *DOSY 2D (600 MHz, 2 x 10*$^{-3}$ *M, DMSO-d*$_6$*, 298 K) of* ***5****.*

10. MALDI-TOF spectra of compound 5

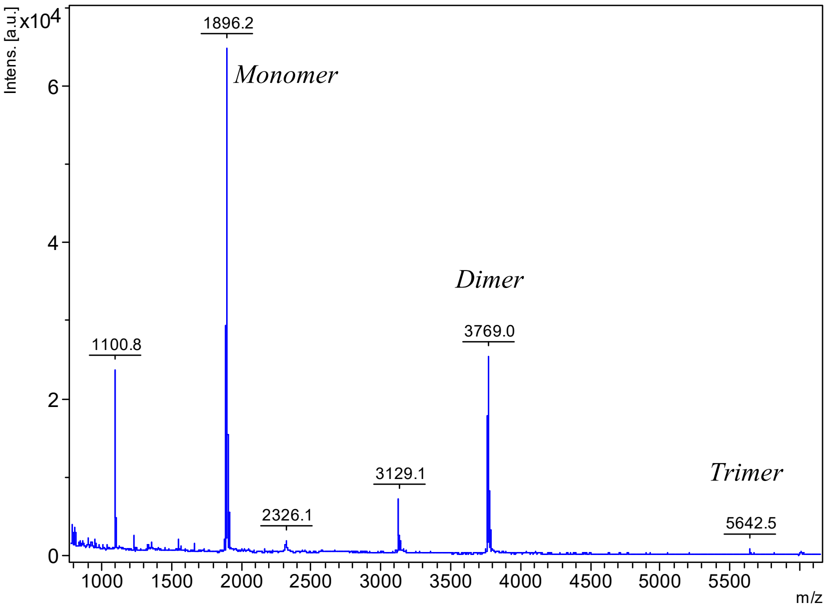

Figure S14. *MALDI-TOF spectrum of compound* ***5****.*

11. Titration experiments

11.1 Titration of macrocycle 3 with thread 1

^{1}H NMR titration spectra were recorded on a Bruker Avance 400 MHz spectrometer, in $CDCl_3$ at 298 K.

<u>Method for the titration with thread **1**</u>: A solution of thread **1** (40 mM, and 2 mM in macrocycle **3**) was added to a solution of macrocycle **3** (0.5 mL, 2 mM). The chemical shift of the host proton NH_C was monitored for 15 titration points (for 0.0-25 equivalents of added guest).

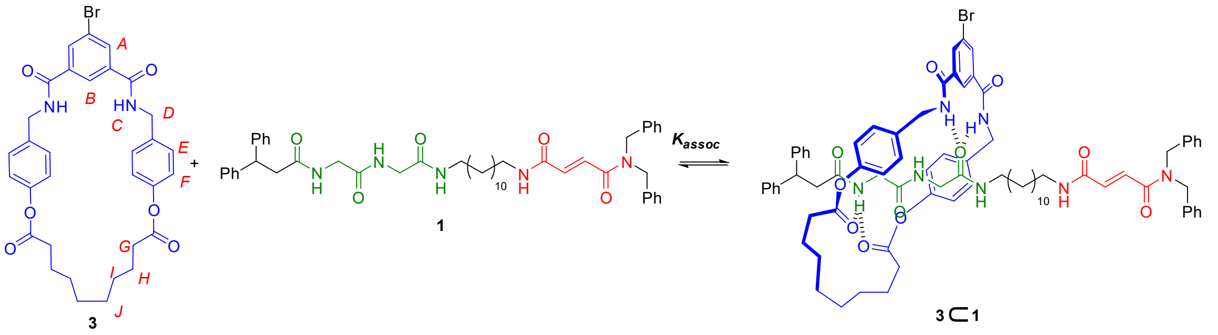

$$\mathbf{3} + \mathbf{1} \overset{k_{assoc}}{\rightleftharpoons} \mathbf{3} \subset \mathbf{1}$$

$$K_{assoc} = 418 \pm 1\ M^{-1}$$

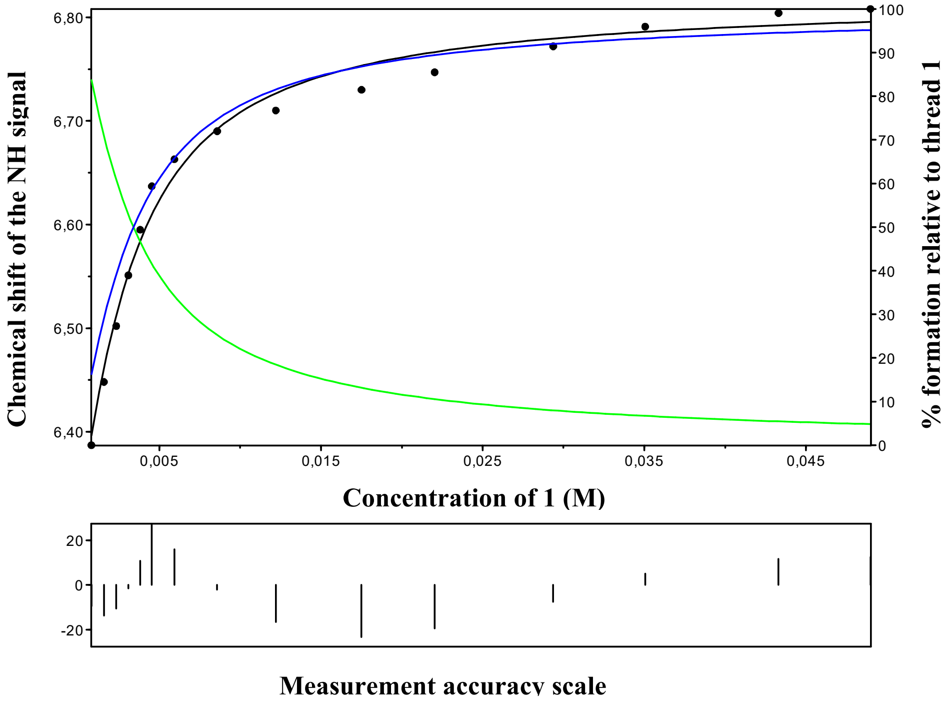

Figure S15. *Data of HypNMR2008 for the association constant of compounds* ***3*** *and* ***1*** *monitored by* ^{1}*H-NMR spectroscopy (400 MHz,* $CDCl_3$*, 298 K).*

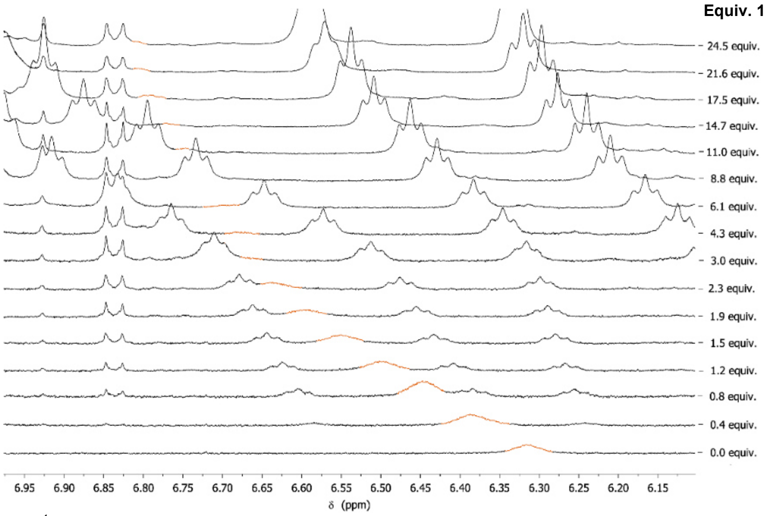

Figure S16. *^{1}H-NMR titration (400 MHz, CDCl$_3$, 298 K) of compounds **3** and **1**.*

11.2 Titration of macrocycle 3 and pyridine *N*-oxide 6

^{1}H NMR titration spectra were recorded on a Bruker Avance 400 MHz spectrometer, in CDCl$_3$ at 298 K.

<u>Method for the titration with pyridine *N*-oxide **6**</u>: A solution of pyridine *N*-oxide **6** (80 mM, and 1 mM in macrocycle **3**) was added to a solution of host (**3**) (0.5 mL, 1 mM). The chemical shift of the host proton NH$_C$ was monitored for 31 titration points (for 0.0-32 equivalents of added guest).

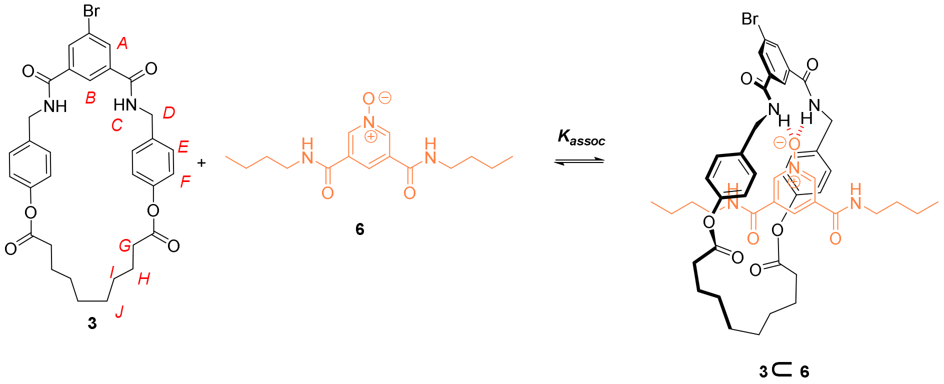

$$\mathbf{3} + \mathbf{6} \xrightleftharpoons{k_{assoc}} \mathbf{5} \subset \mathbf{6}$$

K_a = 529 ± 2 M^{-1}

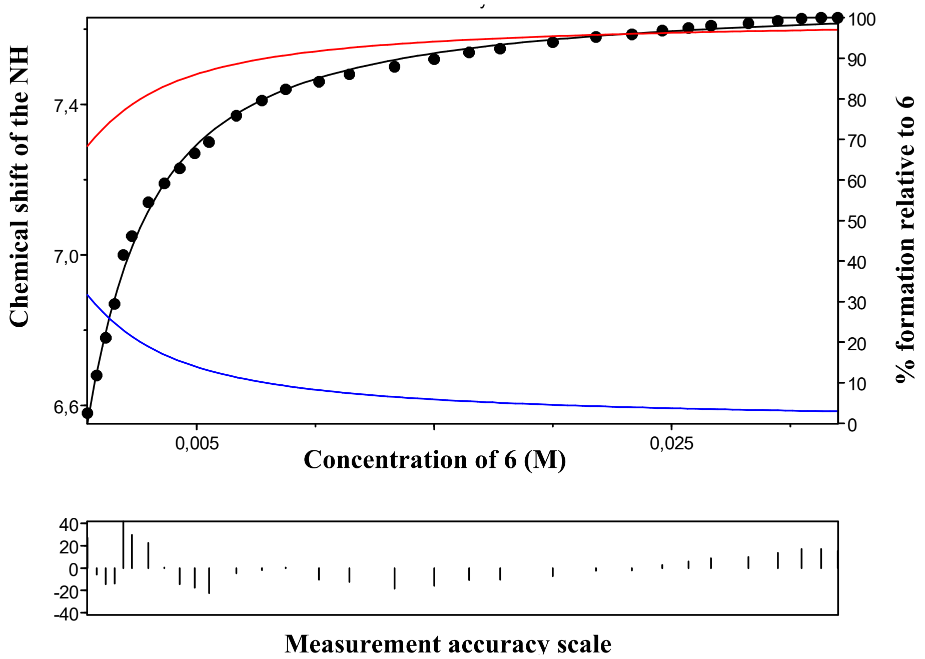

Figure S17. *Data of HypNMR2008 for the association constant of compounds* ***3*** *and* ***6*** *monitored by* ^{1}*H-NMR spectroscopy (400 MHz,* $CDCl_3$*, 298 K).*

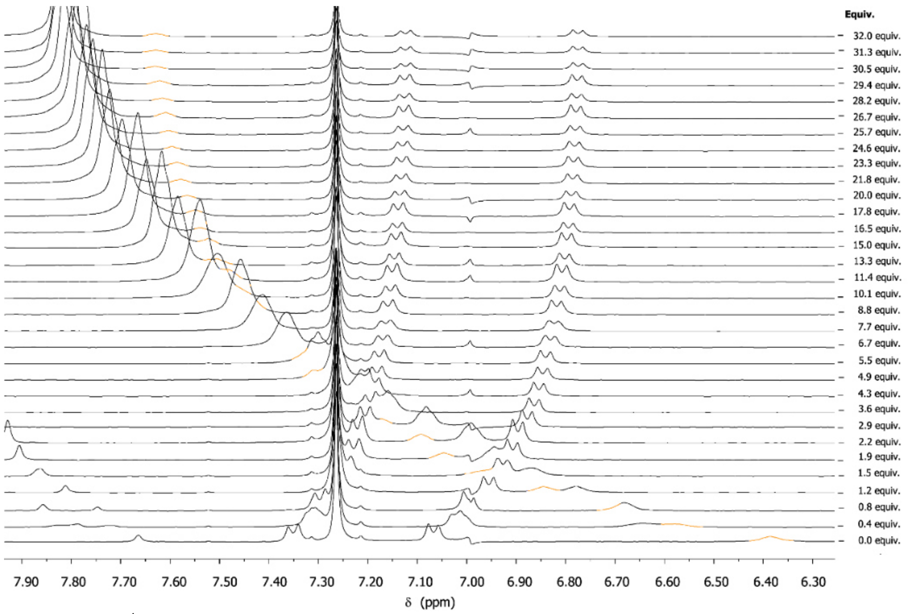

Figure S18. ^{1}*H-NMR titration (400 MHz,* $CDCl_3$*, 298 K) of compounds* ***3*** *and* ***6****.*

12. Disassembly of dimer 5:5 by addition of the pyridine *N*-oxide 6

^{1}H NMR spectra were recorded on a Bruker Avance 400 MHz spectrometer, in $CDCl_3$ at 298 K.

<u>Method</u>: A solution of pyridine *N*-oxide **6** (80 mM) was added to a solution of dimer **5·5** (0.5 mL, 1 mM). Although an association constant could not be determined due to the complexity of the spectra, changes on the signals related to the fumaramide (H_b and H_c) and GlyGly stations (H_r and H_t) were observed. Protons H_b and H_c appeared as two doublets when the guest was added, staying at similar chemical shifts during the experiment. More importantly, the addition of *N*-oxide **6** triggers a shifting of the GlyGly protons H_r and H_t to lower field, an indication that the GlyGly station is decreasing its interaction with the diamine macrocycle. Thus, the disassembly of the hetero[4]pseudorotaxane dimer **5·5** is occurring while the *N*-oxide **6** is added.

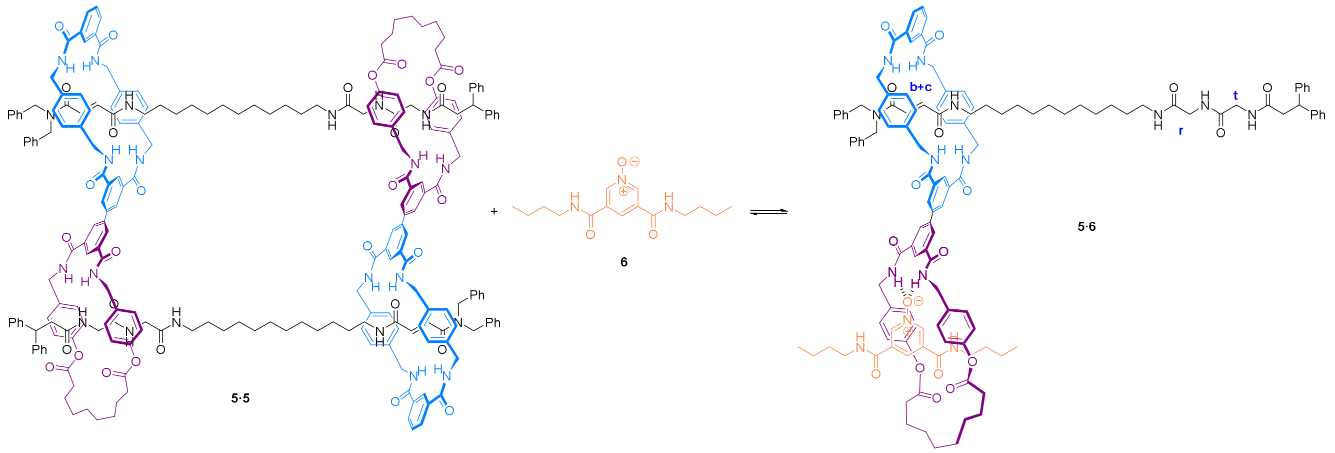

13. ^{1}H and ^{13}C NMR spectra of synthesized compounds.

^{1}H-NMR of S1 (300 MHz, $CDCl_3$, 298 K)

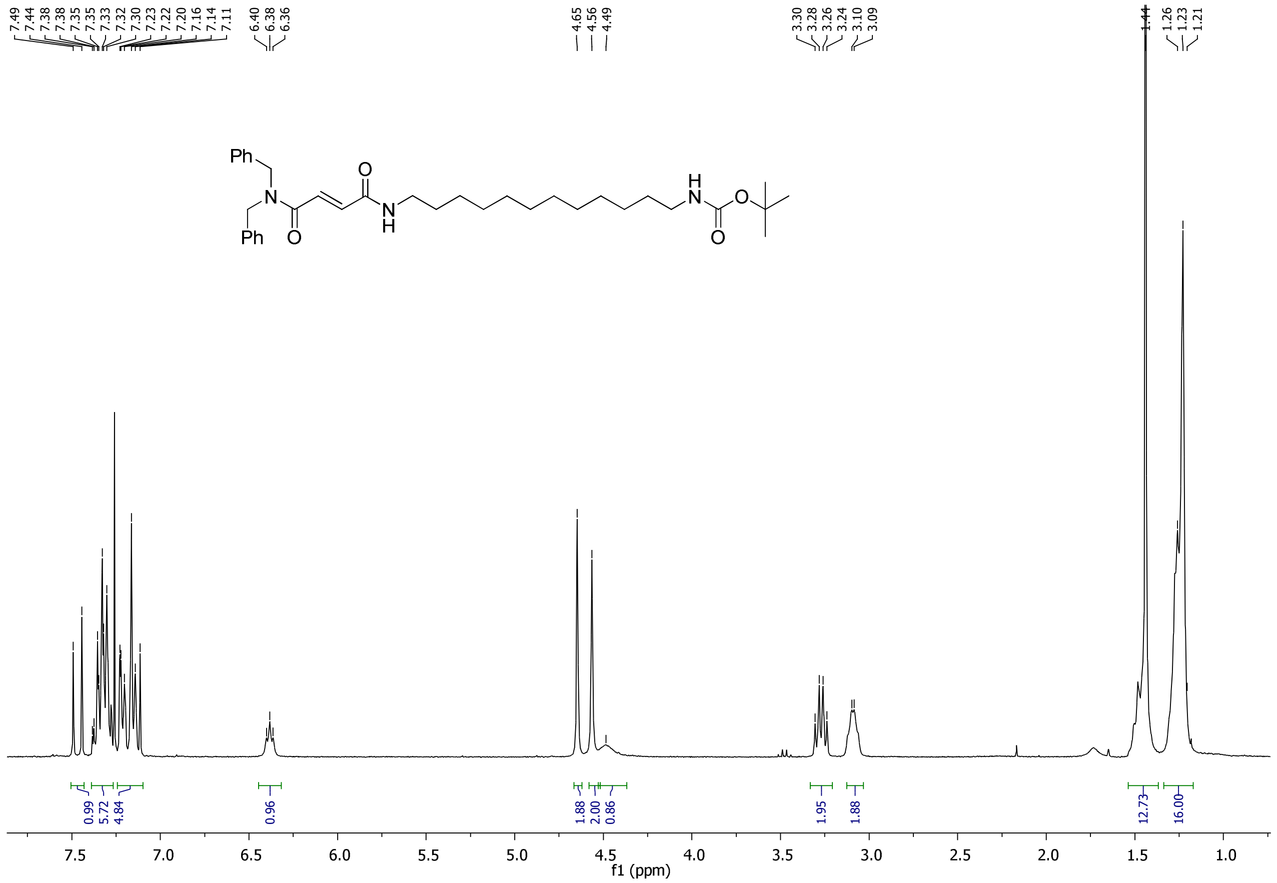

^{13}C-NMR of S1 (75 MHz, $CDCl_3$, 298 K)

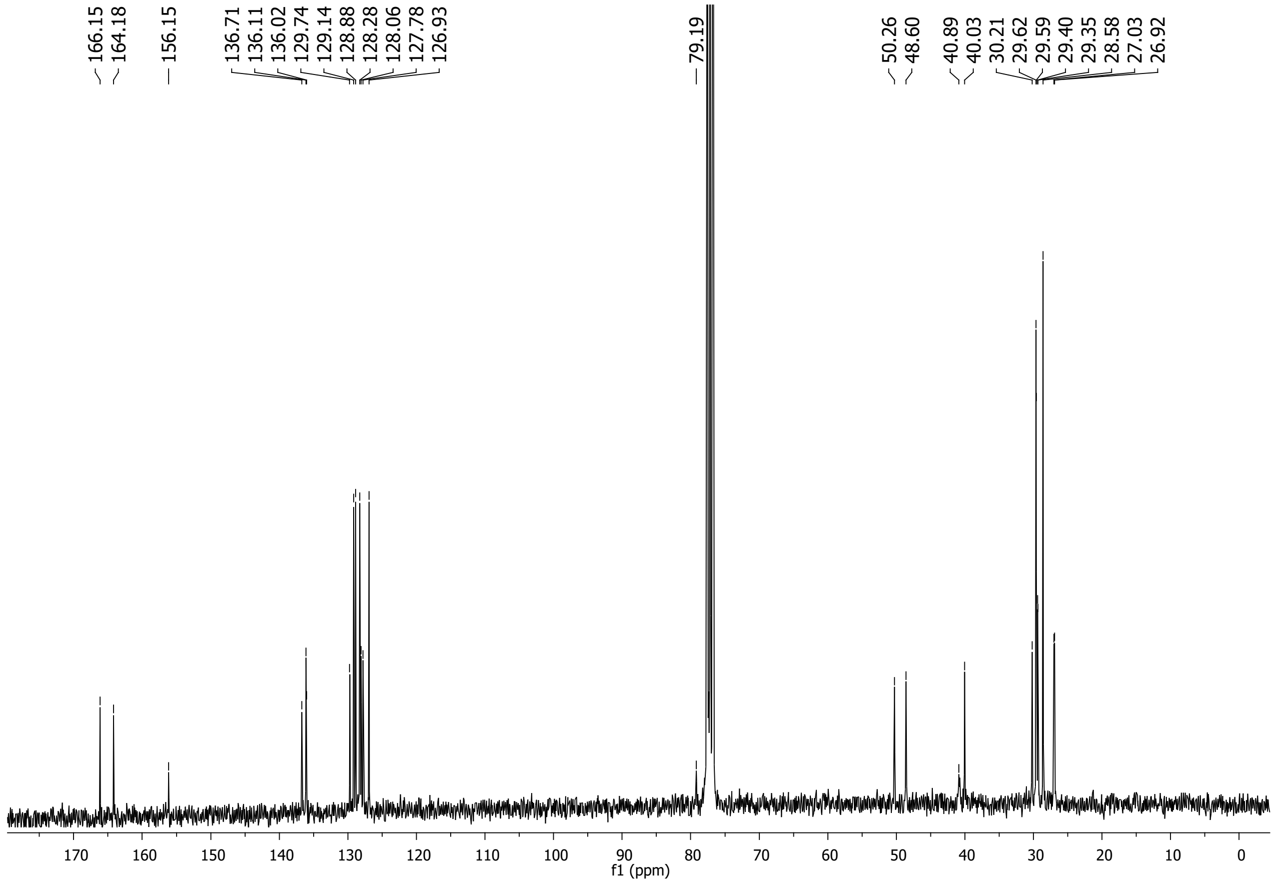

^{1}H-NMR of S2 (400 MHz, DMSO-d_6, 298 K)

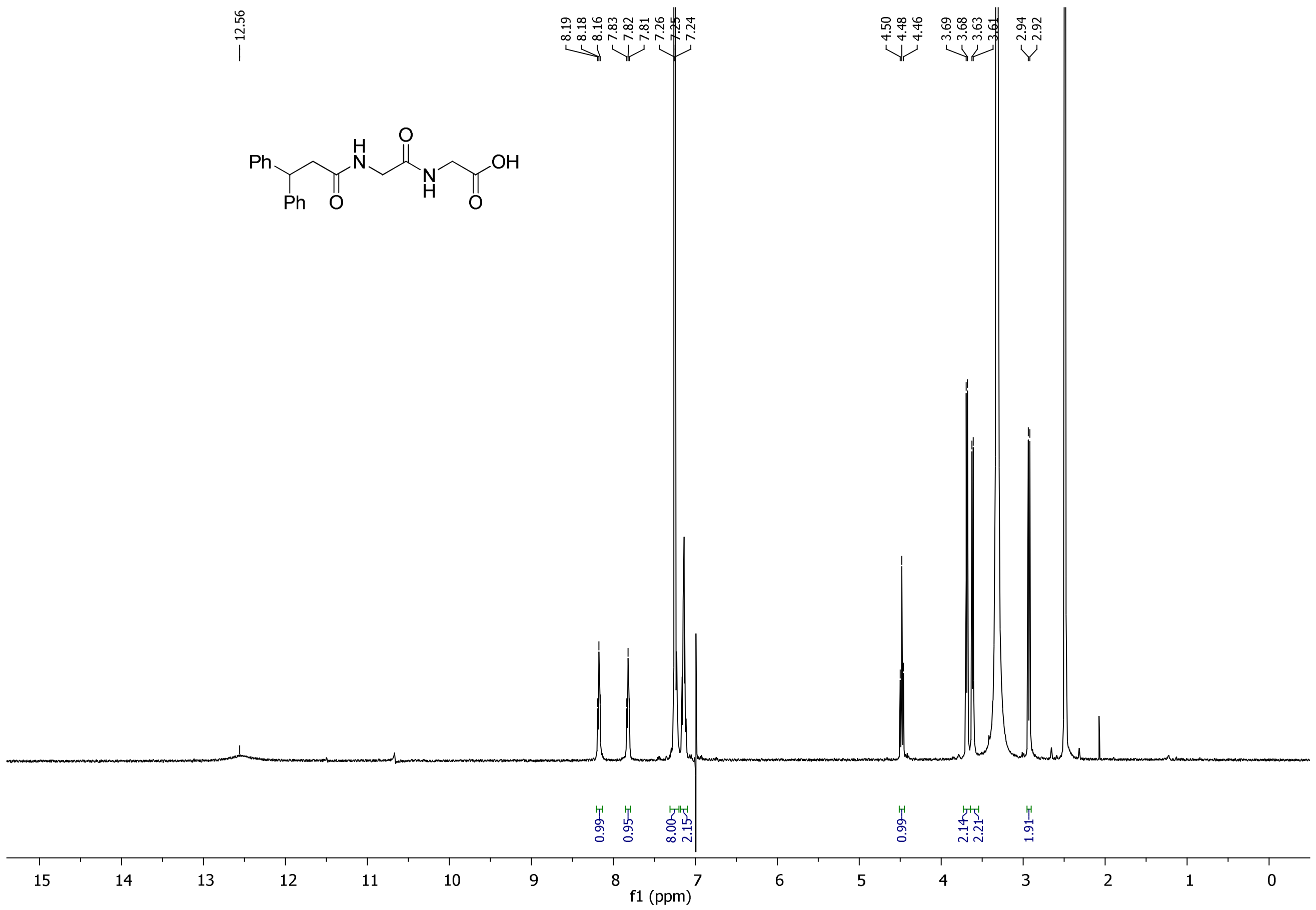

^{13}C-NMR of S2 (100 MHz, DMSO-d_6, 298 K)

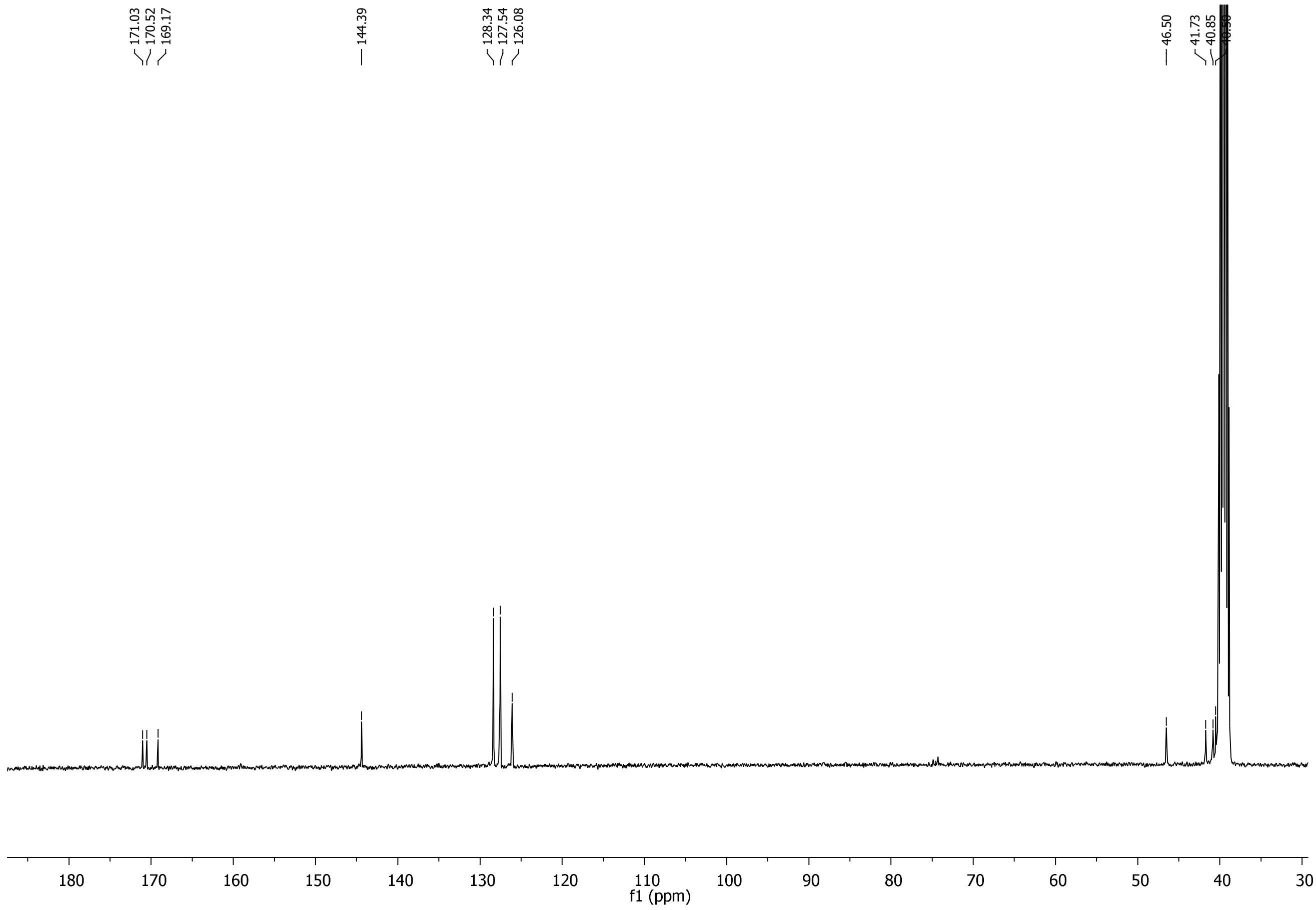

^{1}H-NMR of 1 (400 MHz, $CDCl_3$, 298 K)

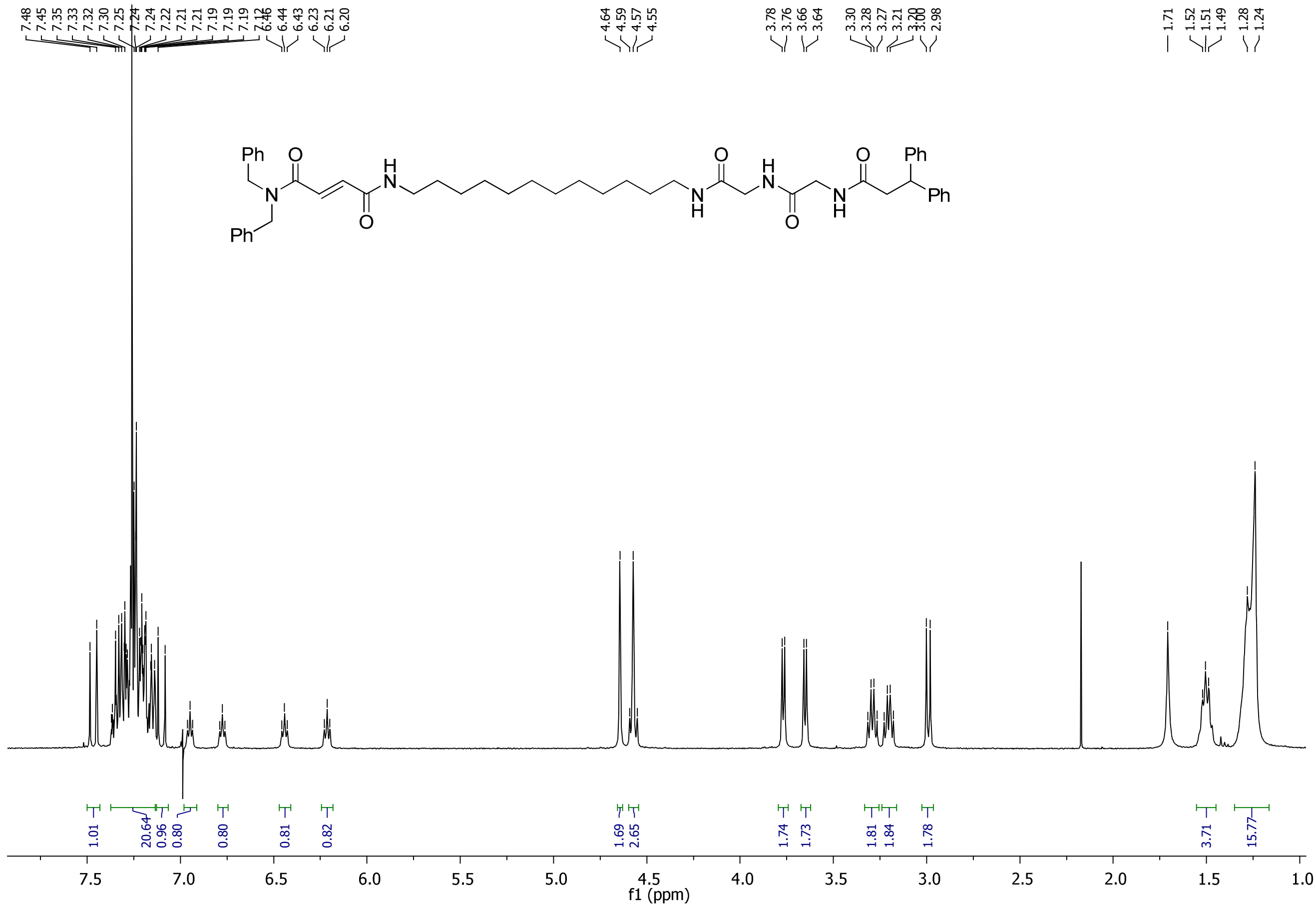

^{13}C-NMR of 1 (100 MHz, $CDCl_3$, 298 K)

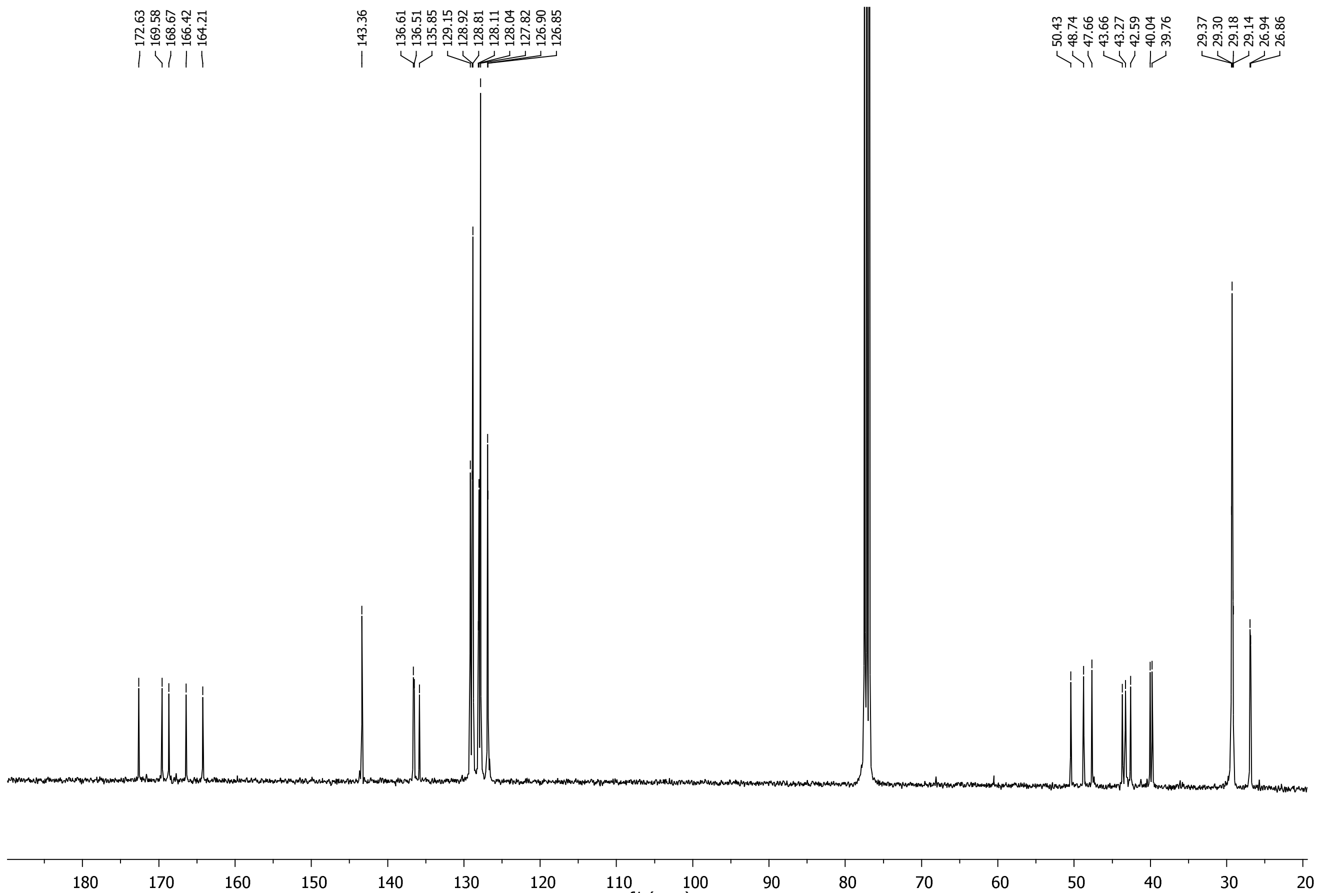

[135]DEPT-NMR of 1 ($CDCl_3$, 298 K)

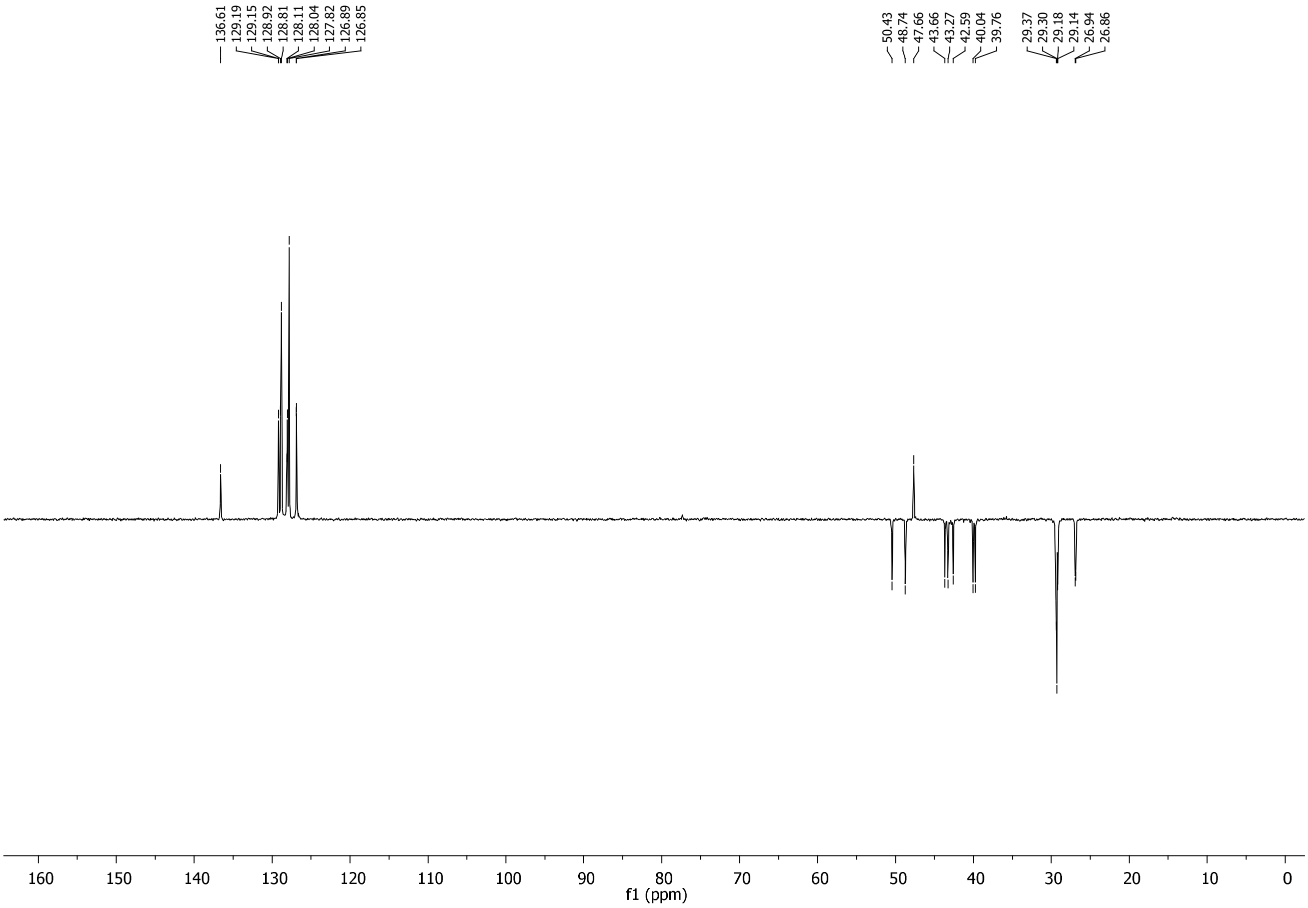

^{1}H-NMR of S3 (400 MHz, DMSO-d_6, 298 K)

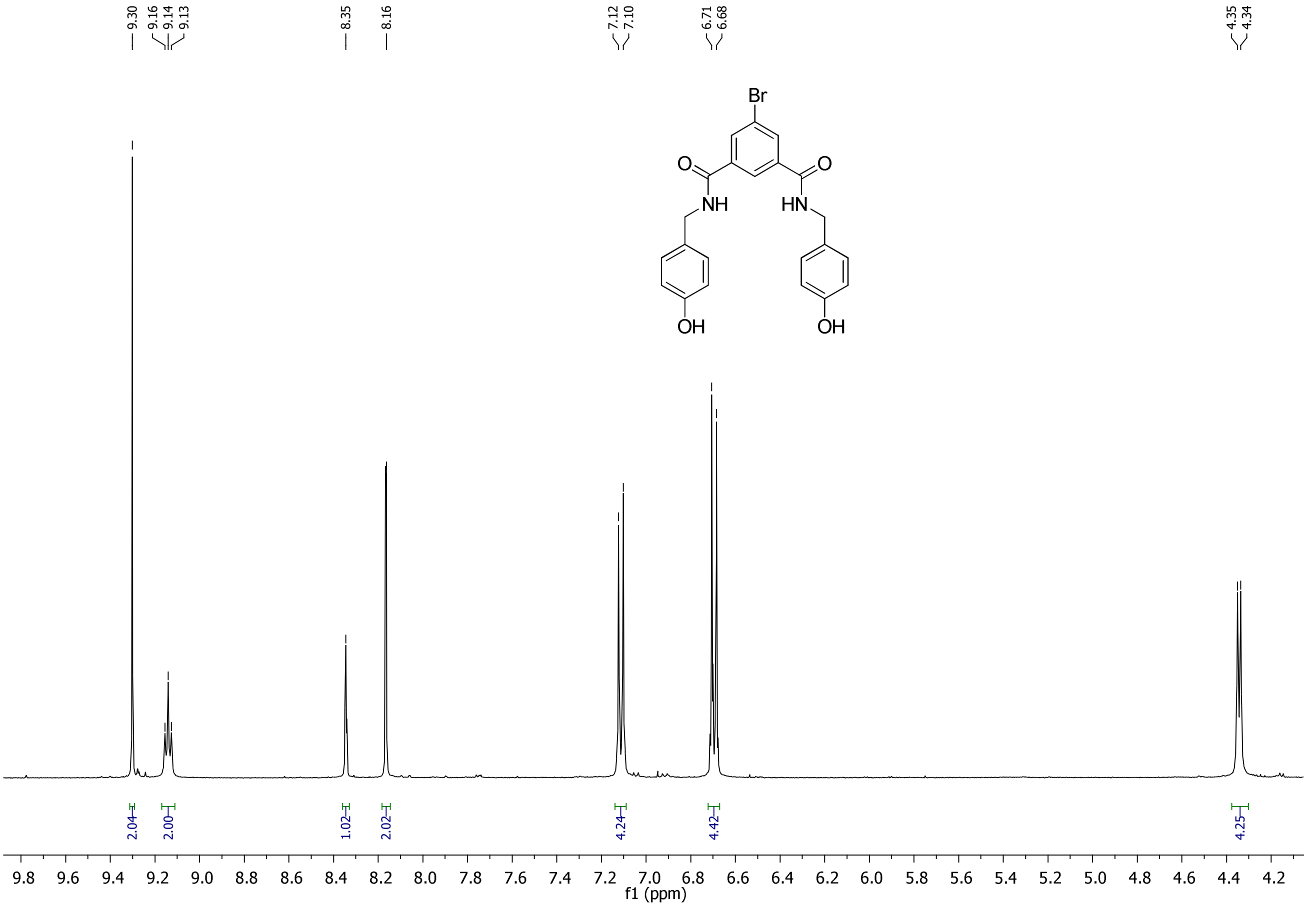

^{13}C-NMR of S3 (100 MHz, DMSO-d_6, 298 K)

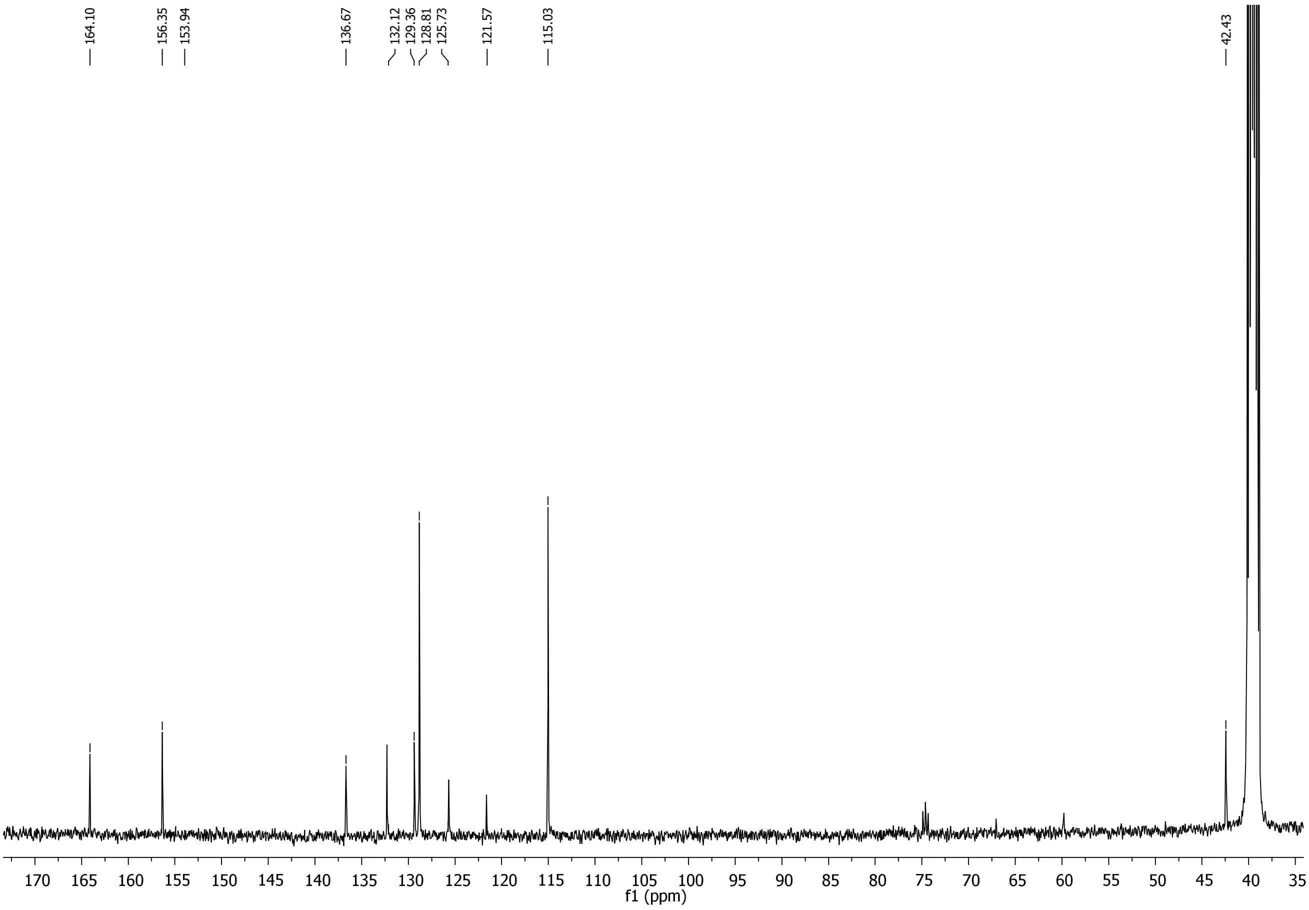

^{1}H-NMR of 3 (400 MHz, $CDCl_3$, 298 K)

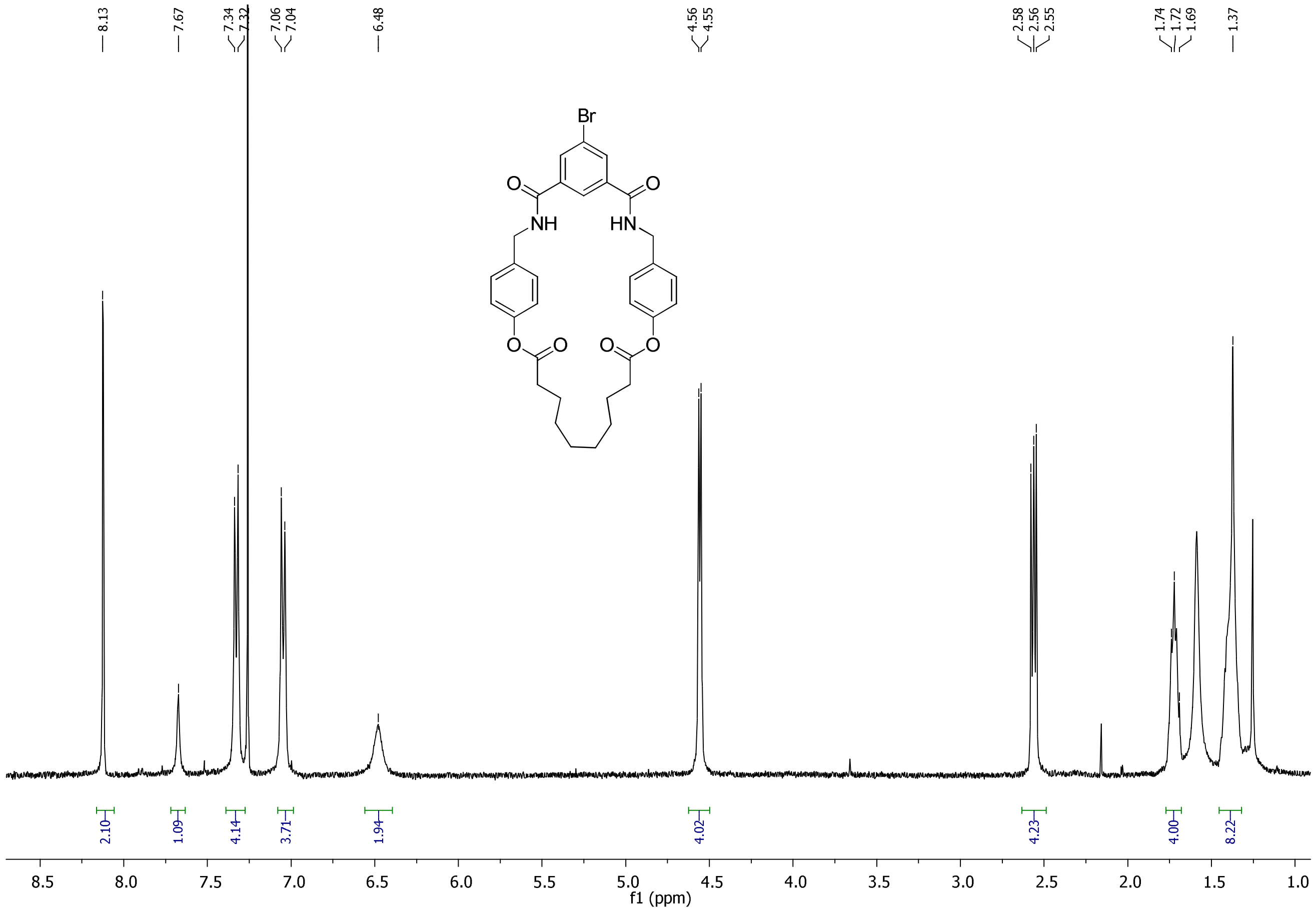

^{13}C-NMR of 3 (100 MHz, $CDCl_3$, 298 K)

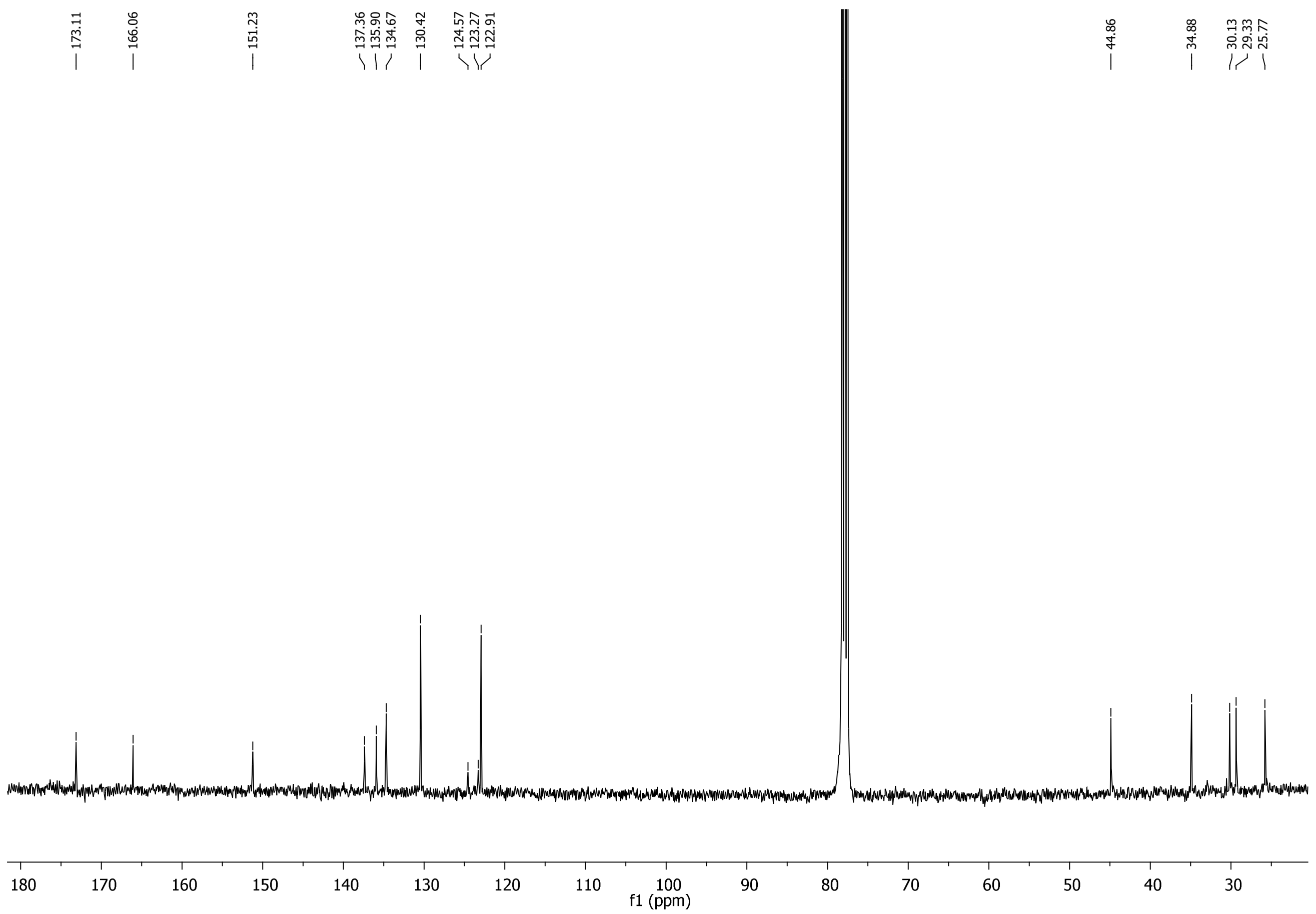

^{1}H-NMR of 4 (300 MHz, $CDCl_3$, 298 K)

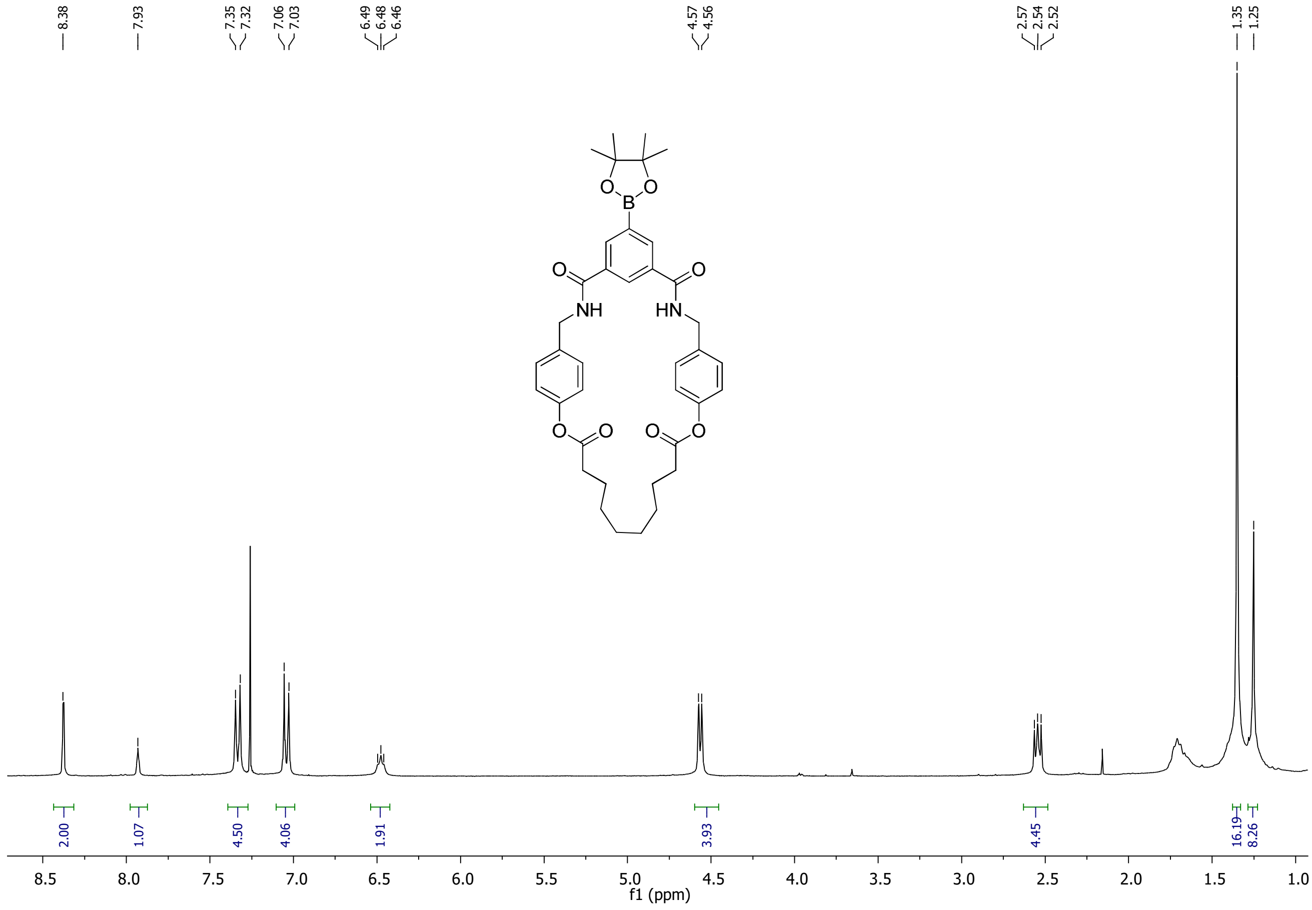

^{13}C-NMR of 4 (75 MHz, $CDCl_3$, 298 K)

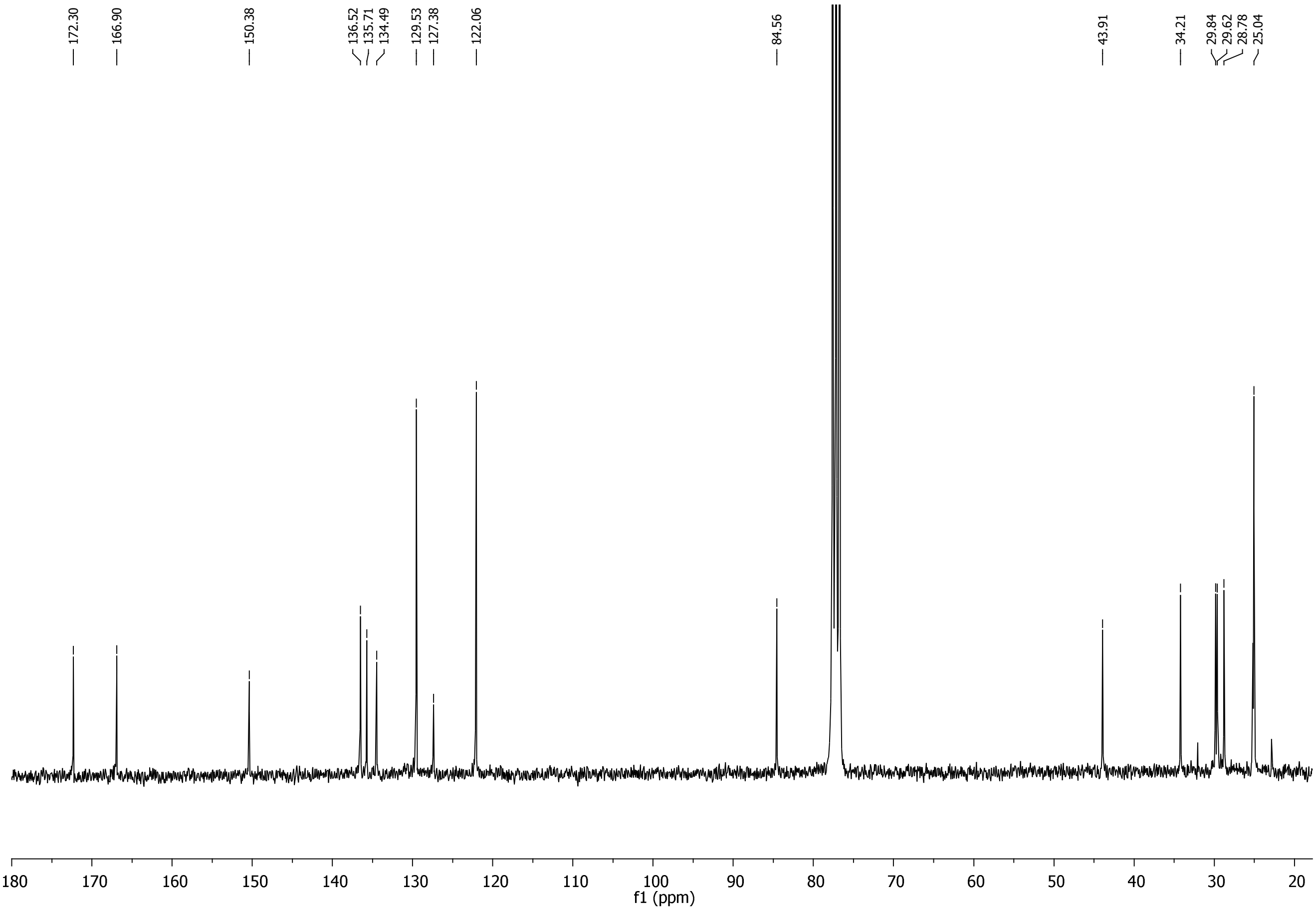

^{1}H-NMR of 2 (400 MHz, $CDCl_3$, 298 K)

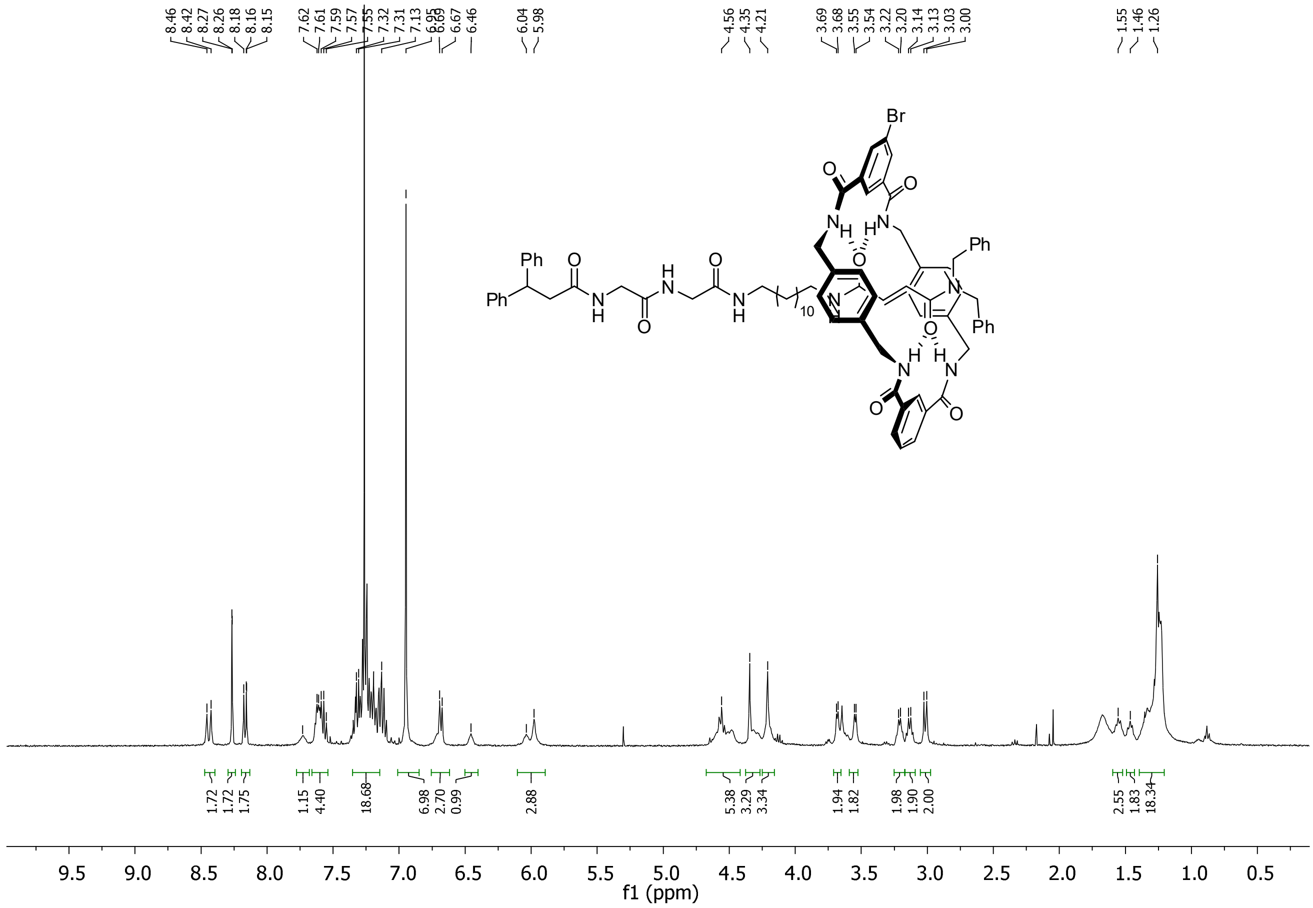

^{13}C-NMR of 2 (100 MHz, $CDCl_3$, 298 K)

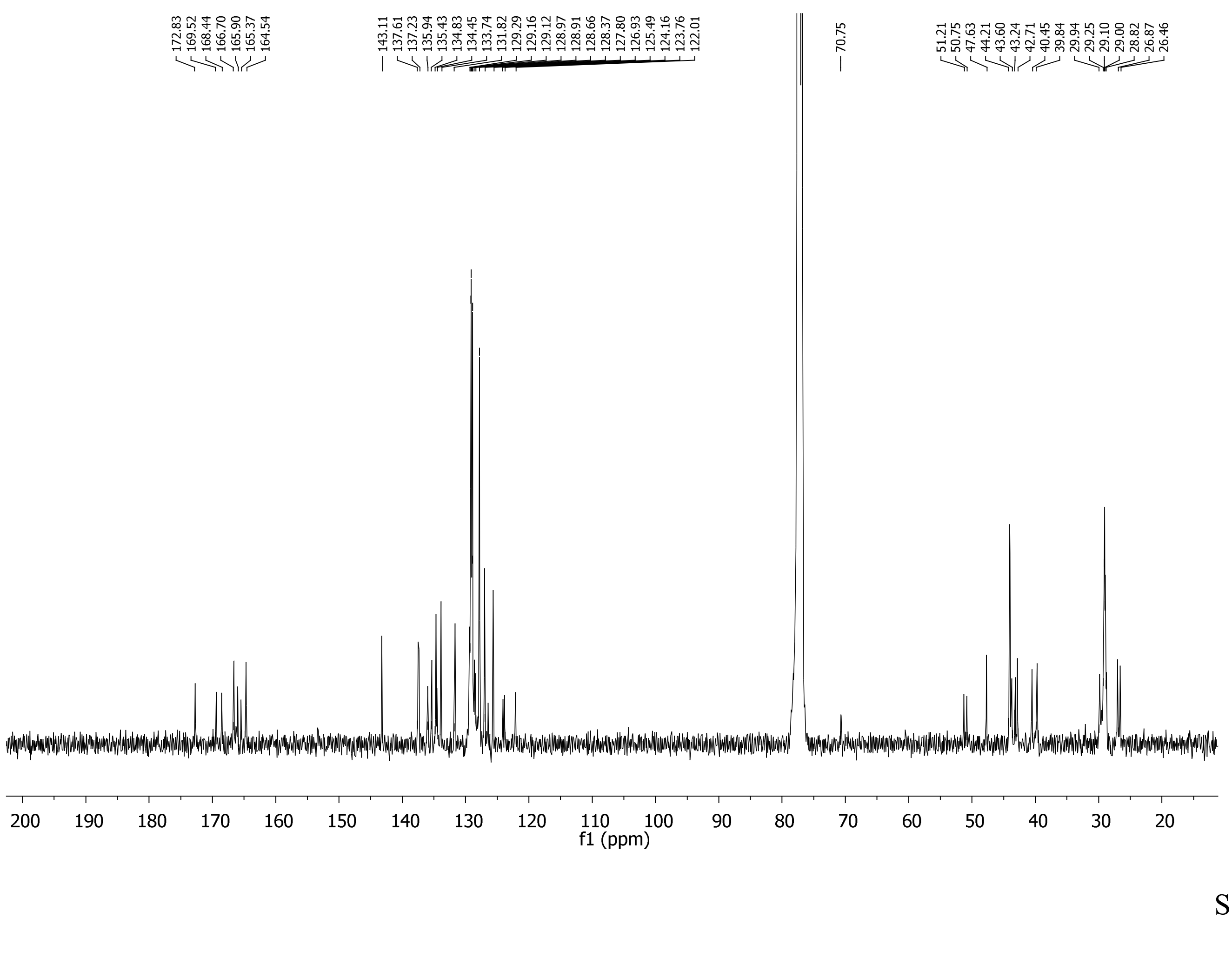

^{135}DEPT-NMR of 2 (100 MHz, $CDCl_3$, 298 K)

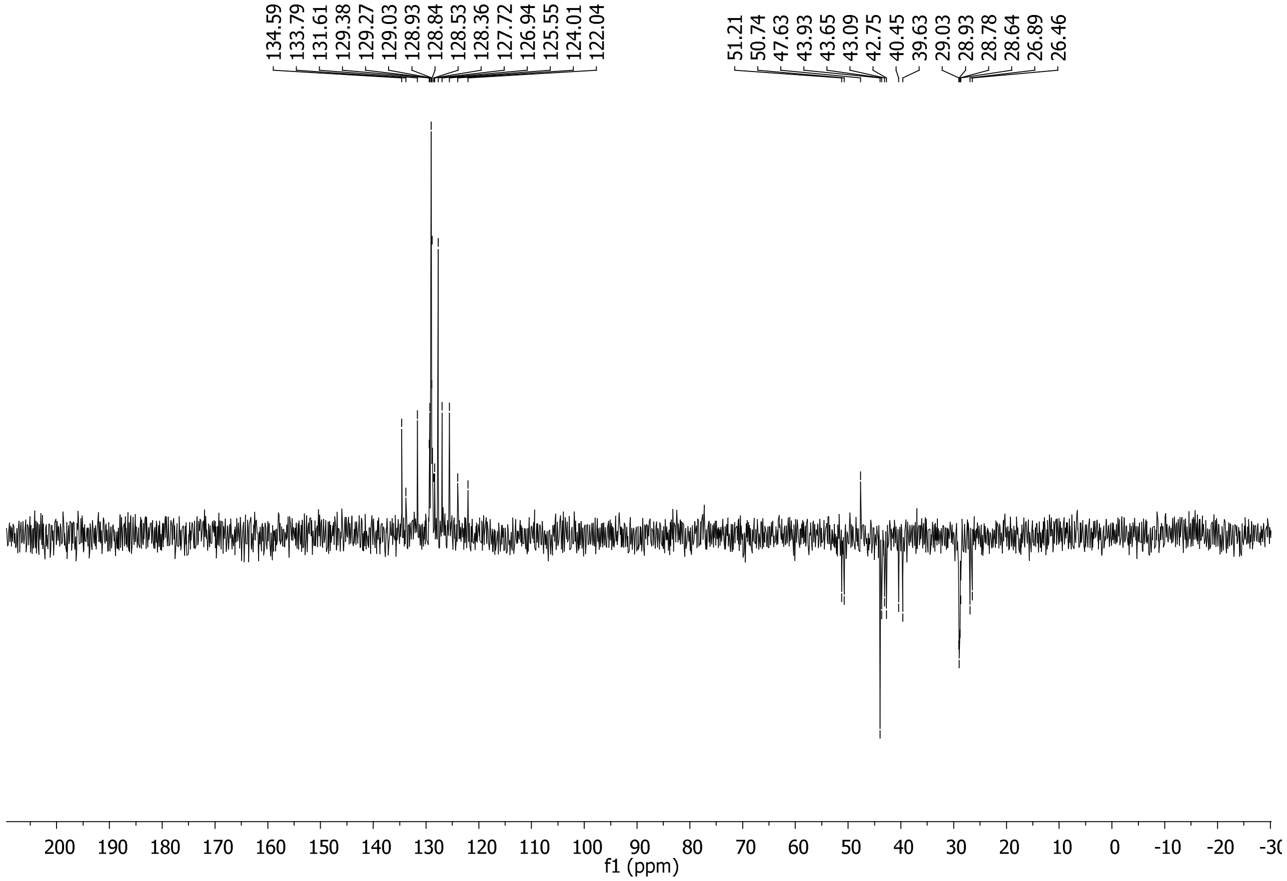

COSY-NMR of 2 (400 MHz, $CDCl_3$, 298 K)

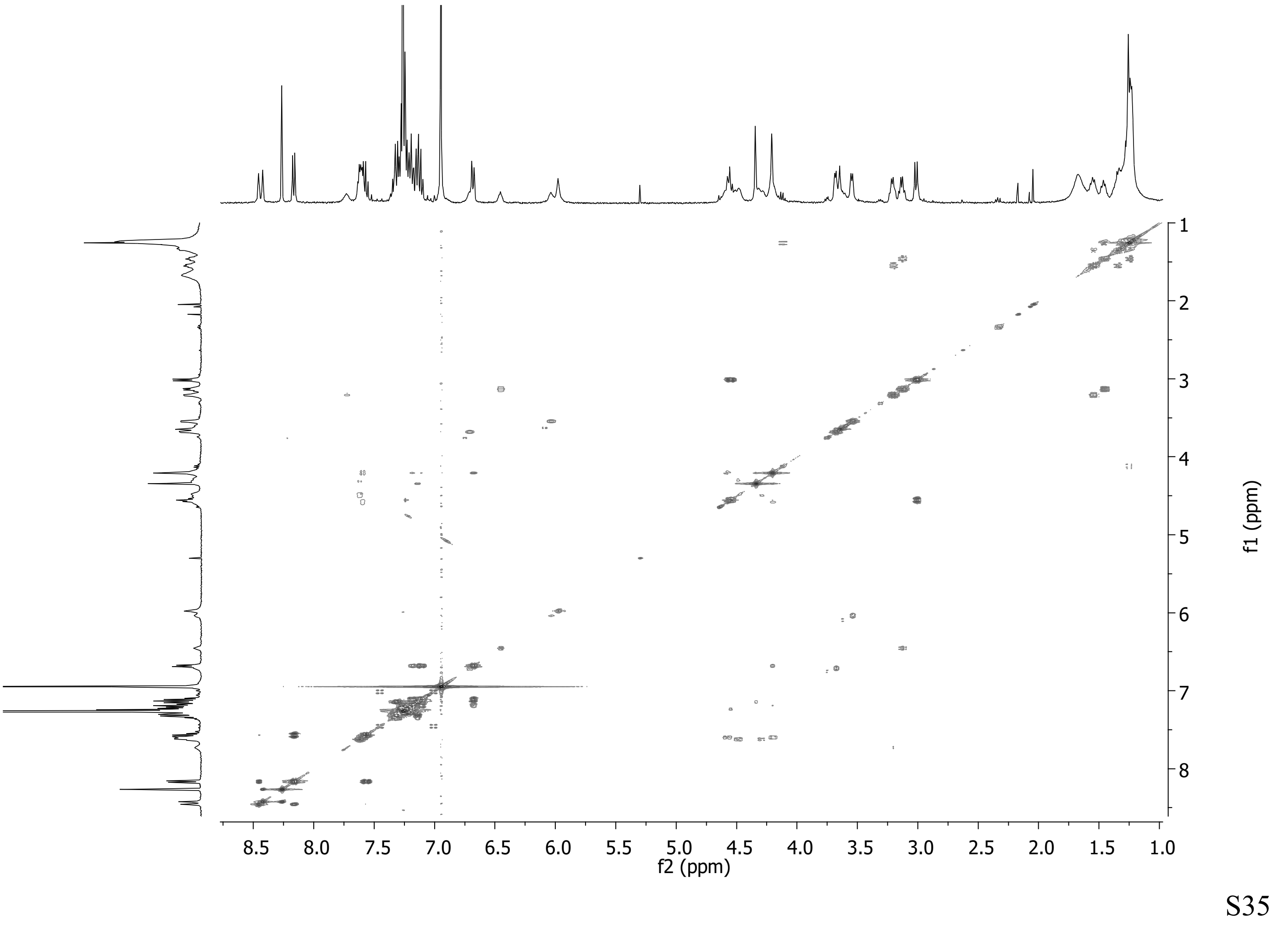

HMQC-NMR of 2 ($CDCl_3$, 298 K)

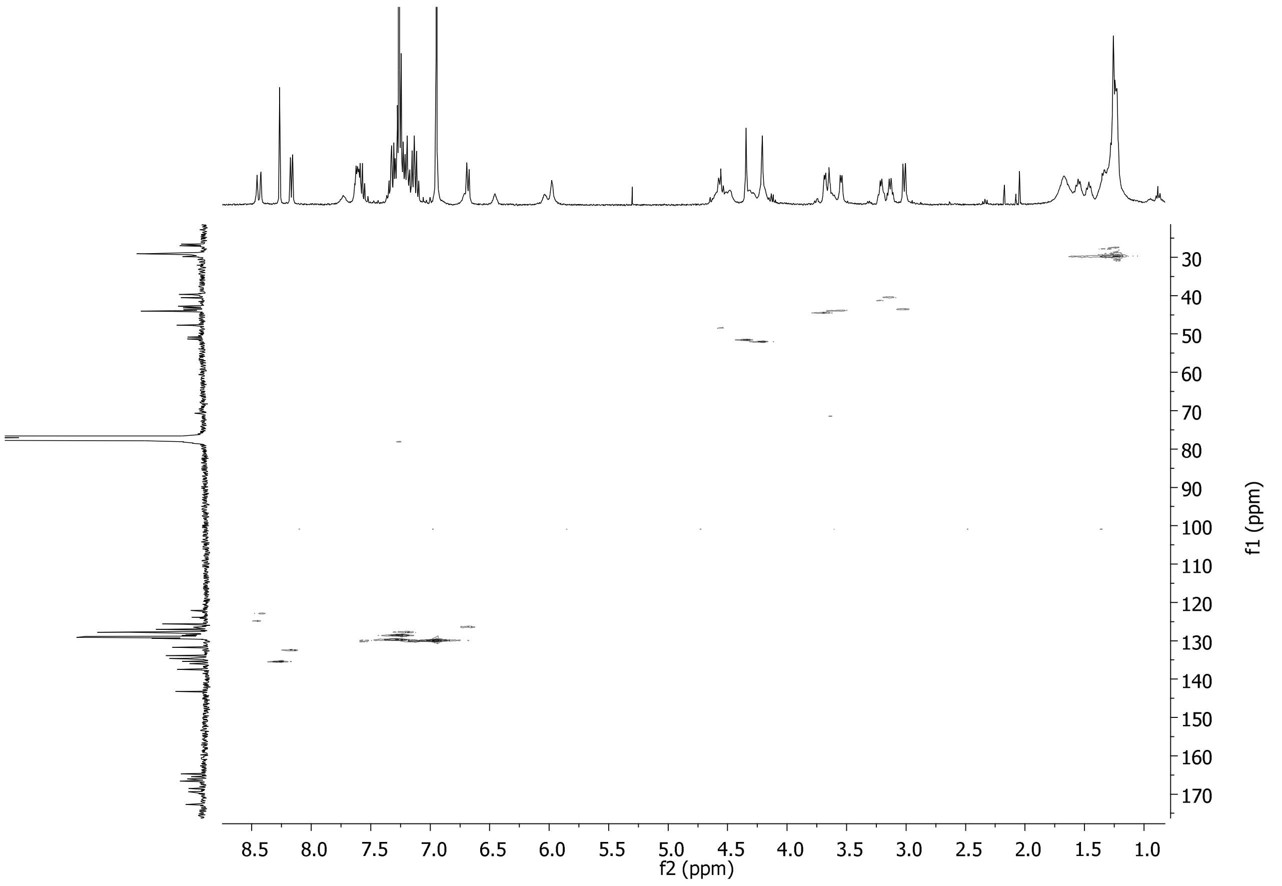

^{1}H-NMR of S4 (400 MHz, $CDCl_3$, 298 K)

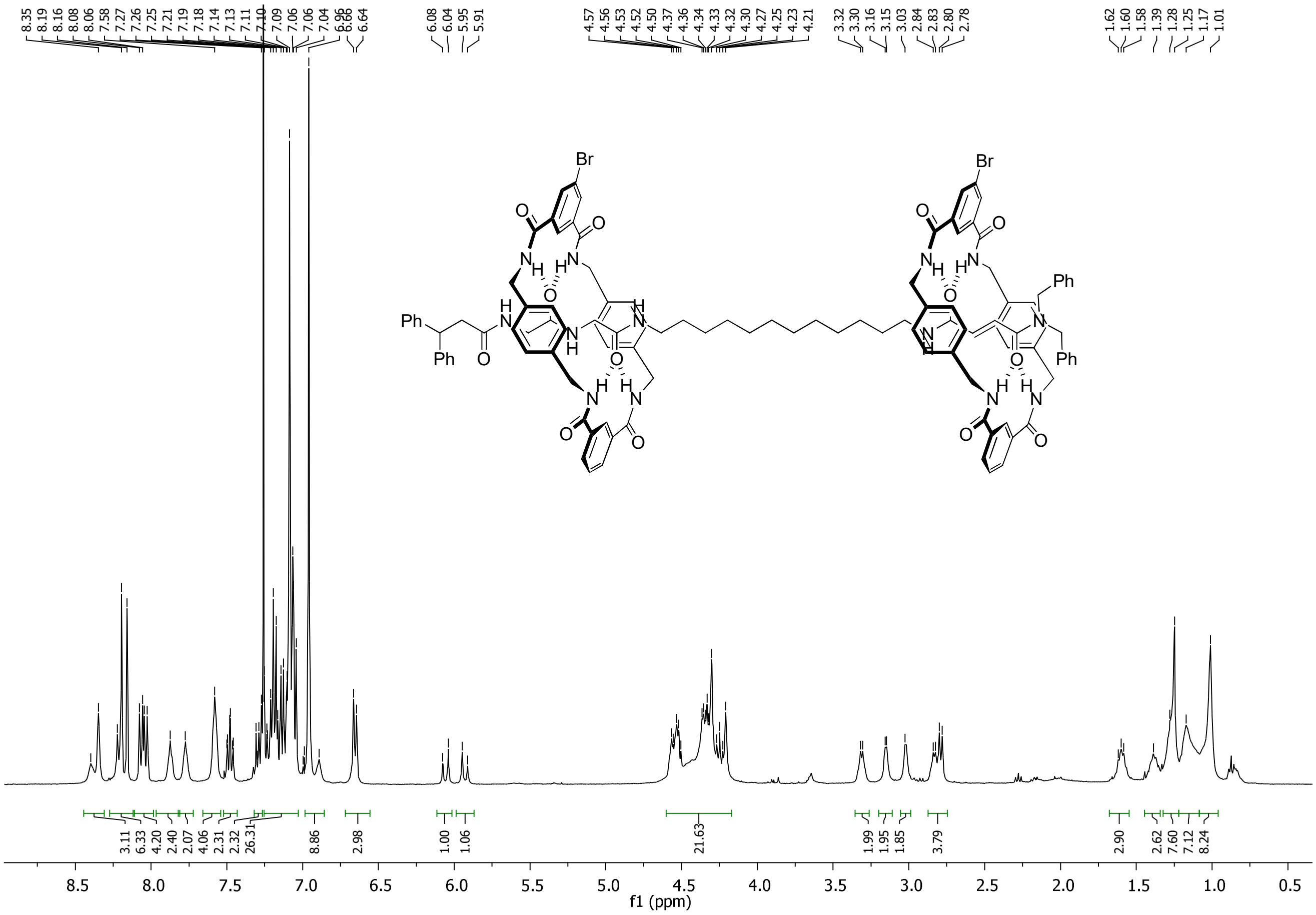

^{13}C-NMR of S4 (100 MHz, $CDCl_3$, 298 K)

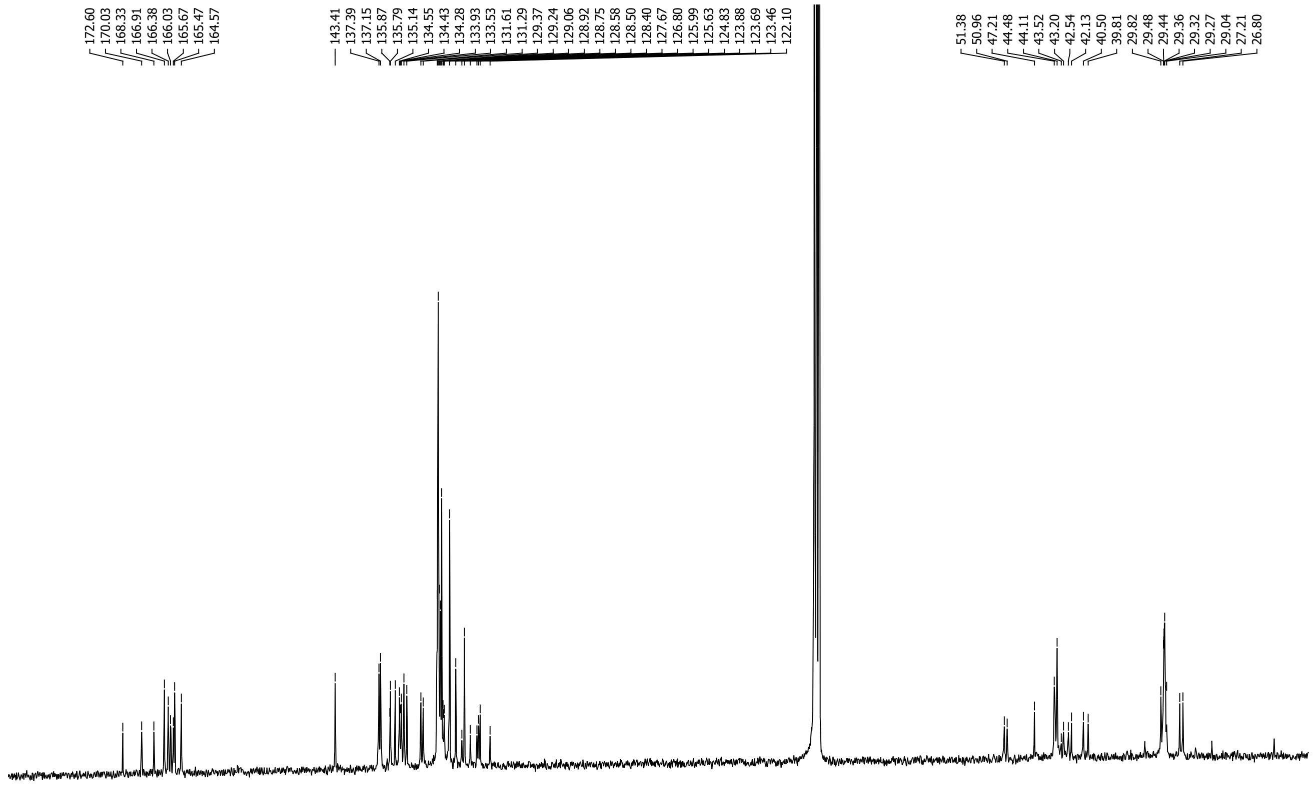

135DEPT-NMR of S4 ($CDCl_3$, 298 K)

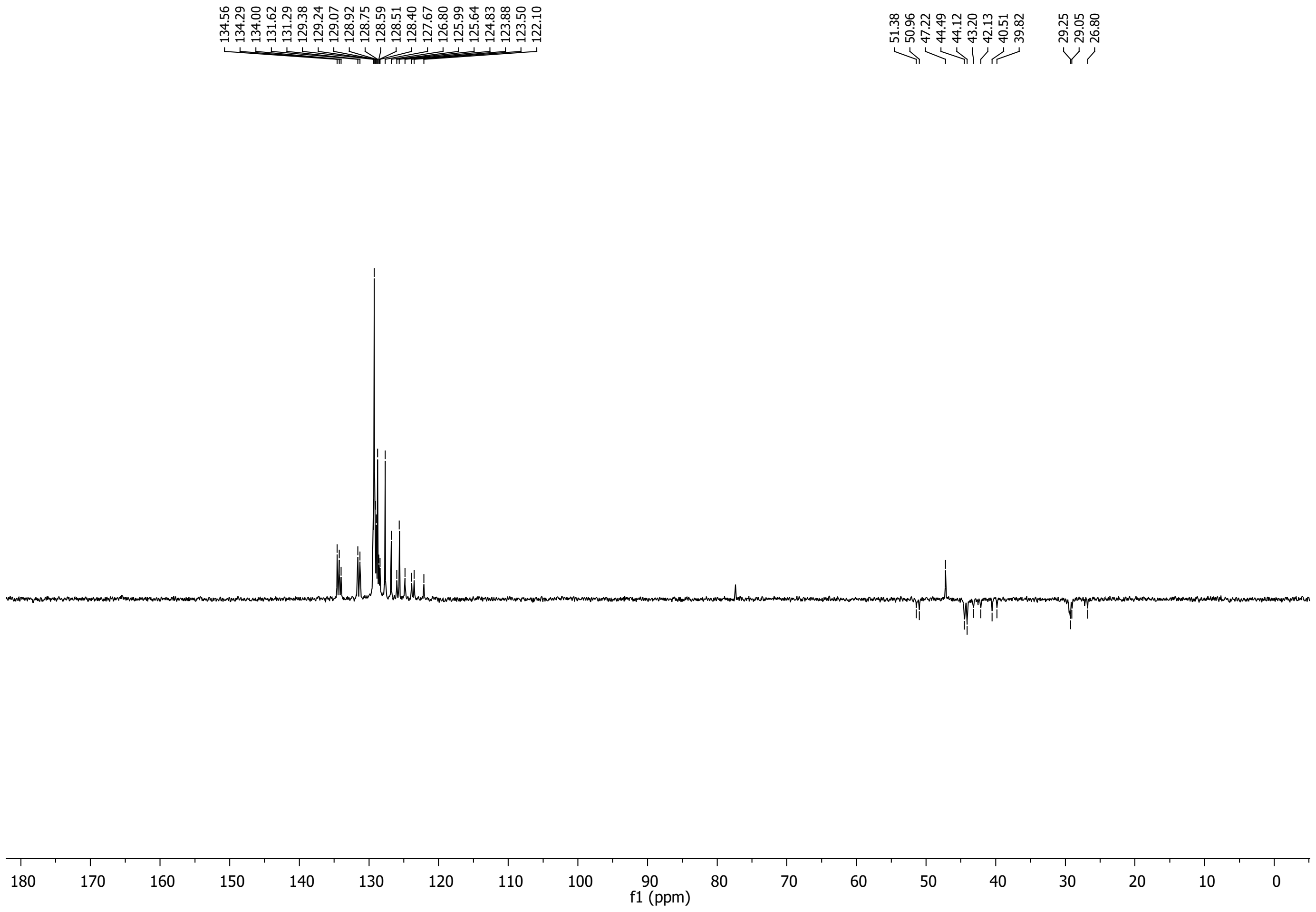

^{1}H-NMR of 5 (400 MHz, DMSO-d_6, 298 K)

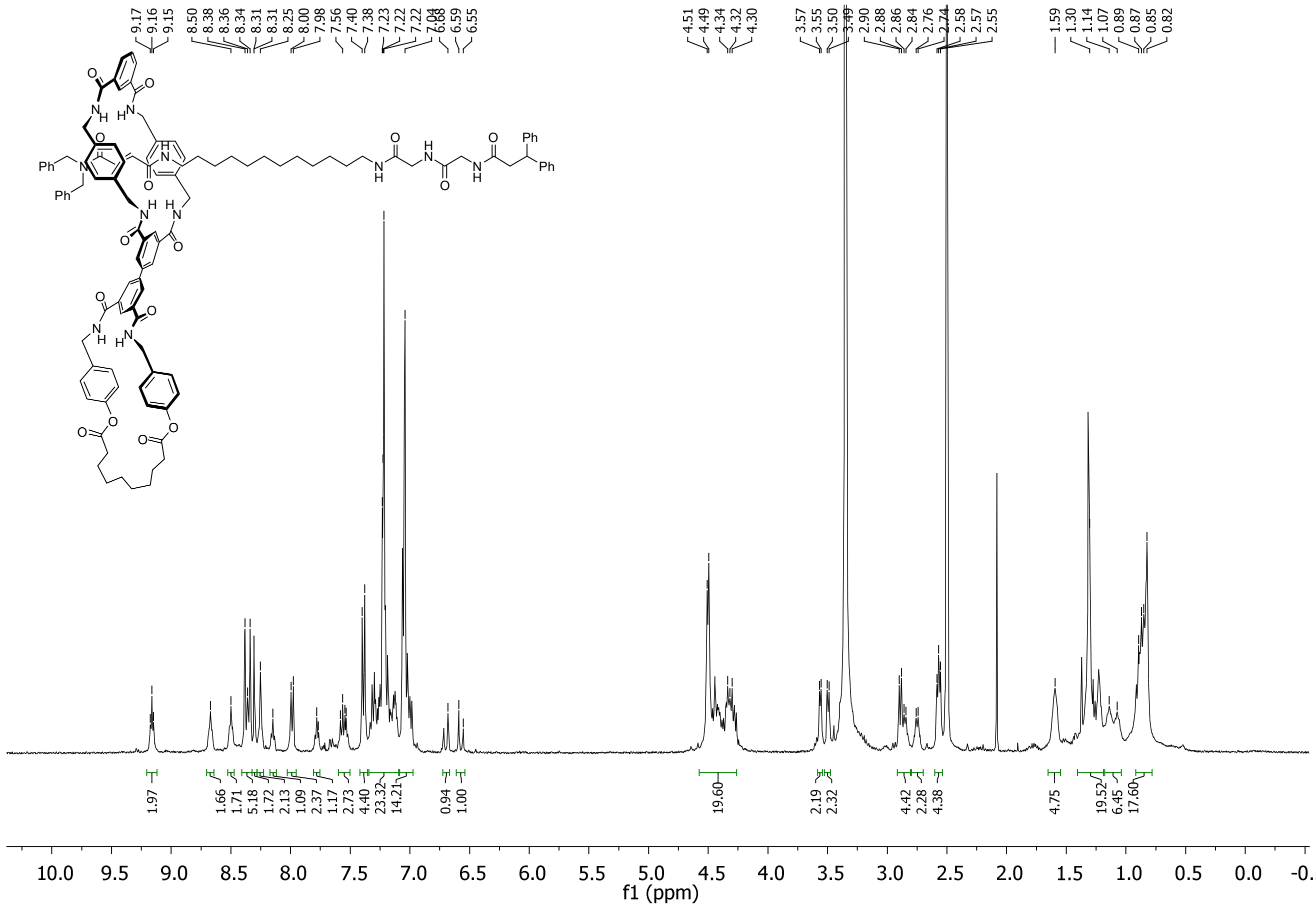

^{13}C-NMR of 5 (100 MHz, DMSO-d_6, 298 K)

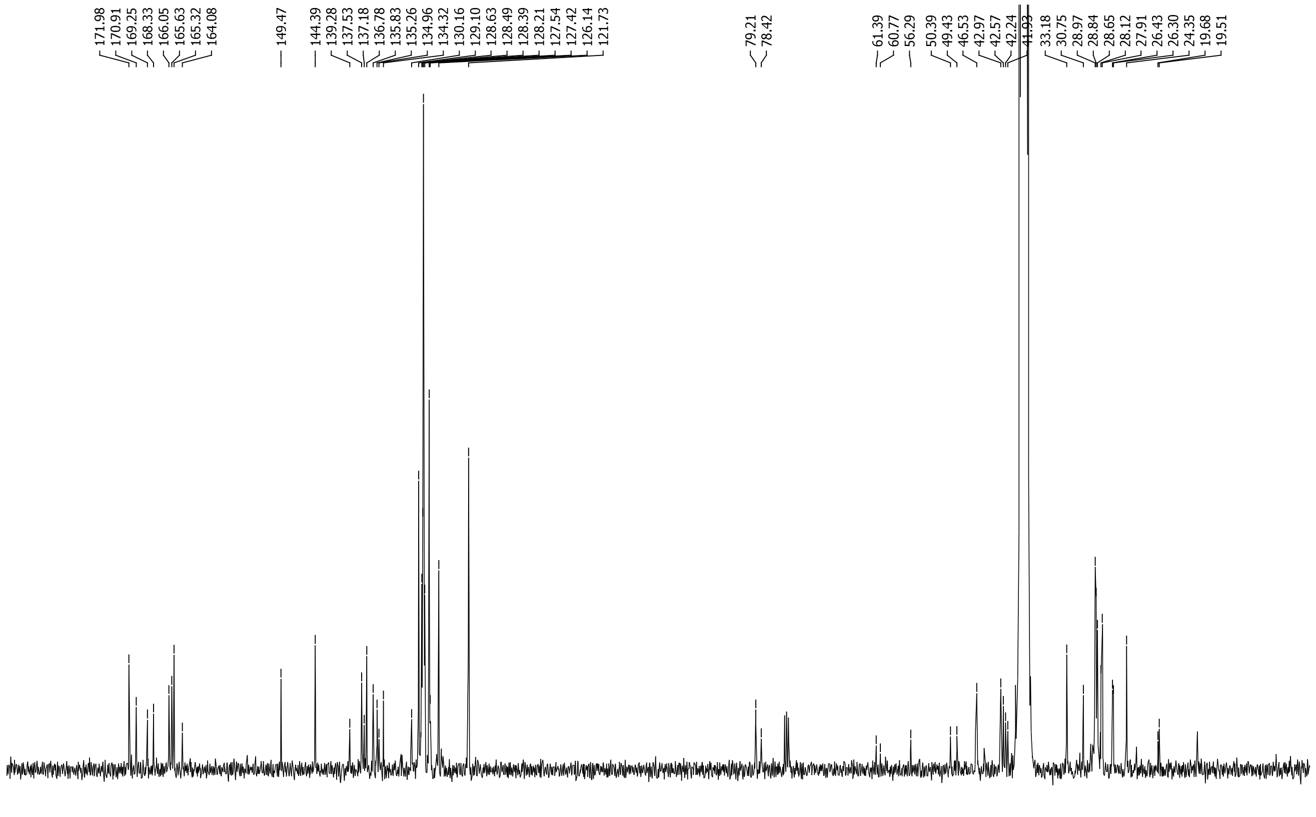

COSY-NMR of 5 (DMSO-d_6, 298 K)

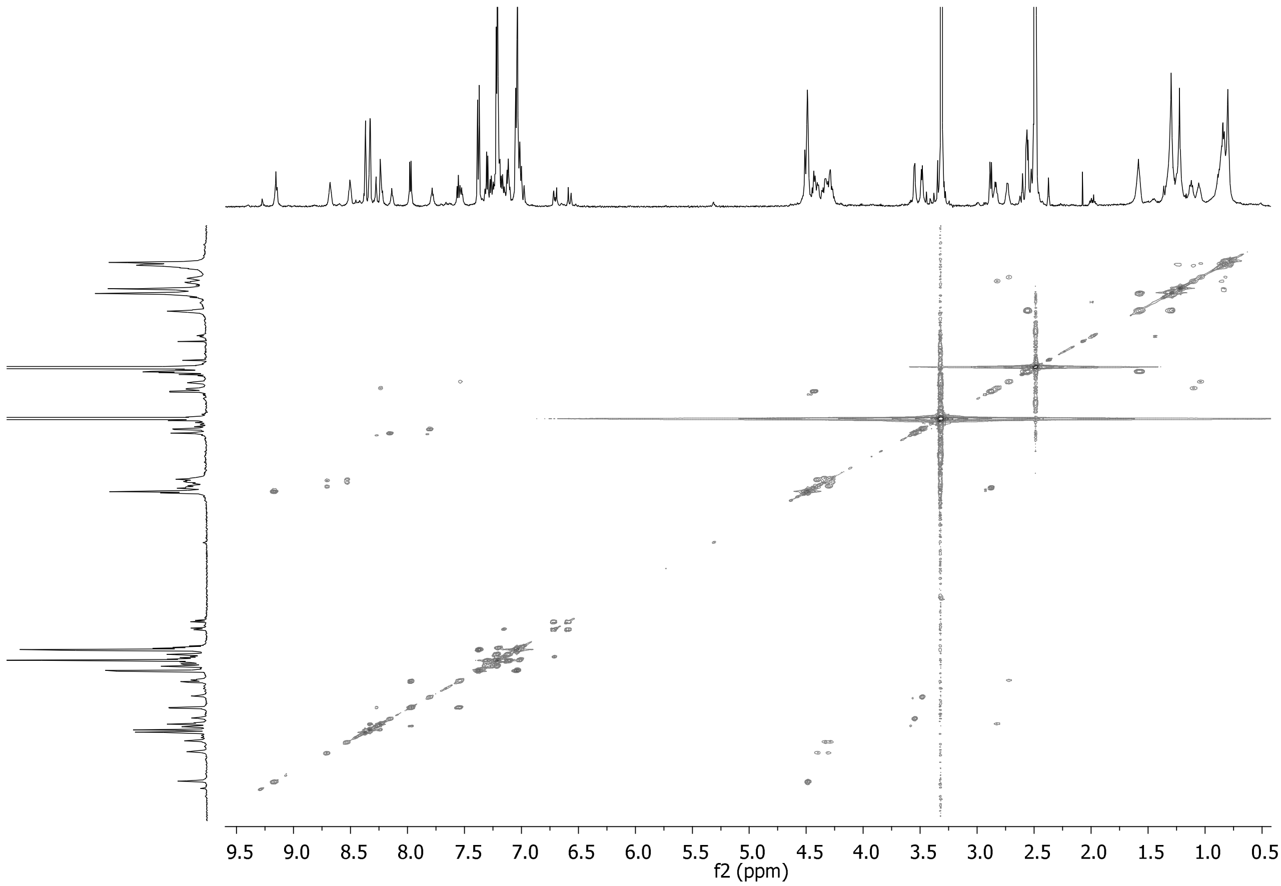

NOESY-NMR of 5 (DMSO-d_6, 298 K)

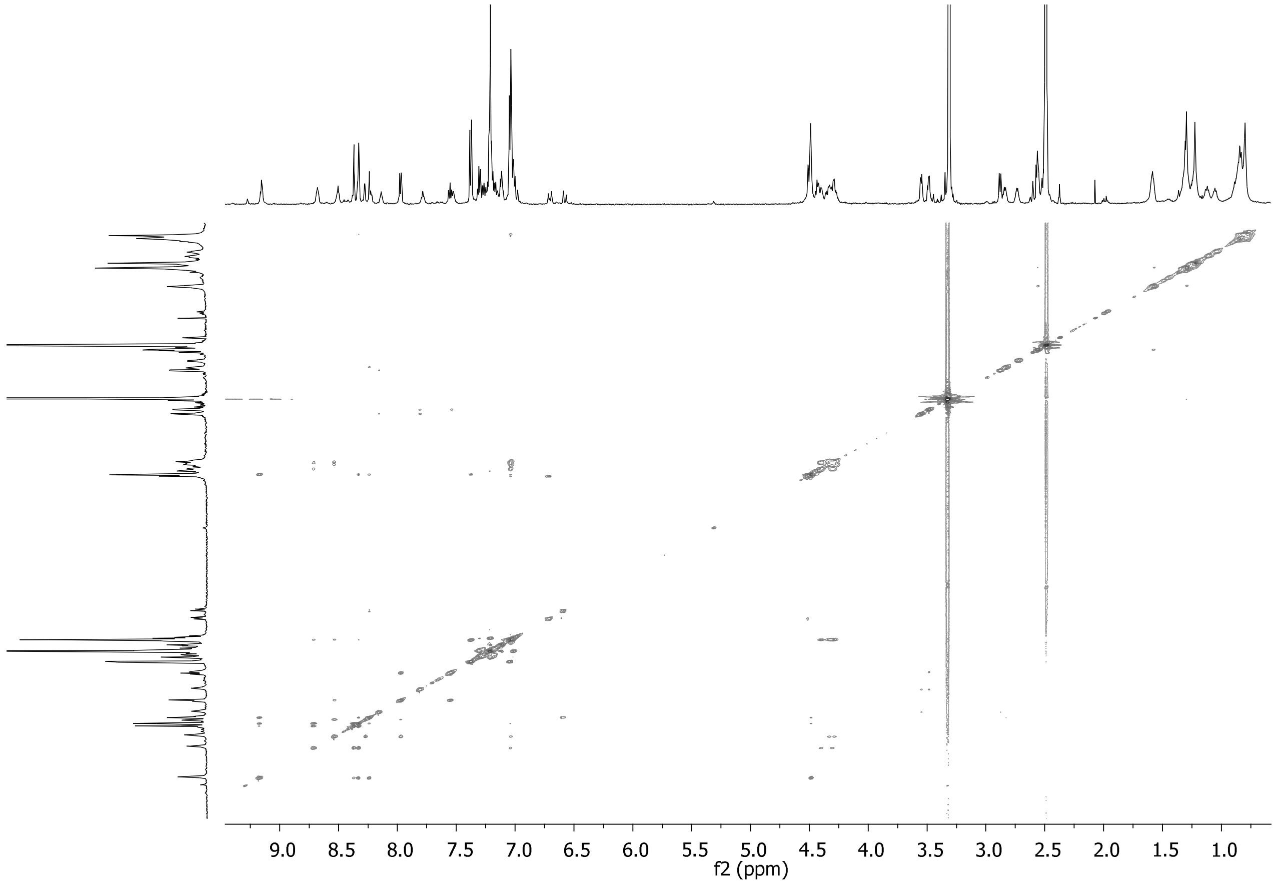

135DEPT-NMR of 5 (DMSO-d_6, 298 K)

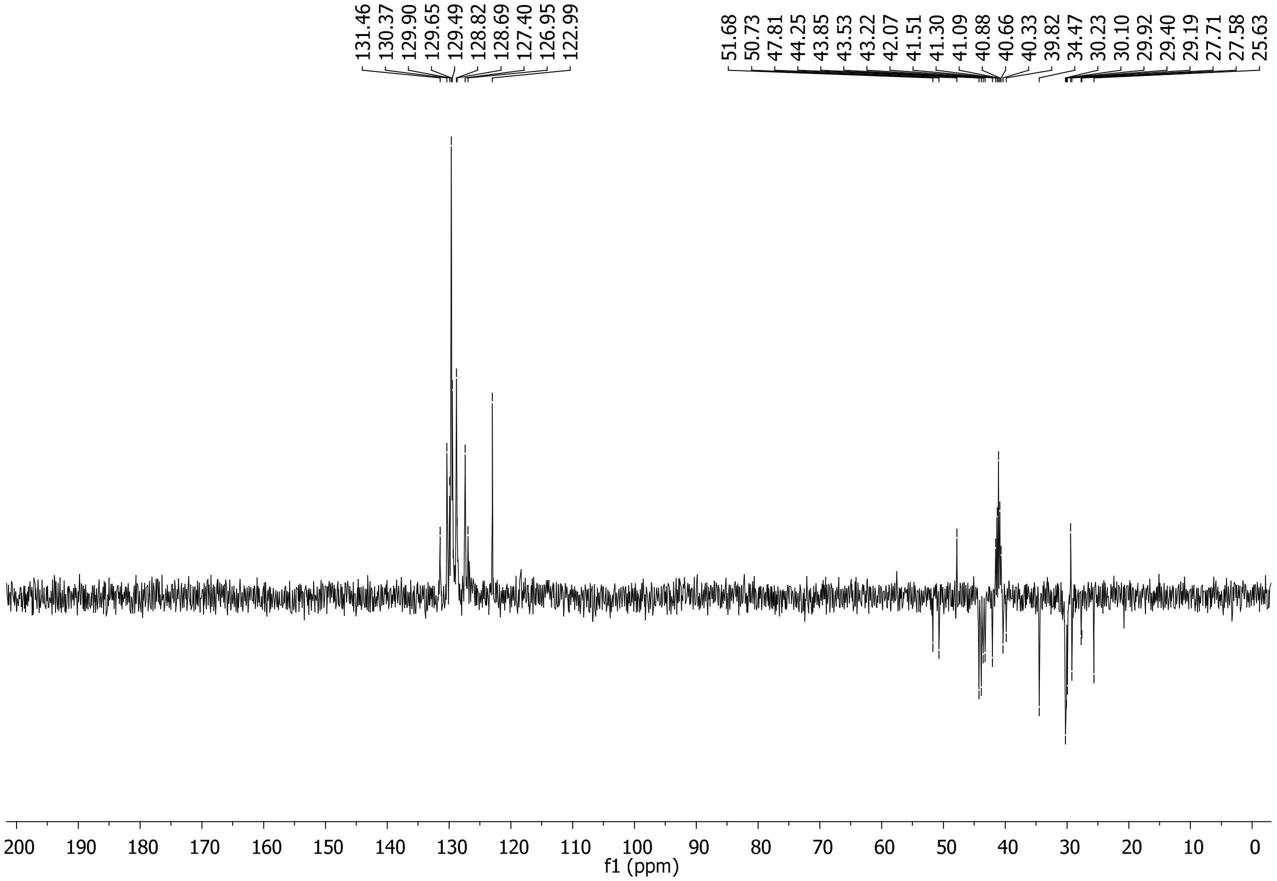

^{1}H-NMR of 5 (600 MHz, CD_2Cl_2, 298 K)

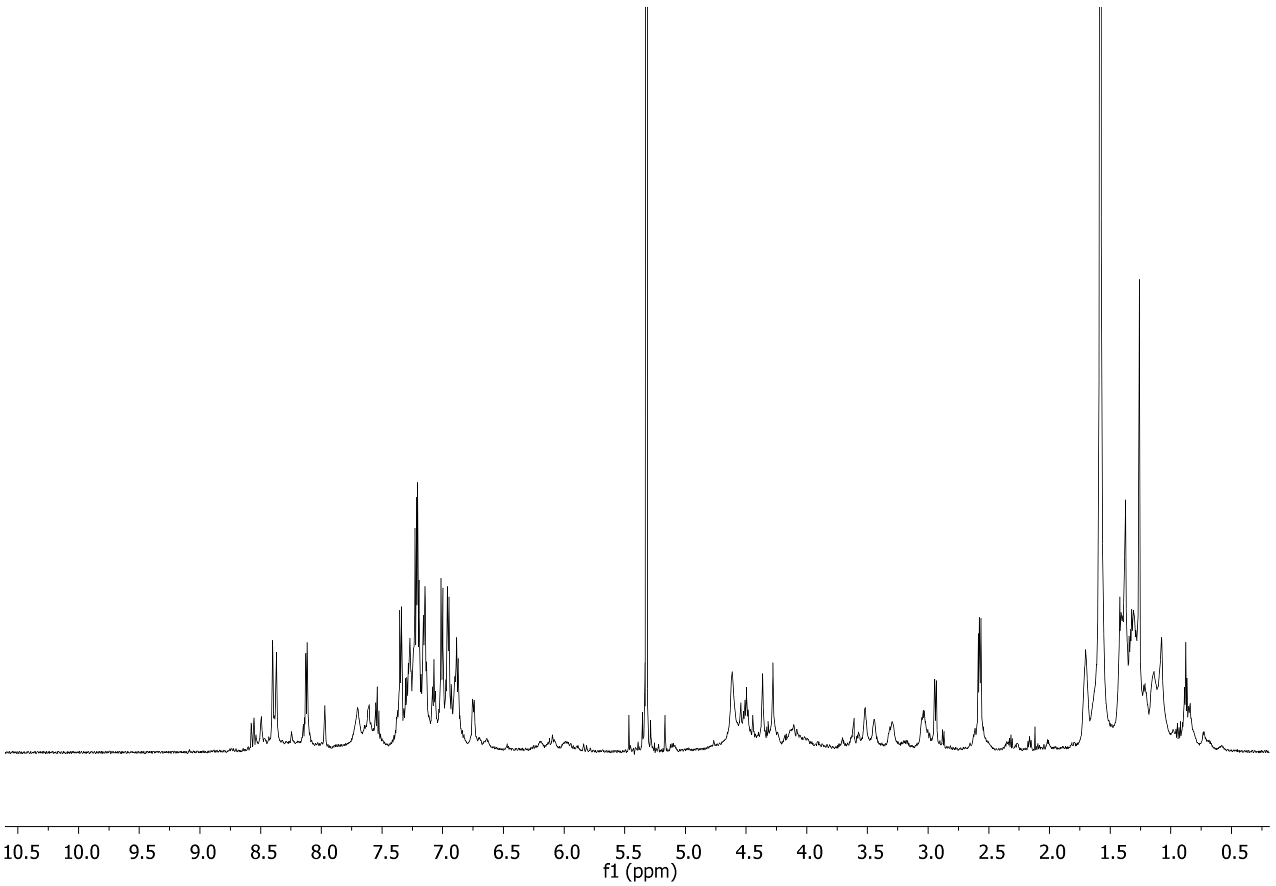

COSY-NMR of 5 (CD_2Cl_2, 298 K)

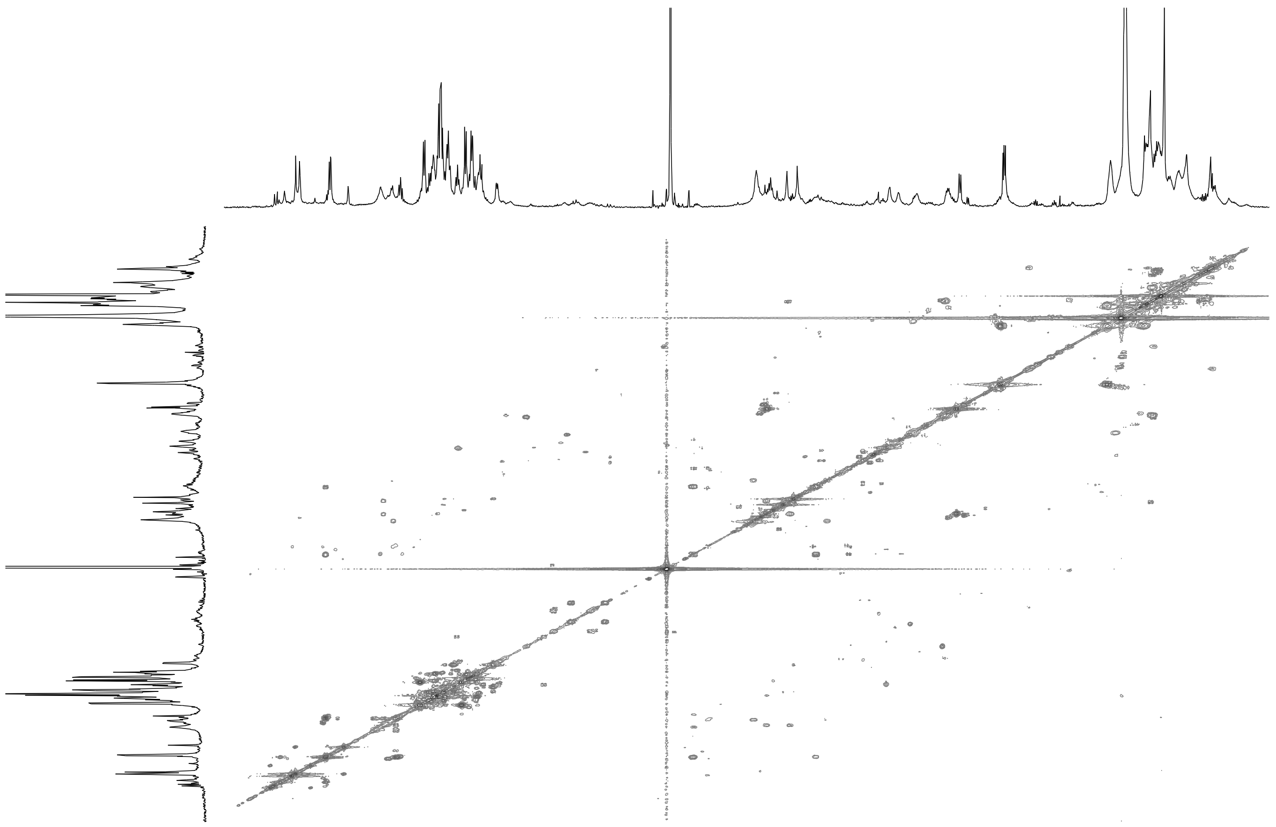

NOESY-NMR of 5 (CD_2Cl_2, 298 K)

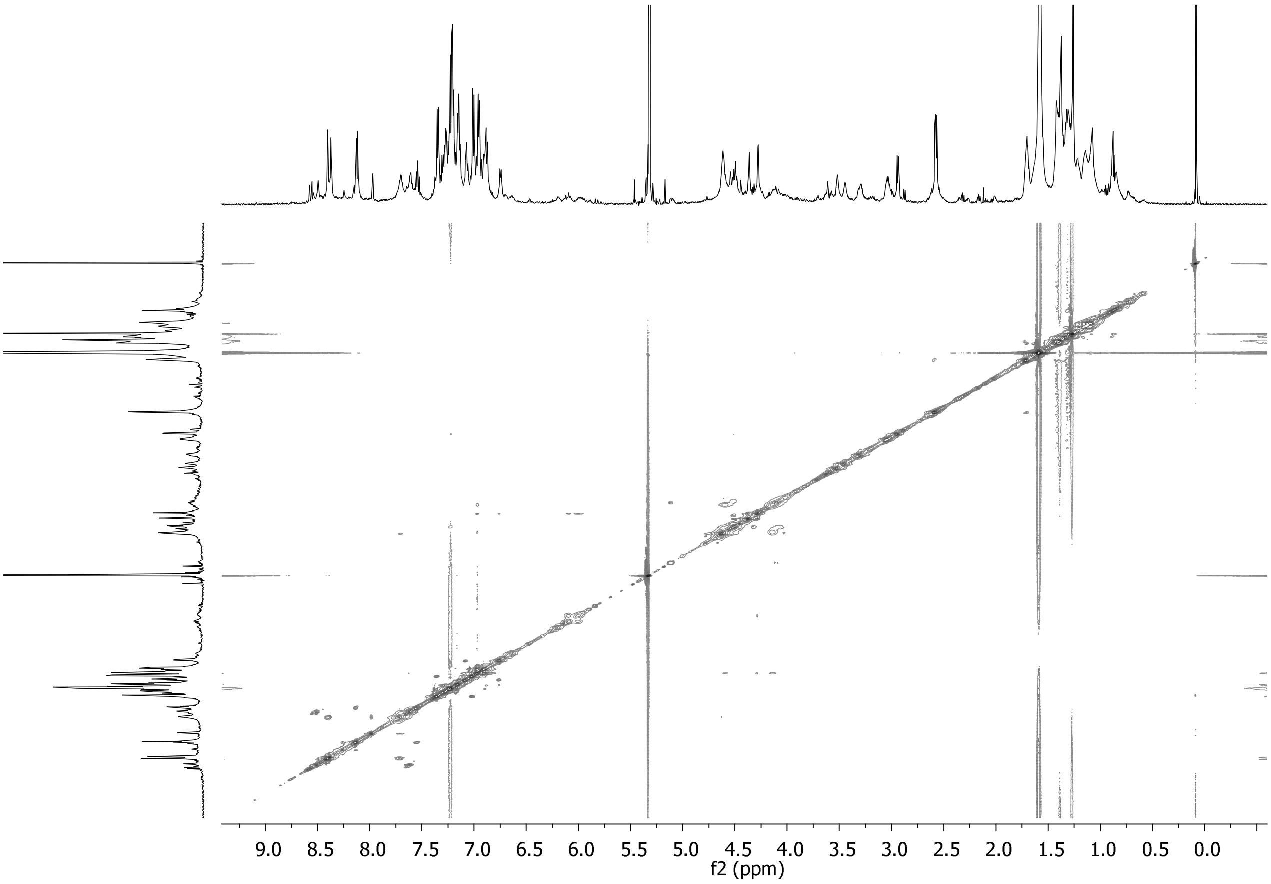

^{1}H-NMR of ***Cp*-1** (400 MHz, $CDCl_3$, 298 K)

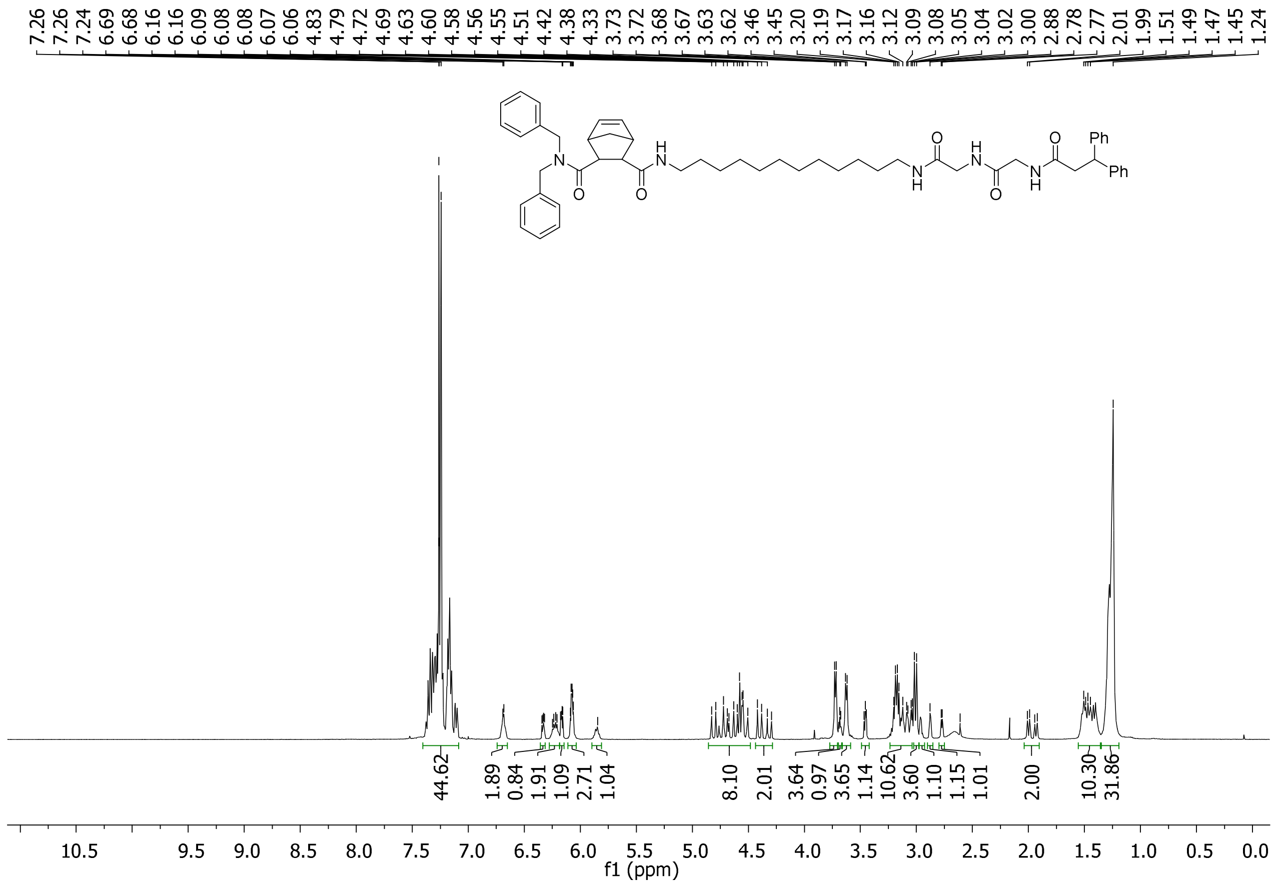

COSY-NMR of ***Cp*-1** ($CDCl_3$, 298 K)

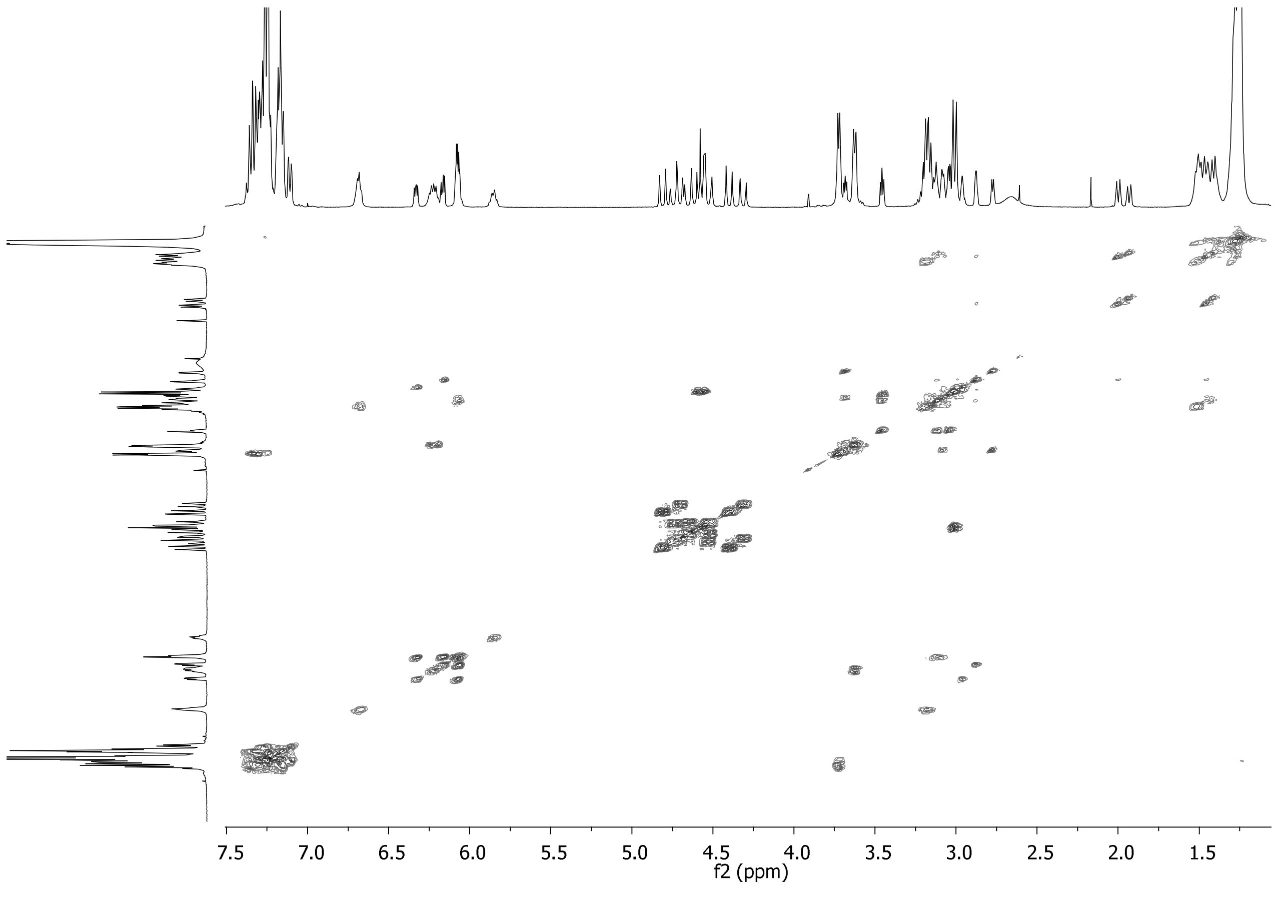

^{13}C-NMR of ***Cp*-1** (100 MHz, $CDCl_3$, 298 K)

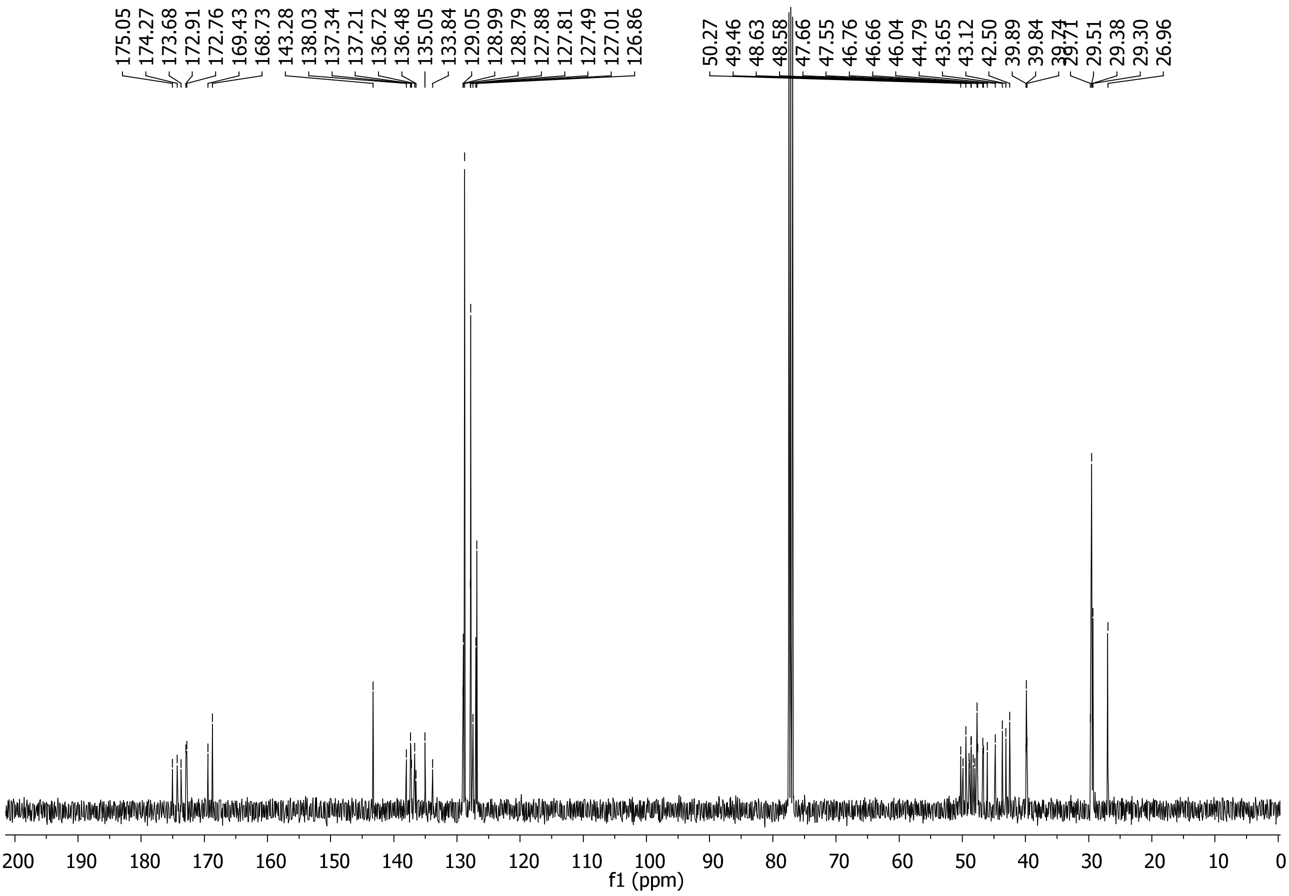

^{135}DEPTof ***Cp*-1** ($CDCl_3$, 298 K)

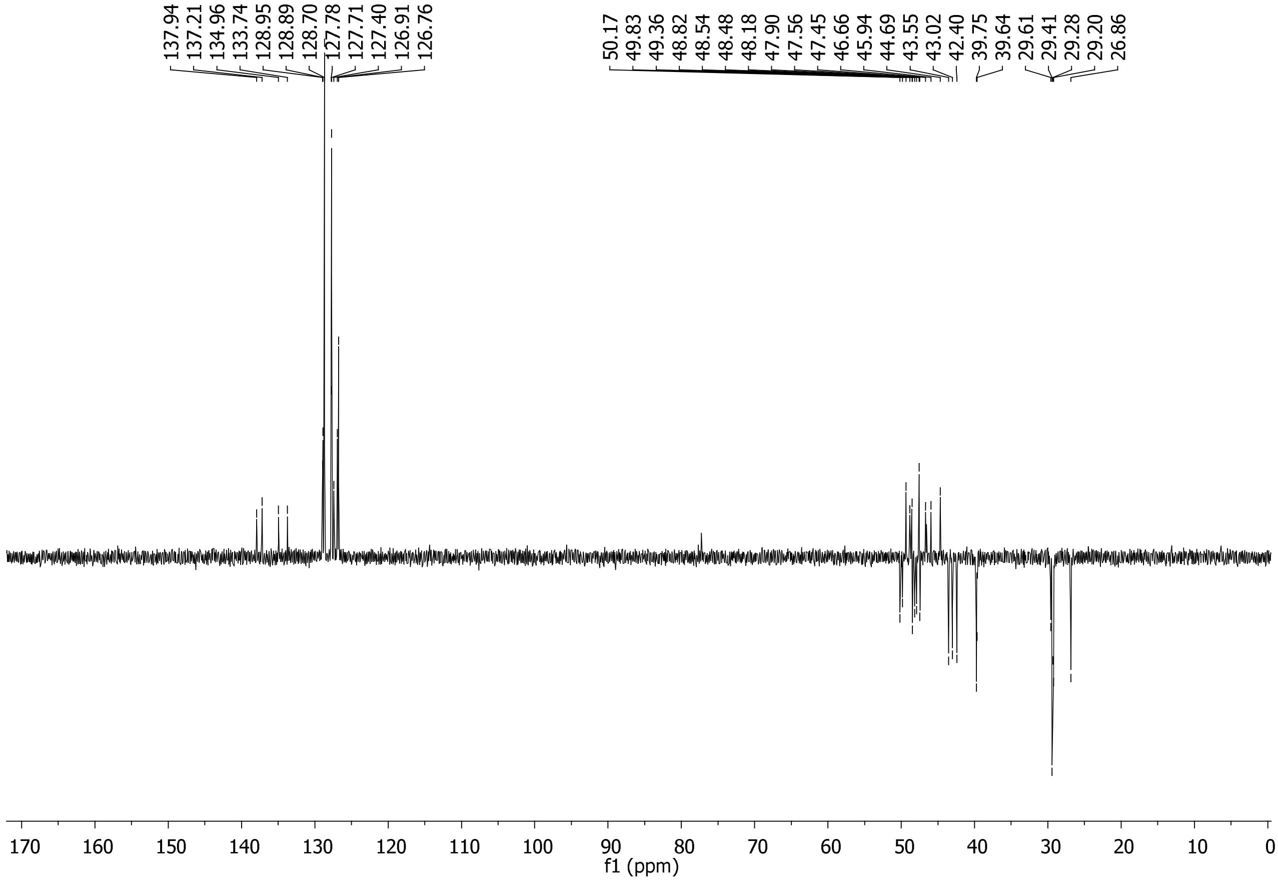

HMQC of ***Cp*-1** ($CDCl_3$, 298 K)

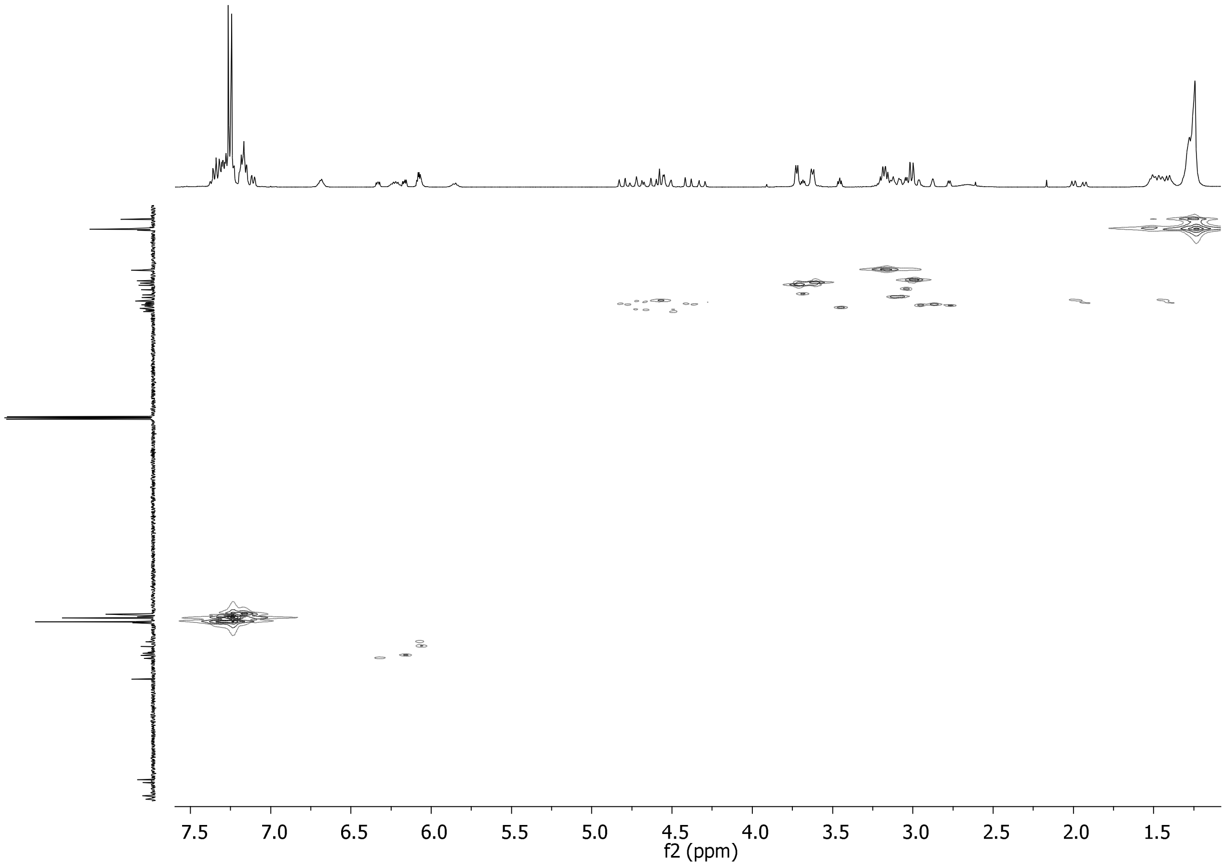

HMBC of ***Cp*-1** ($CDCl_3$, 298 K)

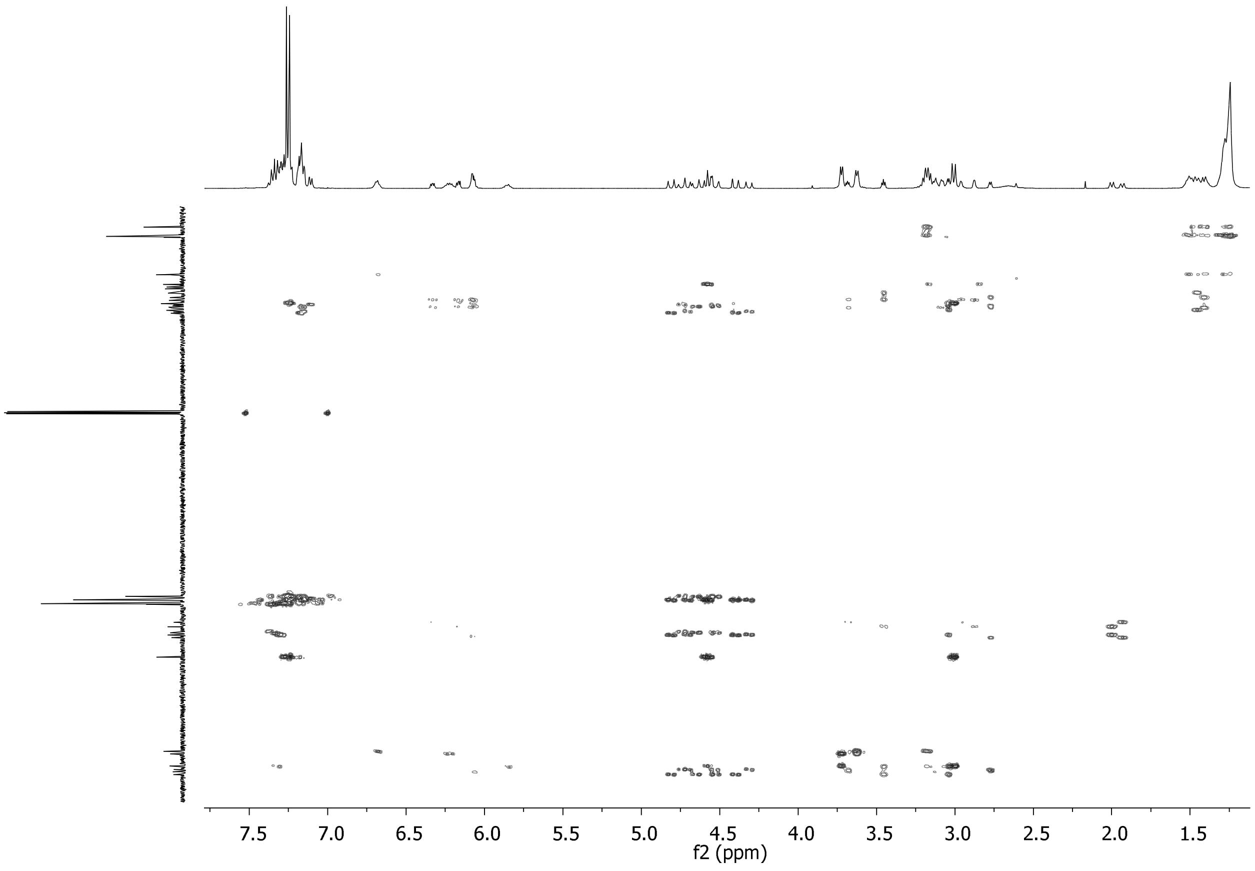

^{1}H-NMR of ***Cp*-2** (400 MHz, $CDCl_3$, 298 K)

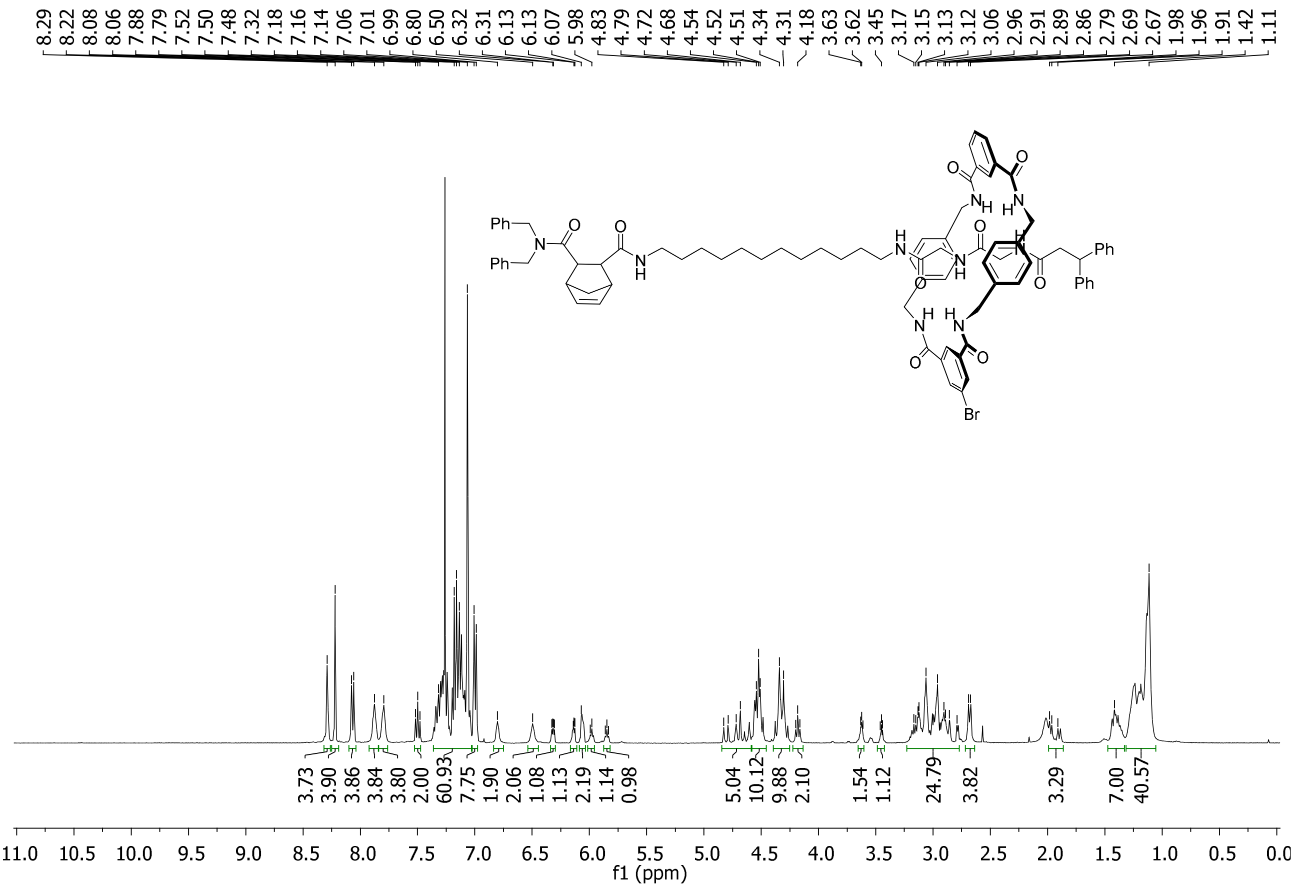

COSY-NMR of ***Cp*-2** ($CDCl_3$, 298 K)

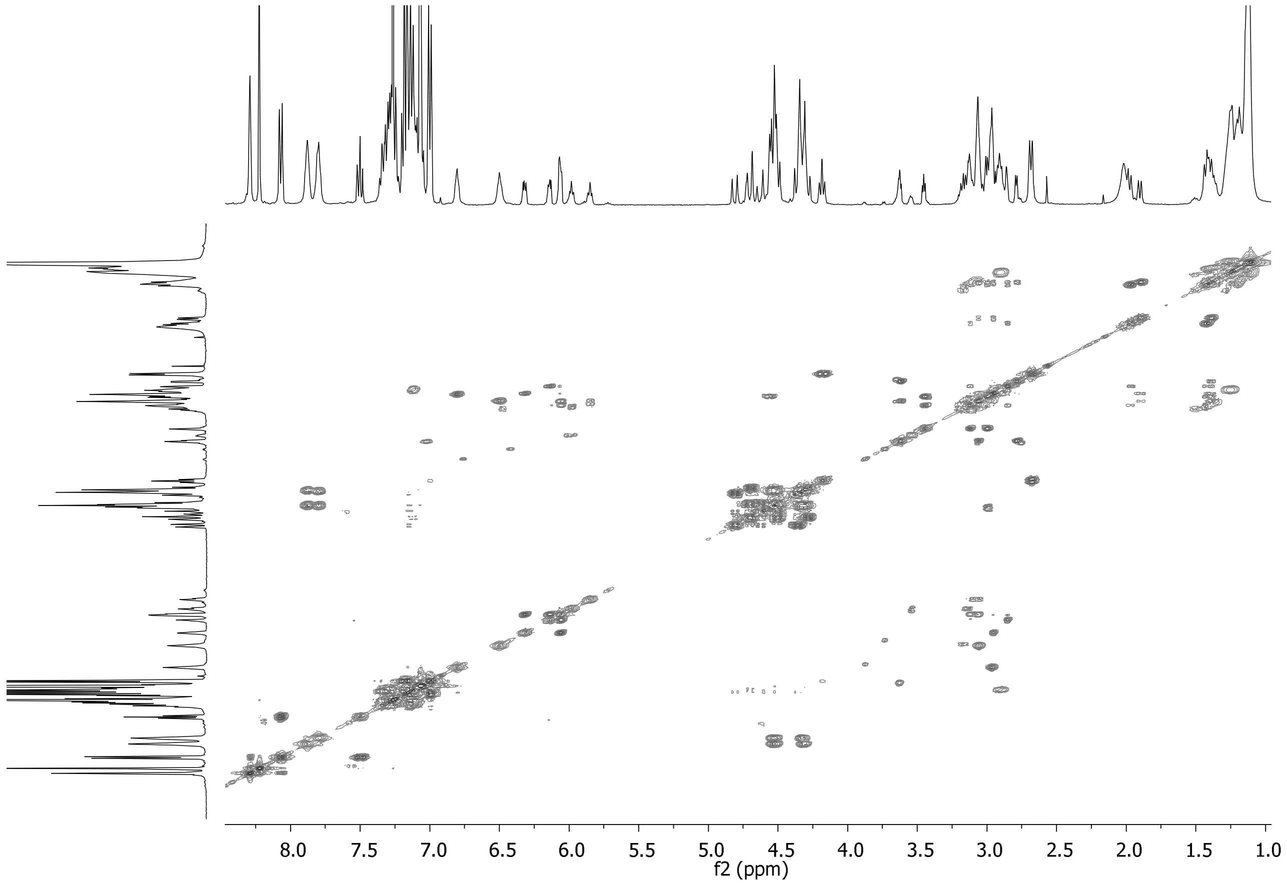

^{13}C-NMR of ***Cp*-2** (100 MHz, $CDCl_3$, 298 K)

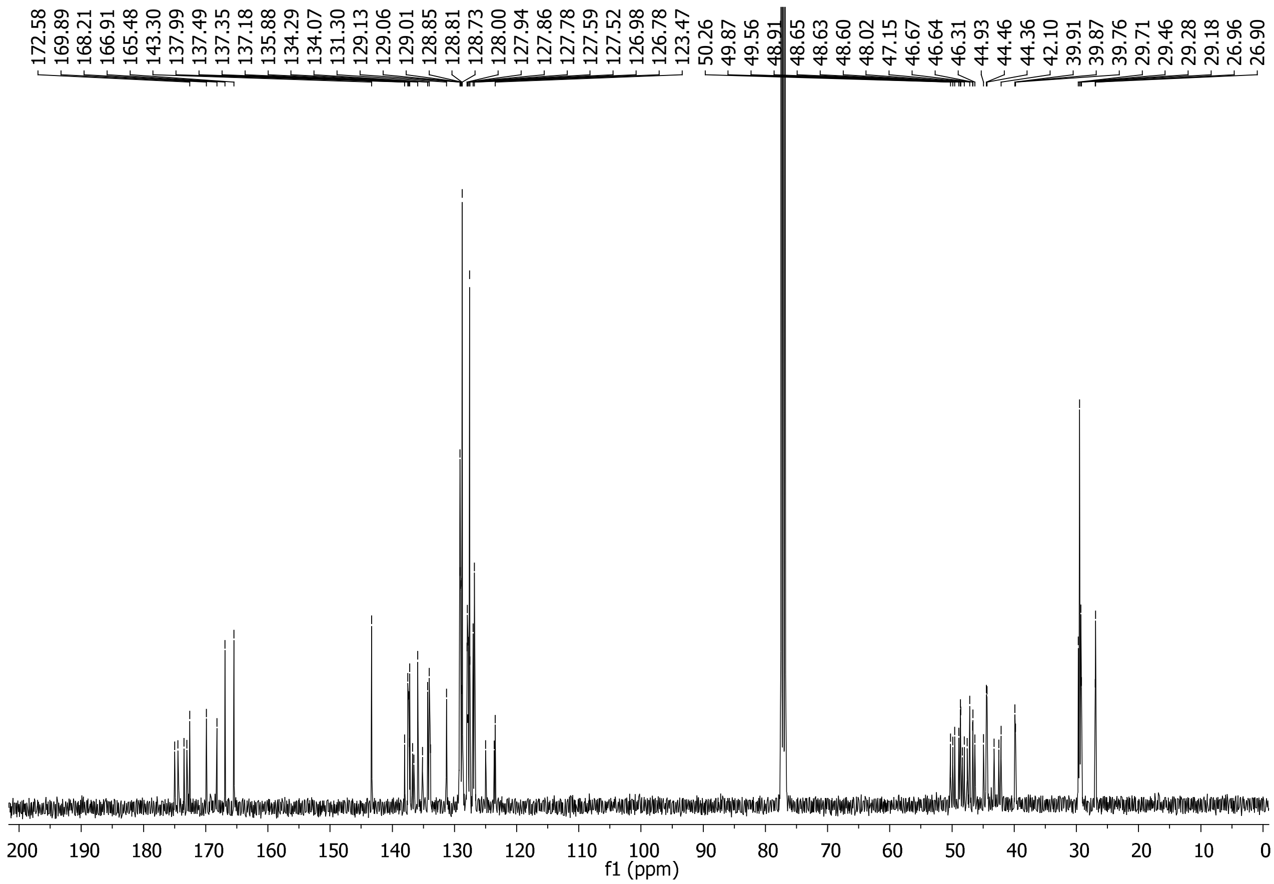

^{135}DEPT-NMR of ***Cp*-2** (100 MHz, $CDCl_3$, 298 K)

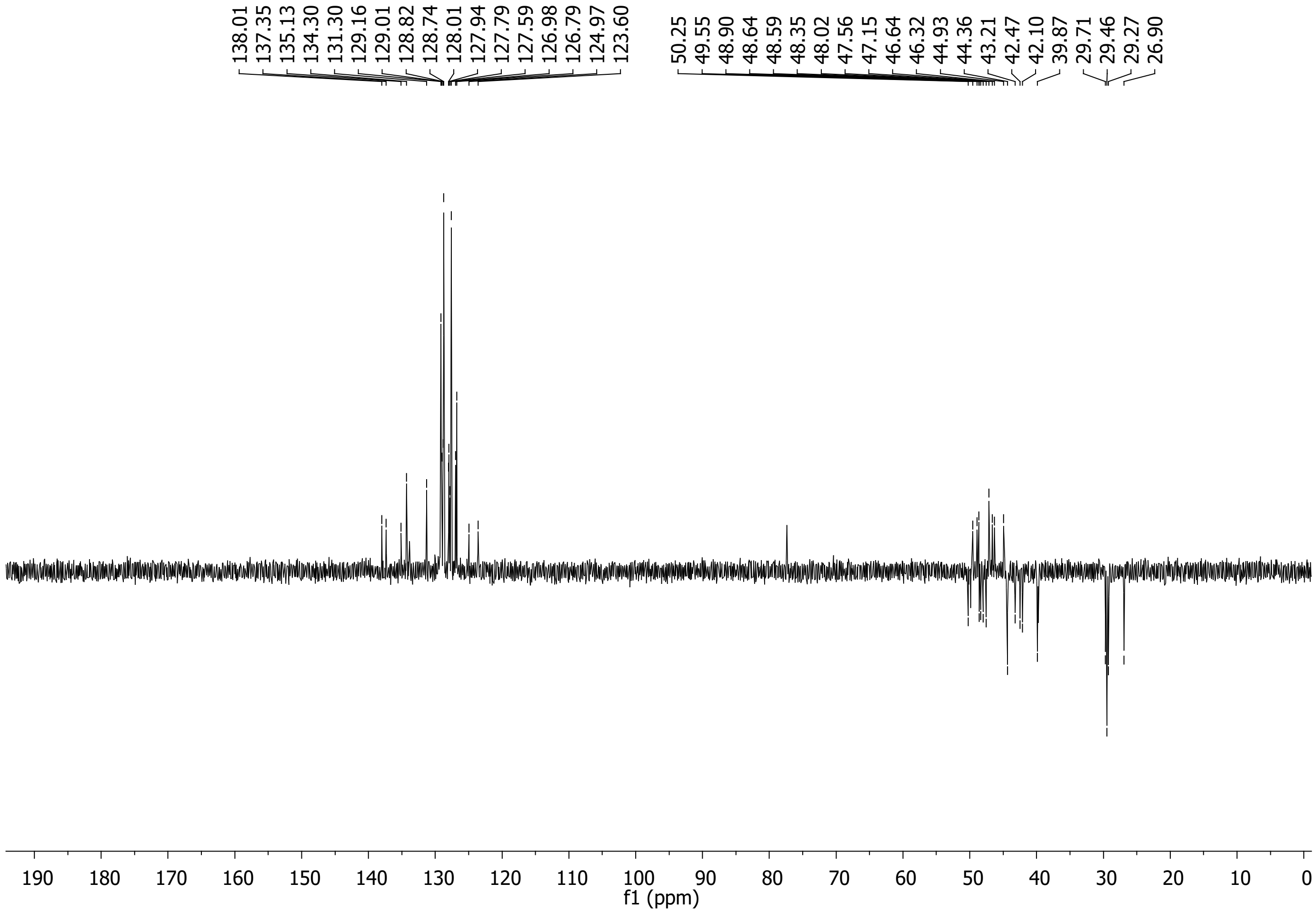

HMQC-NMR of ***Cp*-2** ($CDCl_3$, 298 K)

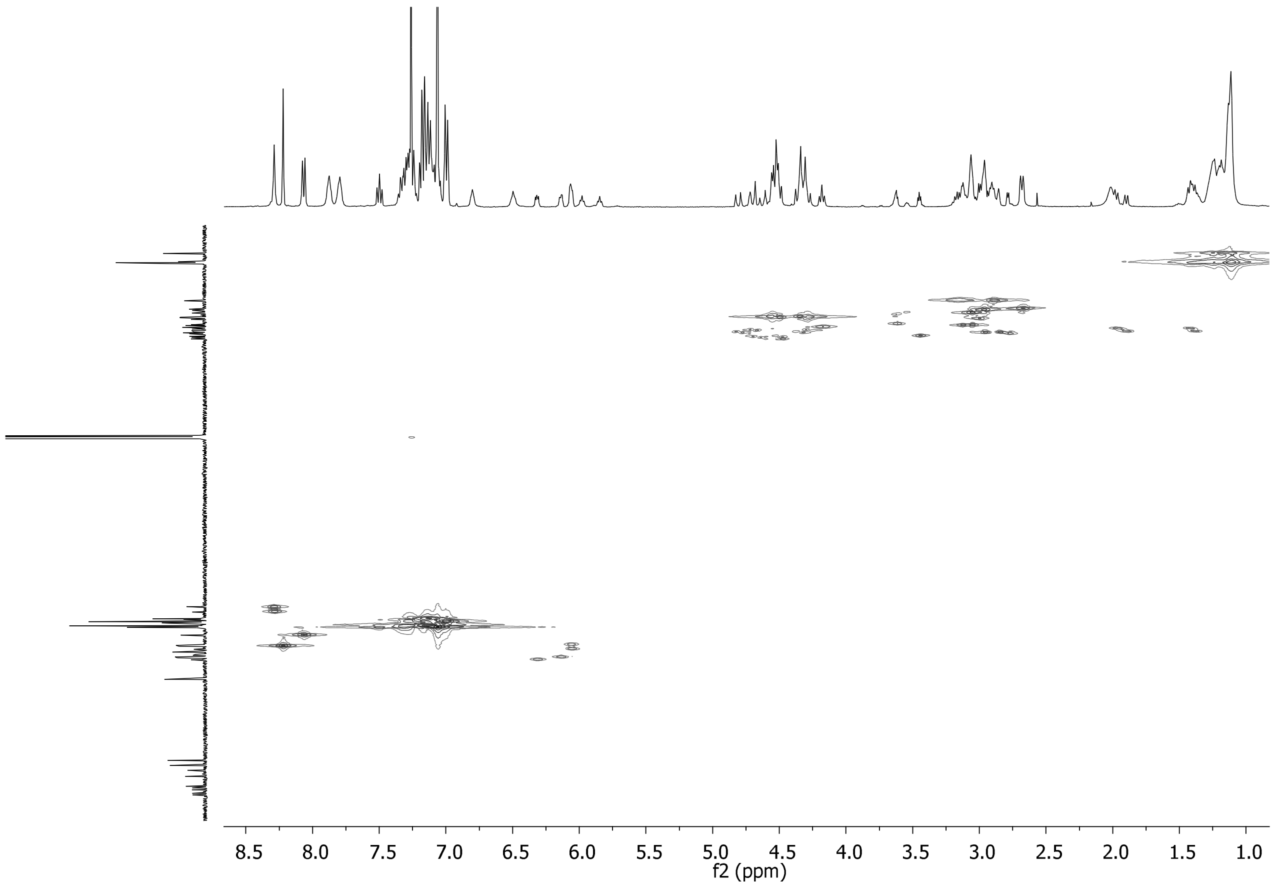

HMBC-NMR of ***Cp*-2** ($CDCl_3$, 298 K)

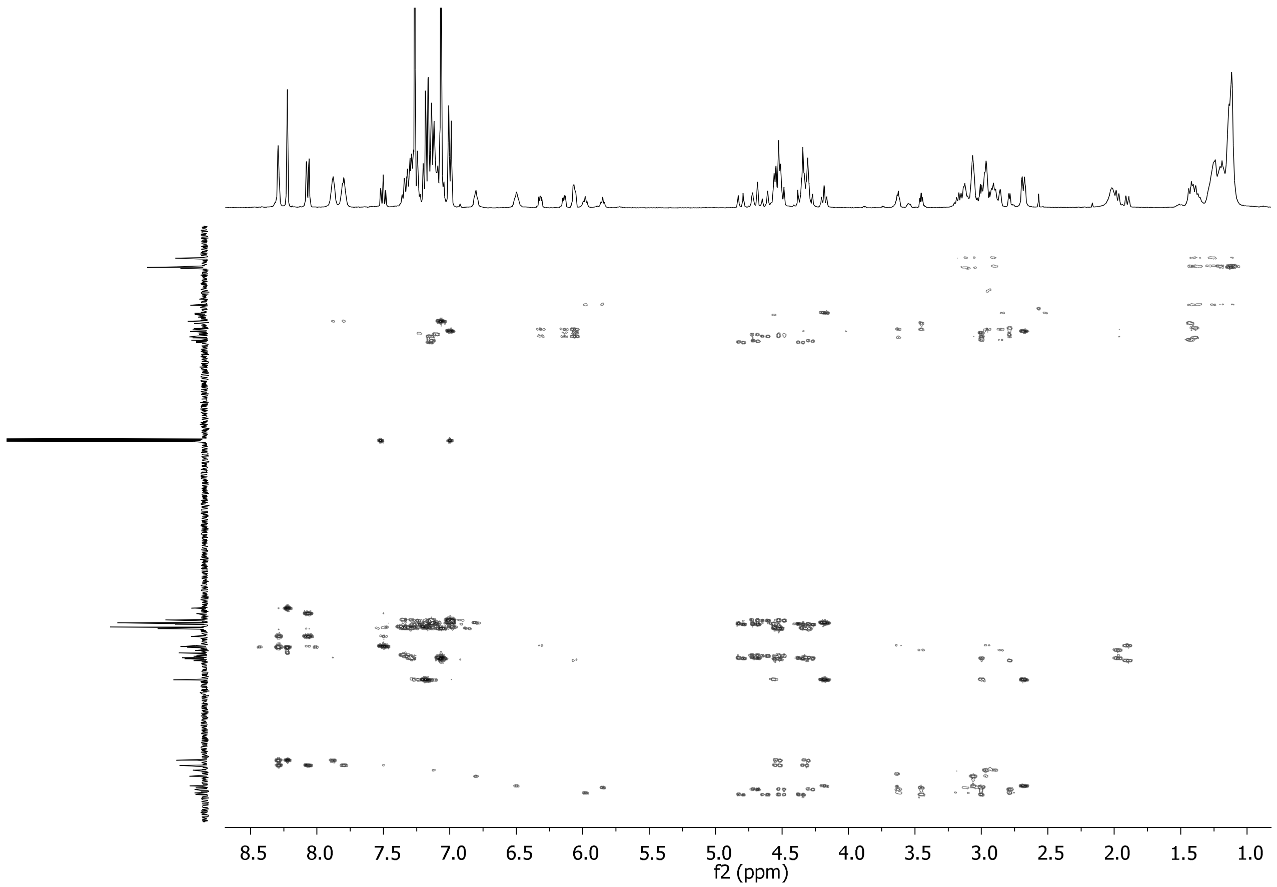

^{1}H-NMR of *Cp*-5 (600 MHz, $CDCl_3$, 298 K)

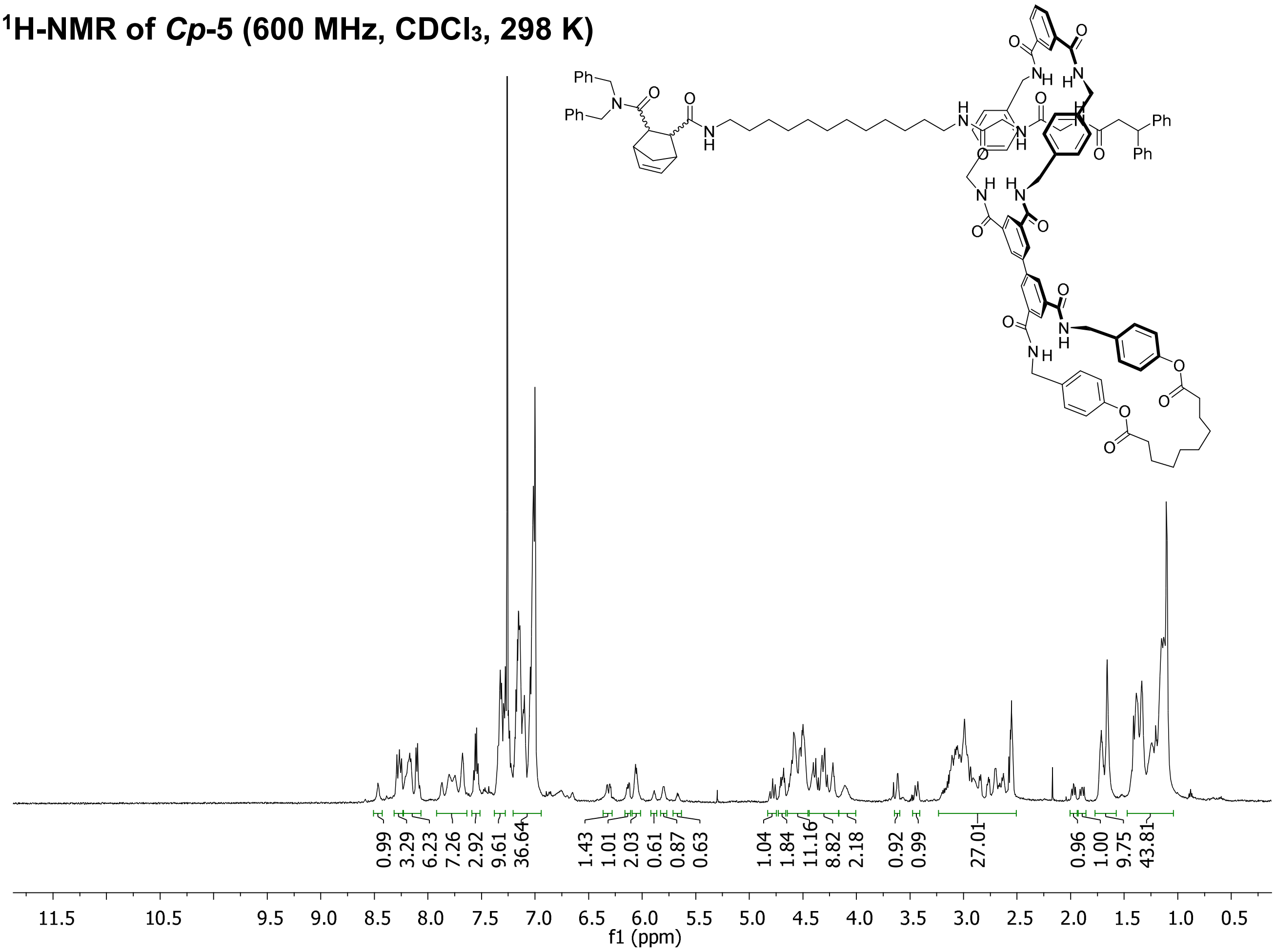

COSY-NMR of *Cp*-5 (600 MHz, $CDCl_3$, 298 K)

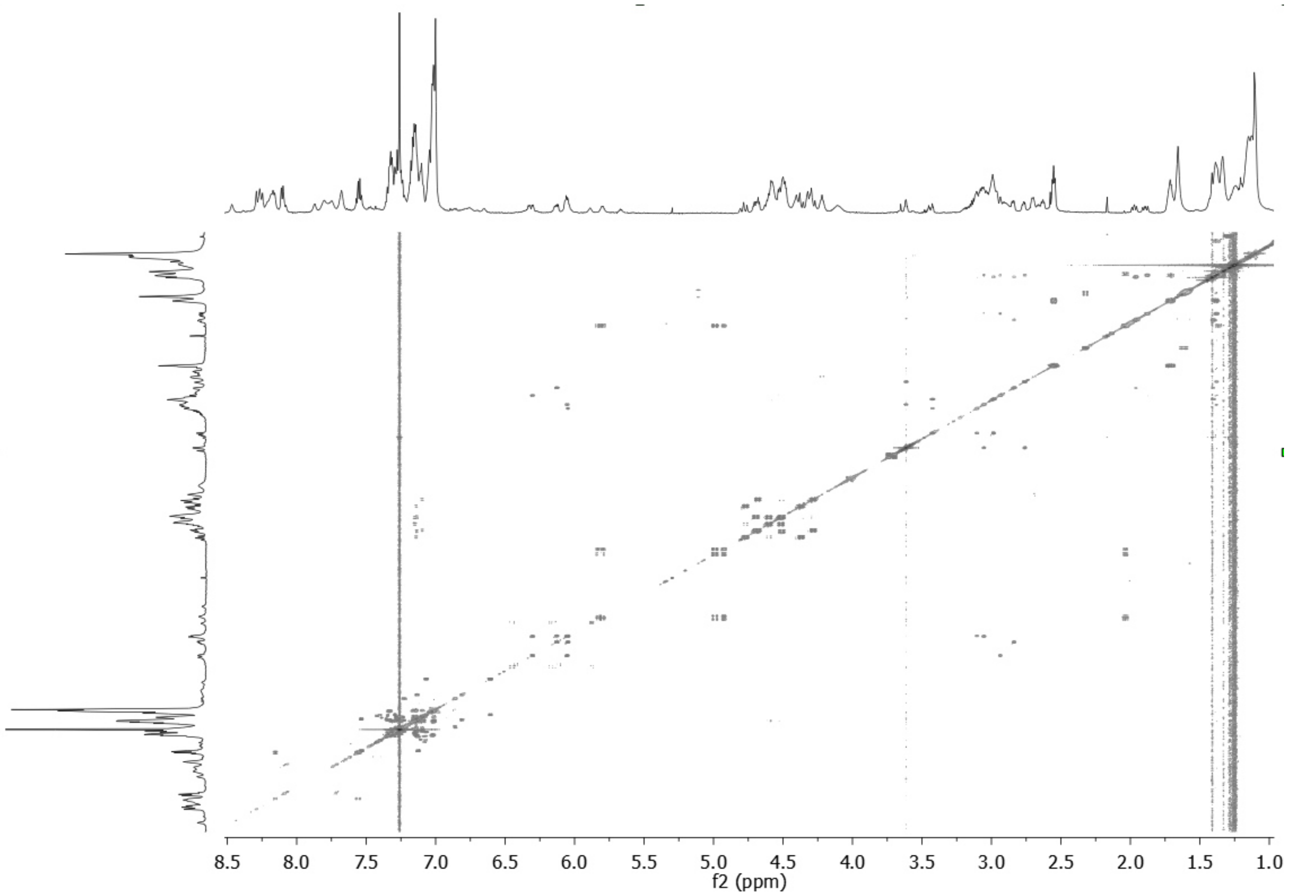

^{13}C-NMR of *Cp*-5 (151 MHz, CDCl$_3$, 298 K)

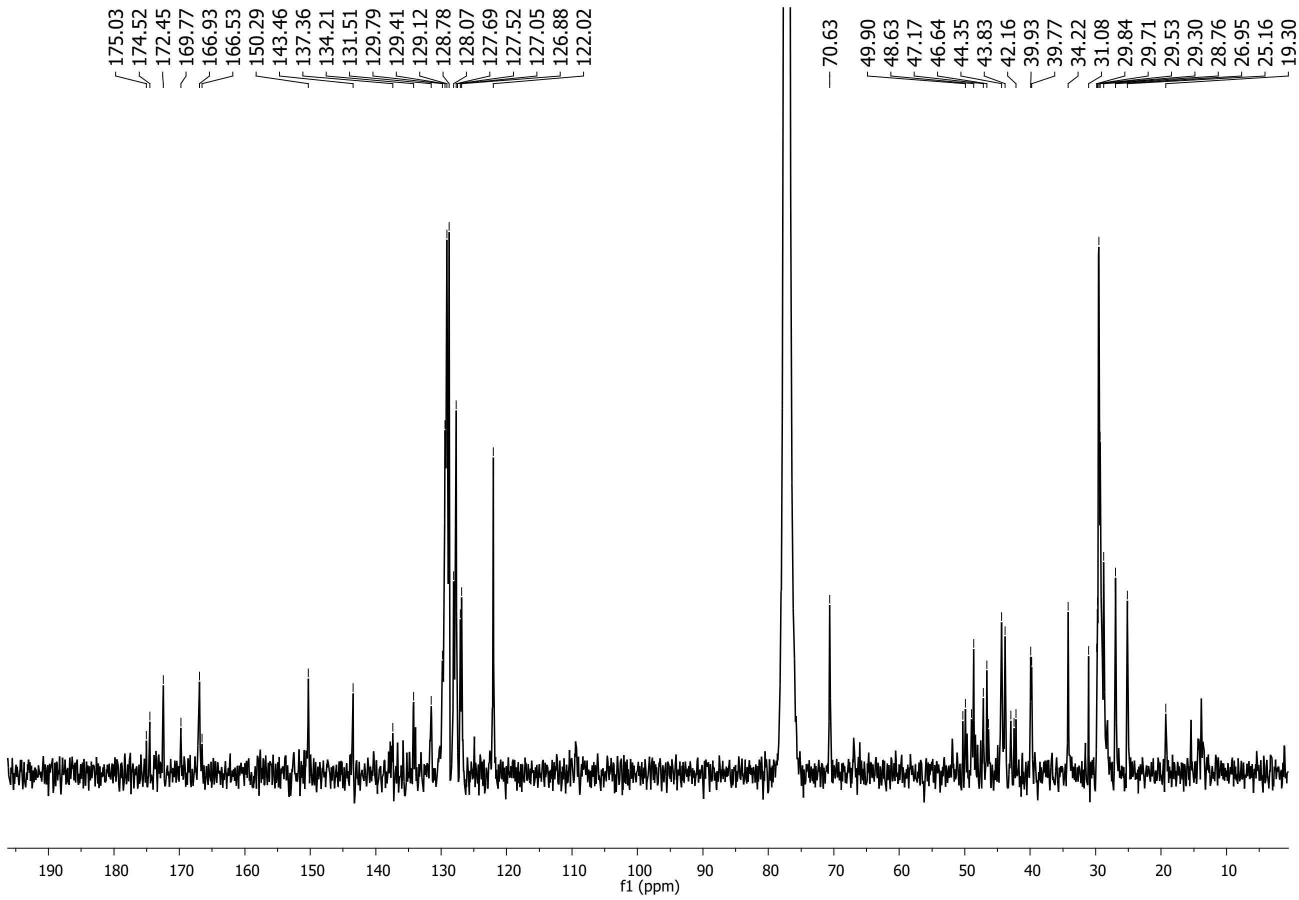

^{135}DEPT-NMR of *Cp*-5 (CDCl$_3$, 298 K)

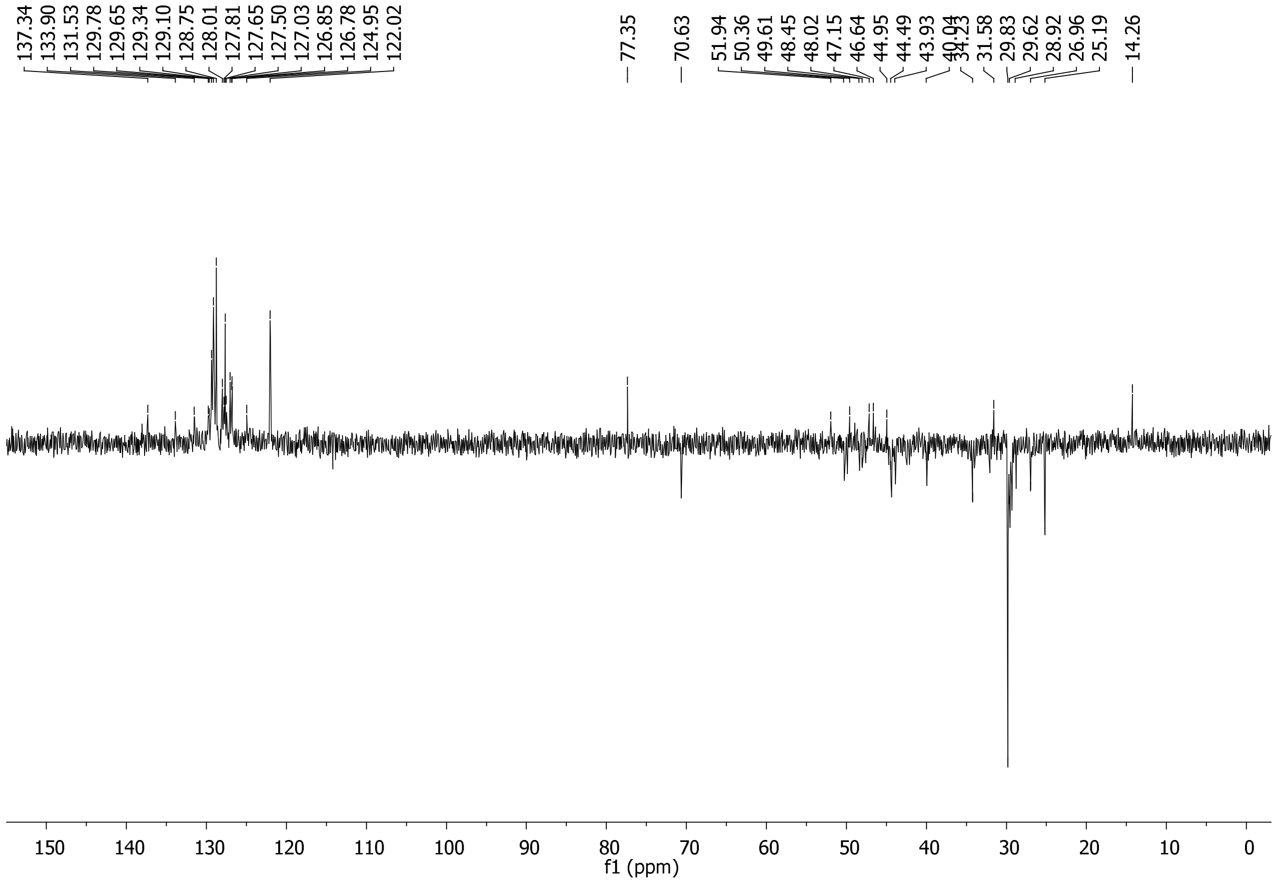

HMQC-NMR of *Cp*-5 (CDCl$_3$, 298 K)

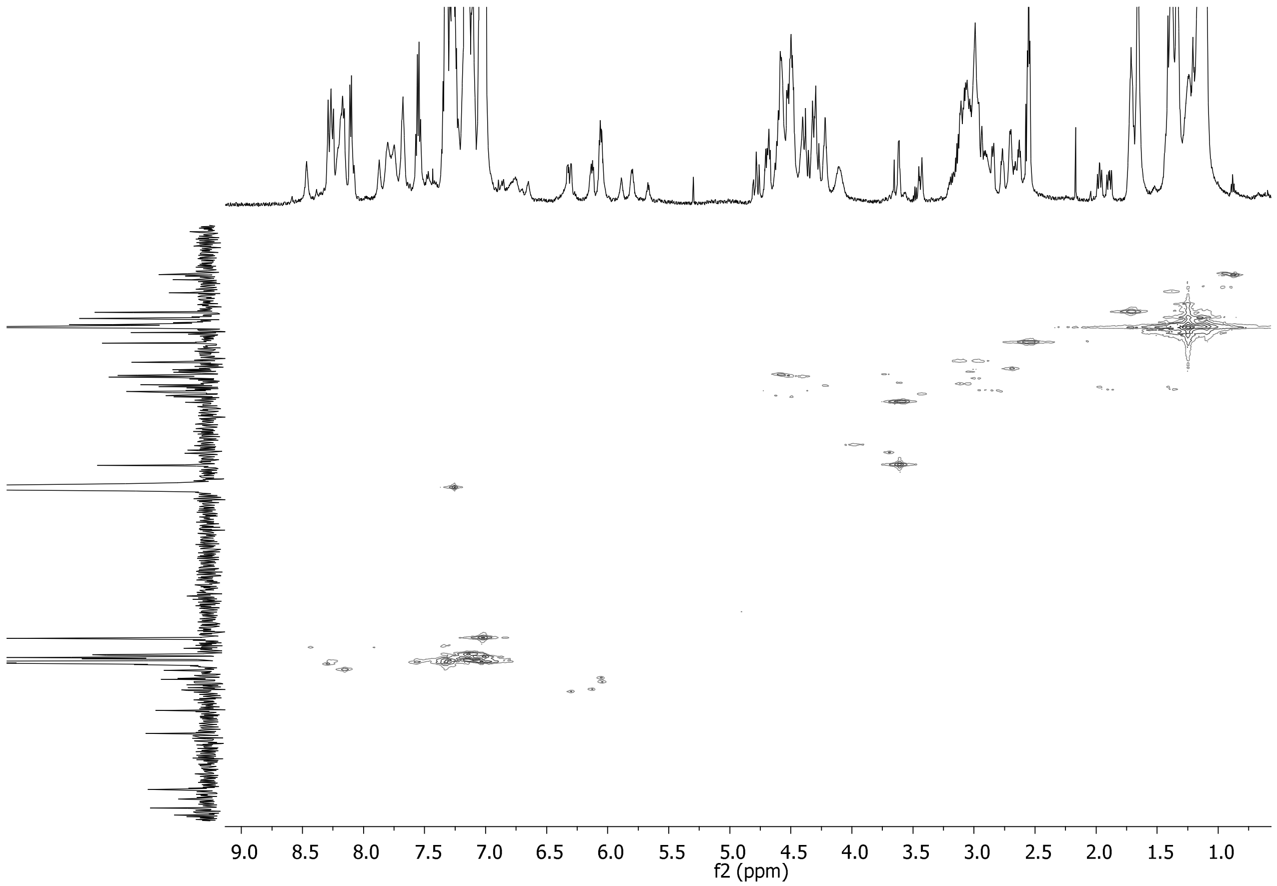

14. Computational Studies.

14.1 Computational Methods

The Global conformational space of the interlocked components, thread **1** and dimacrocyclic hexamide **S7** was analysed by molecular mechanics calculations using the MMFF94 force field.[7] The geometries of the lowest energy conformers were then used as a starting point to carry out geometry optimizations at the DFT level according to the BP86-D3/def2-SVP method.[8]

For the case of interlocked species, [2]rotaxane **5** and [4]pseudorotaxane **5·5**, due to the impracticality of carrying out a conformational analysis, molecular dynamics calculations have been carried out by molecular mechanics using the MMFF94 force field for exploring the different modes of interaction. The molecular dynamics simulations were carried out for 1 ns, with T = 300 K and with a time step of 2 fs. As in the previous approach, the lowest energy of the compounds has been used to carry out the optimizations of their geometries at the DFT level according to the BP86-D3/def2-SVP method, considering solvent effects of chloroform implicitly by means of COSMO[9] continuum solvation model. The BP86 functional was employed with the latest available correction for dispersion (D3),[10] since it is needed to describe adequately the π interactions. This level of theory is a good compromise between the size of the systems and the accuracy of the results.[11]

The interaction energy has been calculated as the difference between the energy of the supramolecular complex minus the energies of all the molecules that form it, in its lowest energy conformation. For example, the interaction energy for the [2]rotaxane **5** has been calculated as the difference between the energy of the rotaxane minus the energies of thread **1** and dimacrocyclic hexamide **S7**.

MMFF94 based calculations have been performed by using ChemBio3D program and DFT calculations have been carried out by utilizing the program TURBOMOLE version 7.2.[12]

14.2 Cartesian coordinates

Only the cartesian coordinates of the lowest energy conformer are shown.

Thread **1**

C	0.3267424	0.9829215	0.6085659
C	-0.7647222	0.7425530	-0.4560769
C	-1.9490714	0.0042822	0.1390403
N	-2.1576666	-1.2517068	-0.3323607
C	-3.2855652	-2.0536597	0.1050464
C	-3.3829251	-2.2813525	1.6212910
N	-2.1946943	-2.3481126	2.2920790
C	-2.1407367	-2.4385898	3.7359045
C	-2.1099684	-1.0989422	4.4983147
N	-2.4357063	-0.0026617	3.7743136
C	-2.6016222	1.3146348	4.3661328
C	-4.0365420	1.5532535	4.8733149
C	-5.1396423	1.3895345	3.8123824
C	-5.0652619	2.3956186	2.6489145
C	-6.3799599	2.5429535	1.8569273

C	-6.9393361	1.2459353	1.2384426
C	-6.0035282	0.5415578	0.2425939
C	-5.7536051	1.3009417	-1.0715637
C	-4.6404869	0.6596500	-1.9109357
C	-4.3160848	1.3547990	-3.2441556
C	-2.8782848	1.0988297	-3.7297961
C	-2.5337760	-0.3805379	-3.9792867
N	-1.1238447	-0.5403354	-4.3229533
C	-0.1983763	-1.1477075	-3.5321074
C	1.2189031	-0.9140398	-3.9302499
C	2.2183150	-1.1540548	-3.0544029
C	3.6452610	-0.8760703	-3.4075424
N	4.5412601	-0.8724268	-2.3632324
C	5.9776436	-0.7871784	-2.6675510
C	4.1784196	-0.7869867	-0.9510955
O	-2.6510581	0.5155273	1.0331157
O	-4.4835531	-2.4248734	2.1624785
O	-1.8319674	-1.0857722	5.7066845
O	3.9933799	-0.7016725	-4.5878230
O	-0.4900821	-1.8053221	-2.5075801
C	0.6636707	-0.2954877	1.3761043
C	0.9448029	-1.5018477	0.6973384
C	1.2750356	-2.6659694	1.4086004
C	1.3316379	-2.6429645	2.8137556
C	1.0412418	-1.4529712	3.5012199
C	0.7053937	-0.2909414	2.7847601
C	1.6036132	1.6041321	0.0295954
C	1.8132700	1.7852782	-1.3534822
C	3.0306225	2.2997475	-1.8389439
C	4.0517034	2.6620243	-0.9488313
C	3.8488125	2.5021838	0.4346108
C	2.6421483	1.9734438	0.9150137
C	6.5114551	0.6313921	-2.5806430
C	7.2880165	1.0474359	-1.4812248
C	7.7563924	2.3713158	-1.3945091
C	7.4497201	3.2927867	-2.4106011
C	6.6686545	2.8861693	-3.5103217
C	6.2004900	1.5655556	-3.5937197
C	4.3250817	-2.0765903	-0.1673763
C	4.5627181	-2.0176386	1.2206978
C	4.6210835	-3.1923199	1.9858326
C	4.4459398	-4.4449467	1.3705892
C	4.2140377	-4.5127210	-0.0142838
C	4.1550705	-3.3340677	-0.7787677
H	-0.0922766	1.6986262	1.3474160
H	-0.3594789	0.1692201	-1.3064826
H	-1.1251694	1.7180773	-0.8397125
H	-1.5718190	-1.5776125	-1.1263592
H	-4.2490368	-1.5920244	-0.1856936
H	-3.2215491	-3.0403429	-0.3944589
H	-1.3256279	-2.1647783	1.7791820
H	-3.0494554	-2.9726452	4.0821943
H	-1.2573193	-3.0235863	4.0536557
H	-2.6191727	-0.0768927	2.7613032
H	-2.3243131	2.0639458	3.5965500
H	-1.8874833	1.4080133	5.2102112
H	-4.2193424	0.8447426	5.7097102
H	-4.0844119	2.5764802	5.3094965
H	-5.1118440	0.3507448	3.4151130
H	-6.1243513	1.4940293	4.3210631
H	-4.7941457	3.3970529	3.0544660
H	-4.2387718	2.1105129	1.9640849
H	-7.1562779	2.9682388	2.5327534
H	-6.2332342	3.3039586	1.0586393
H	-7.1834057	0.5327203	2.0551849
H	-7.9085396	1.4766134	0.7405968
H	-5.0285751	0.3493297	0.7381242
H	-6.4192604	-0.4630793	0.0037912
H	-6.6980534	1.3670634	-1.6586562
H	-5.4589963	2.3516534	-0.8509440

H	-3.7243928	0.6583498	-1.2878417
H	-4.8822229	-0.4120439	-2.0906466
H	-5.0461253	1.0531240	-4.0277835
H	-4.4386056	2.4534785	-3.1237102
H	-2.6859710	1.6743137	-4.6618256
H	-2.1644474	1.4889165	-2.9710968
H	-2.7173643	-0.9893190	-3.0743880
H	-3.1658257	-0.7973034	-4.7931869
H	-0.7798128	-0.0189514	-5.1340250
H	1.4536870	-0.4781779	-4.9159441
H	1.9637284	-1.5867472	-2.0756696
H	6.5081657	-1.4472518	-1.9509638
H	6.1211481	-1.1919168	-3.6872435
H	3.1422169	-0.4085145	-0.8554652
H	4.7950313	0.0135374	-0.4922286
H	0.8750716	-1.5452257	-0.3998541
H	1.5130066	-3.5899542	0.8612519
H	1.5981578	-3.5545402	3.3689784
H	1.0408362	-1.4301095	4.6011087
H	0.4517038	0.6306633	3.3304824
H	1.0379756	1.5029048	-2.0782521
H	3.1864560	2.3999578	-2.9240690
H	5.0075617	3.0470397	-1.3306686
H	4.6451238	2.7781372	1.1428399
H	2.5025440	1.8277692	1.9976568
H	7.5337284	0.3250853	-0.6862146
H	8.3621531	2.6833210	-0.5299348
H	7.8146442	4.3293344	-2.3452224
H	6.4229872	3.6056815	-4.3066382
H	5.5809551	1.2375415	-4.4422717
H	4.6799993	-1.0368293	1.7082141
H	4.7961290	-3.1295591	3.0706038
H	4.4906020	-5.3670246	1.9701643
H	4.0790568	-5.4891678	-0.5048704
H	3.9815373	-3.3905689	-1.8647600

Dimacrocyclic Hexamide **S7**

C	11.9625491	6.1166853	-1.2200948
C	13.1453780	5.3611903	-0.5862485
C	12.8163851	4.0067067	0.0612090
C	12.2651724	2.9384283	-0.8927673
C	11.9177551	1.6341491	-0.1643765
C	11.3805475	0.5213298	-1.0725396
C	11.0635782	-0.7778137	-0.3127800
O	10.1831985	-1.1896887	1.9215794
C	10.1007755	-0.6312522	0.8476992
C	10.7727572	6.3741744	-0.2590044
O	9.5575425	4.5141658	0.7640043
C	9.7790405	5.2352379	-0.1901734
O	9.0727847	0.2348430	0.5290379
O	9.1273613	5.1311782	-1.3980922
O	2.5966269	-0.2295537	3.8919432
O	2.5360103	1.6927705	-2.9516293
C	6.9858567	2.1406188	2.8898333
C	8.0065018	1.8220440	1.9783828
C	8.0945290	0.5114321	1.4917169
C	7.1908305	-0.4832831	1.9011513
C	6.1771993	-0.1486496	2.8103723
C	7.4808600	1.8192311	-1.7591589
C	8.4045082	2.7818267	-1.3265899
C	8.2018980	4.1256260	-1.6779401
C	7.0765570	4.5115284	-2.4256792
C	6.1559528	3.5376879	-2.8412938
C	6.0630929	1.1658155	3.3170807
C	4.8637865	1.5444695	4.1696634
C	6.3530554	2.1772116	-2.5251079
C	5.3454842	1.1224498	-2.9474097
N	3.6999410	1.6992215	3.2934074
N	4.3830329	0.8368486	-1.8873705
C	2.8011043	0.6840297	3.0837057

C	3.0638516	1.1976886	-1.9475234
C	2.8687559	1.0046428	0.5875968
C	2.1126814	0.7250075	1.7417281
C	0.7645776	0.3481639	1.6178505
C	0.1407878	0.3076819	0.3503851
C	0.9123711	0.6096765	-0.7950792
C	2.2786831	0.9225228	-0.6870187
H	11.5863069	5.5875087	-2.1210412
H	12.3293304	7.0984839	-1.5834314
H	13.9250871	5.2181543	-1.3669912
H	13.6044207	6.0165021	0.1866862
H	13.7382037	3.6113141	0.5420603
H	12.0902858	4.1552450	0.8899376
H	11.3580042	3.3159207	-1.4161842
H	13.0064359	2.7367628	-1.6983805
H	12.8175876	1.2644227	0.3772478
H	11.1658349	1.8612027	0.6246824
H	10.4704481	0.8708955	-1.6030985
H	12.1248816	0.2888367	-1.8633480
H	11.9789923	-1.2395900	0.1065367
H	10.6151678	-1.5256042	-1.0035065
H	10.2081934	7.2627788	-0.6092293
H	11.1247254	6.5767767	0.7711731
H	6.9018202	3.1737734	3.2628074
H	8.7076798	2.5914424	1.6269508
H	7.2852362	-1.5049050	1.5072152
H	5.4518439	-0.9152060	3.1251205
H	7.6575836	0.7672841	-1.4861188
H	9.2700584	2.4820533	-0.7277456
H	6.9444105	5.5719036	-2.6851315
H	5.2737481	3.8387535	-3.4274059
H	5.0457646	2.4864406	4.7244582
H	4.6114955	0.7526794	4.9007039
H	4.7582954	1.4597048	-3.8230105
H	5.8677129	0.1829900	-3.2252366

H	3.7913658	2.4021633	2.5559200
H	4.7332761	0.3795287	-1.0436557
H	3.9319337	1.2751077	0.6898981
H	0.2110225	0.0692473	2.5268541
H	0.4578587	0.6104605	-1.7970230
C	-14.8081941	-5.4341632	-0.0465401
C	-14.2543427	-5.0157749	1.1735474
C	-13.0318557	-4.3136591	1.1937646
C	-12.4057573	-3.9827824	-0.0248450
C	-12.9730934	-4.3740902	-1.2539796
C	-14.1633720	-5.1301860	-1.2555156
C	-12.3238038	-4.0633728	-2.5800149
O	-12.4142152	-4.8406318	-3.5410824
N	-11.6503942	-2.8741578	-2.6451322
C	-12.4675206	-3.9045893	2.5328080
O	-13.2042341	-3.6624506	3.4978612
N	-11.1011550	-3.8307385	2.5957221
C	-10.8686816	-2.4771579	-3.8137885
C	-10.3805522	-3.3676597	3.7713791
C	-9.3882593	-2.7711081	-3.6409488
C	-8.9387373	-4.1085001	-3.6057154
C	-8.4472799	-1.7370892	-3.4674249
C	-7.5821987	-4.4002931	-3.3989423
C	-7.0889524	-2.0300755	-3.2654967
C	-6.6387117	-3.3655472	-3.2299832
C	-9.4788667	-2.1836933	3.4670502
C	-8.1512817	-2.1409598	3.9416114
C	-9.9597797	-1.0954682	2.7102656
C	-7.3278451	-1.0369377	3.6735075
C	-9.1351941	0.0079282	2.4377903
C	-7.8108621	0.0522944	2.9170256
C	-1.2963041	-0.0569816	0.2204040
C	-2.2375907	0.3508504	1.1907609
C	-3.6027639	0.0403042	1.0528595
C	-4.0301571	-0.7475822	-0.0336945

C	-3.1008339	-1.1958769	-0.9926919
C	-1.7504001	-0.8206148	-0.8775693
C	-3.4998050	-2.0394086	-2.1805689
O	-2.8881567	-1.9711136	-3.2544572
N	-4.5681607	-2.8712943	-1.9758094
C	-4.5527410	0.5353162	2.1167236
O	-4.1914214	0.6646637	3.2947778
N	-5.8151038	0.8369538	1.6850170
C	-5.1654327	-3.6772013	-3.0289739
C	-6.8894697	1.2173896	2.5983347
H	-15.7501735	-6.0031358	-0.0550608
H	-14.7505335	-5.2367740	2.1301595
H	-11.4610712	-3.4159909	-0.0157010
H	-14.5760673	-5.4619867	-2.2198460
H	-11.6375789	-2.2619077	-1.8281426
H	-10.5487093	-4.1388976	1.7933916
H	-11.0337247	-1.3986254	-4.0077320
H	-11.2764217	-3.0484847	-4.6716025
H	-9.7864210	-4.1959509	4.2134498
H	-11.1561104	-3.0918134	4.5161002
H	-9.6691482	-4.9228314	-3.7358438
H	-8.7811236	-0.6871457	-3.4920385
H	-7.2486394	-5.4500829	-3.3691723
H	-6.3654792	-1.2107786	-3.1290060
H	-7.7577730	-2.9844752	4.5314377
H	-10.9922905	-1.1161274	2.3267536
H	-6.2919997	-1.0136615	4.0479885
H	-9.5278275	0.8494047	1.8442338
H	-1.9278822	0.9508571	2.0590417
H	-5.0933027	-1.0171387	-0.1321219
H	-1.0502029	-1.1676655	-1.6514138
H	-4.9663843	-2.9485342	-1.0381468
H	-6.0405299	0.7412215	0.6938341
H	-5.0303723	-4.7586707	-2.8136465
H	-4.5863549	-3.4554370	-3.9500365
H	-7.4596745	2.0578357	2.1545750
H	-6.3990956	1.5831804	3.5223253

[2]Rotaxane **5**

C	-0.0140195	-2.7316162	-2.9544310
C	1.3506448	-3.2574777	-3.4385401
C	1.2727495	-4.6766887	-3.9812061
N	2.0902568	-4.9560366	-5.0286731
C	2.2837639	-6.3156214	-5.5099433
C	3.1524390	-7.0811525	-4.5030322
N	2.4980389	-7.9984551	-3.7546630
C	3.1054594	-8.6221008	-2.6065514
C	2.7630146	-7.9890265	-1.2532905
N	1.9353341	-6.9307222	-1.2654441
C	1.5571485	-6.1969186	-0.0639996
C	0.1324736	-5.6521997	-0.1871405
C	-0.3214626	-4.8024930	1.0051028
C	0.4953283	-3.5221024	1.2435956
C	-0.2181461	-2.5477899	2.1909874
C	0.5561141	-1.2624822	2.5125964
C	1.6971355	-1.4034904	3.5294800
C	2.3235695	-0.0421305	3.8656463
C	3.5277328	-0.0716135	4.8182268
C	4.8096897	-0.6606760	4.2115897
C	6.0338023	-0.4853968	5.1187556
C	7.3455922	-0.9839514	4.4931293
N	7.6700209	-0.3004047	3.2454169
C	7.5206473	-0.8683614	2.0239391
C	7.9224102	-0.0175759	0.8743371
C	8.0268003	-0.5156266	-0.3763135
C	8.4616860	0.3673724	-1.4927181
N	8.9159170	-0.2107795	-2.6400186
C	9.1949484	0.6790949	-3.7852463
C	9.1253799	-1.6530314	-2.7950058
O	0.5520187	-5.5531880	-3.4591176

O	4.3585066	-6.7993996	-4.3523140
O	3.2652915	-8.4606891	-0.2083514
O	8.4109970	1.6177292	-1.3859785
O	7.1011678	-2.0440564	1.8903758
C	0.0765647	-1.5235769	-2.0285594
C	-1.0088181	-1.2299878	-1.1723236
C	-1.0008967	-0.0834119	-0.3656284
C	0.0991937	0.7949973	-0.3953606
C	1.1880062	0.5096094	-1.2330232
C	1.1720248	-0.6401299	-2.0431315
C	-0.9358825	-2.4070874	-4.1299391
C	-2.0559161	-3.2094296	-4.4186429
C	-2.9098247	-2.8920962	-5.4910948
C	-2.6440994	-1.7767699	-6.3017460
C	-1.5231918	-0.9720673	-6.0261550
C	-0.6820409	-1.2831511	-4.9470425
C	10.4978291	1.4520835	-3.6661566
C	10.4878512	2.8263106	-3.3556282
C	11.6896926	3.5426933	-3.2351383
C	12.9200460	2.8913652	-3.4298093
C	12.9415763	1.5207778	-3.7509117
C	11.7372587	0.8066650	-3.8672039
C	7.9160971	-2.4159087	-3.3156232
C	6.8079896	-1.7470703	-3.8664381
C	5.6837969	-2.4627849	-4.3143850
C	5.6596386	-3.8613405	-4.2178145
C	6.7811167	-4.5368877	-3.7092964
C	7.9016592	-3.8258257	-3.2549290
H	-0.4864512	-3.5563882	-2.3817561
H	2.0610406	-3.3232342	-2.5822579
H	1.8059529	-2.5833469	-4.1922148
H	2.6744048	-4.2018139	-5.4140392
H	1.3023090	-6.8093069	-5.6544542
H	2.8194279	-6.2729391	-6.4741103
H	1.5054826	-8.2010230	-3.9687034

H	2.8380396	-9.6939576	-2.5521809
H	4.2012223	-8.5565918	-2.7171643
H	1.5616563	-6.5798109	-2.1636427
H	1.6629956	-6.8821447	0.8021569
H	2.2740937	-5.3610031	0.0783349
H	0.0686766	-5.0556183	-1.1212479
H	-0.5682965	-6.5020185	-0.3333958
H	-1.3834308	-4.5151944	0.8361461
H	-0.3222178	-5.4181013	1.9332737
H	1.5047697	-3.7749540	1.6364242
H	0.6673055	-3.0097819	0.2702694
H	-1.1880008	-2.2707317	1.7220670
H	-0.4801179	-3.0721381	3.1393562
H	0.9579446	-0.8351374	1.5679420
H	-0.1567620	-0.4983417	2.8954608
H	1.3152352	-1.8733968	4.4639856
H	2.4704239	-2.1028382	3.1409906
H	2.6218493	0.4616323	2.9172269
H	1.5314747	0.6056401	4.3020382
H	3.7447461	0.9684557	5.1489232
H	3.2611550	-0.6321594	5.7431636
H	4.6768106	-1.7375144	3.9697840
H	5.0110754	-0.1589721	3.2389158
H	6.1442509	0.5891664	5.3877853
H	5.8844183	-1.0279295	6.0775413
H	8.1899399	-0.8416020	5.1989744
H	7.2750571	-2.0631582	4.2557254
H	8.0108334	0.6627266	3.2931953
H	8.1853490	1.0373055	1.0532697
H	7.7664909	-1.5665204	-0.5689410
H	9.2028267	0.0430627	-4.6911255
H	8.3494324	1.3891458	-3.8802485
H	9.4646751	-2.0769848	-1.8297684
H	9.9868403	-1.7895739	-3.4816750
H	-1.8714733	-1.9151112	-1.1435667

H	-1.8511913	0.1211755	0.3029350
H	0.1113947	1.6895003	0.2456730
H	2.0656870	1.1728651	-1.2566874
H	2.0357094	-0.8440290	-2.6894482
H	-2.2508570	-4.0971897	-3.7977254
H	-3.7879090	-3.5233734	-5.6968127
H	-3.3089932	-1.5323971	-7.1441434
H	-1.3071607	-0.0933063	-6.6532900
H	0.1790004	-0.6342625	-4.7207221
H	9.5264623	3.3346619	-3.1862573
H	11.6623904	4.6101921	-2.9692681
H	13.8628205	3.4497563	-3.3275353
H	13.9013682	1.0056724	-3.9083766
H	11.7620883	-0.2628539	-4.1299062
H	6.8087061	-0.6503424	-3.9174134
H	4.8169947	-1.9304374	-4.7333785
H	4.7845490	-4.4487733	-4.5231949
H	6.7502349	-5.6305448	-3.6414981
H	8.7571405	-4.3682584	-2.8213754
C	-2.3160682	-7.5894385	-6.1066361
C	-1.6736754	-6.2241010	-6.3926024
C	-1.3204972	-6.0586151	-7.8785144
C	-1.1177363	-4.6166558	-8.3658353
C	0.0360067	-3.8438872	-7.7120736
C	0.3993669	-2.5878327	-8.5196595
C	1.5300580	-1.7325662	-7.9094679
O	3.1378313	-2.6684385	-6.3537053
C	2.6959569	-2.5878794	-7.4915701
C	-2.4450198	-7.9389413	-4.6181722
O	-0.1032470	-8.5693935	-4.6179513
C	-1.1846760	-8.5407859	-4.0492584
O	3.1553966	-3.3488816	-8.5313475
O	-1.4041499	-9.0854374	-2.8190673
O	6.8804682	-10.5343441	-6.1474629
O	4.7247512	-12.8173270	-1.7867884
C	5.7381744	-5.6932385	-7.2305469
C	5.0715639	-4.4832576	-7.4702861
C	3.9303246	-4.4909425	-8.2876872
C	3.4888794	-5.6724648	-8.9034917
C	4.1755307	-6.8734026	-8.6586781
C	0.7708780	-10.2680089	-0.0987815
C	-0.1790308	-9.5257446	-0.8159097
C	-0.4061377	-9.8243173	-2.1661511
C	0.2788723	-10.8750616	-2.8012517
C	1.2353763	-11.6002426	-2.0718914
C	5.2960774	-6.9052634	-7.8035036
C	5.9904529	-8.2104392	-7.4713918
C	1.5042562	-11.3000304	-0.7184414
C	2.6889954	-11.9384876	-0.0093136
N	6.0909170	-8.3812745	-6.0247483
N	3.9025601	-11.2001039	-0.3646897
C	6.5180539	-9.5594099	-5.4753215
C	4.7547102	-11.6630167	-1.3343478
C	5.9270487	-10.6726539	-3.2895327
C	6.4614892	-9.5622874	-3.9663415
C	6.8062235	-8.4082403	-3.2387793
C	6.5848431	-8.3293776	-1.8521049
C	6.0212583	-9.4356634	-1.1881443
C	5.6589103	-10.5950994	-1.9084948
C	9.3878008	7.3645070	-0.8702478
C	8.1343226	6.8977296	-1.2989248
C	7.8325097	5.5219596	-1.2295655
C	8.7856130	4.6369391	-0.6963283
C	10.0613565	5.0868821	-0.3131529
C	10.3543812	6.4641634	-0.3922276
C	11.1609180	4.1403383	0.1017125
O	12.2322358	4.5577384	0.5671390
N	10.9172348	2.8111845	-0.1250139
C	6.5063257	5.0397076	-1.7650341
O	5.5603928	5.8182536	-1.9604310

N	6.4465415	3.7040636	-2.0596001
C	11.9887543	1.8286733	-0.0763124
C	5.2366669	3.0611023	-2.5441492
C	11.5433702	0.4966417	0.4839805
C	11.5659197	-0.6649575	-0.3145133
C	11.1389353	0.3830849	1.8290030
C	11.2422371	-1.9167643	0.2317504
C	10.8173659	-0.8693190	2.3761450
C	10.8825091	-2.0356131	1.5901491
C	4.9213891	1.8038280	-1.7590182
C	4.7878327	0.5619323	-2.4094690
C	4.7620882	1.8498104	-0.3589952
C	4.4661352	-0.5971158	-1.6907455
C	4.4127721	0.6938654	0.3565307
C	4.2340644	-0.5360726	-0.3043163
C	6.7673150	-7.0129692	-1.1655827
C	5.6758490	-6.1153030	-1.1350856
C	5.8127270	-4.8424313	-0.5546525
C	7.0575850	-4.4668580	-0.0286594
C	8.1465044	-5.3509103	-0.0297681
C	8.0038220	-6.6232975	-0.6169439
C	9.4681340	-4.9443566	0.5656284
O	10.5396308	-5.4540265	0.2021310
N	9.3839654	-3.9946445	1.5488613
C	4.6452272	-3.8888463	-0.5557813
O	3.6721343	-4.0520991	-1.3121993
N	4.7297107	-2.8672853	0.3484061
C	10.5598732	-3.4018966	2.1672585
C	3.7640806	-1.7821662	0.4179346
H	-1.7312104	-8.3903776	-6.6078624
H	-3.3266126	-7.6137402	-6.5668008
H	-2.3813695	-5.4256471	-6.0906362
H	-0.7786451	-6.0788352	-5.7504252
H	-0.4084222	-6.6584835	-8.1039478
H	-2.1332918	-6.5140305	-8.4880905

H	-0.9478384	-4.6441939	-9.4657201
H	-2.0581155	-4.0401302	-8.2182531
H	-0.2228811	-3.5691019	-6.6670041
H	0.9246773	-4.5122851	-7.6391127
H	0.6965490	-2.8928918	-9.5456127
H	-0.4985696	-1.9430033	-8.6266989
H	1.1900790	-1.2033325	-7.0003230
H	1.8765669	-0.9756983	-8.6443567
H	-3.2666156	-8.6584333	-4.4227044
H	-2.6868556	-7.0424613	-4.0038188
H	6.6070596	-5.6903779	-6.5547403
H	5.4190647	-3.5545969	-7.0029273
H	2.5937422	-5.6442623	-9.5409716
H	3.8110249	-7.8053029	-9.1175018
H	0.9756898	-10.0088547	0.9504589
H	-0.7380103	-8.7064552	-0.3438970
H	0.0821920	-11.1094894	-3.8557210
H	1.8091689	-12.3958409	-2.5716996
H	7.0117867	-8.2330111	-7.9149560
H	5.4478377	-9.0699842	-7.9218798
H	2.5457022	-11.9134551	1.0903936
H	2.8327803	-12.9908097	-0.3216875
H	5.5612630	-7.7224998	-5.4236966
H	3.8169384	-10.1655045	-0.2450889
H	5.6199577	-11.5686255	-3.8490149
H	7.2072706	-7.5344145	-3.7706604
H	5.7948011	-9.3523912	-0.1166241
H	9.6209939	8.4386532	-0.9276036
H	7.3741125	7.5829026	-1.7026631
H	8.5247398	3.5787314	-0.5756042
H	11.3536227	6.8051171	-0.0832857
H	10.0790035	2.5242177	-0.6481424
H	7.2446013	3.0775602	-1.8799158
H	12.7939078	2.2664871	0.5474787
H	12.4096312	1.6864338	-1.0951537

H	5.3308221	2.8146767	-3.6249355
H	4.4197325	3.8074178	-2.4553240
H	11.8602523	-0.5823553	-1.3726110
H	11.0984609	1.2868005	2.4576876
H	11.2946998	-2.8263538	-0.3879943
H	10.5220518	-0.9454073	3.4344043
H	4.9172273	0.5093362	-3.5019221
H	4.8983856	2.8054501	0.1711021
H	4.3635336	-1.5617245	-2.2103155
H	4.2523435	0.7535622	1.4439261
H	4.7227665	-6.3716405	-1.6153919
H	7.1956303	-3.4534291	0.3656552
H	8.8701068	-7.3008610	-0.6422273
H	8.4825318	-3.5462658	1.7592531
H	5.5826251	-2.7334932	0.9114257
H	11.4012845	-4.1011291	1.9878400
H	10.4007515	-3.3298351	3.2624829
H	3.5545206	-1.5537660	1.4807453
H	2.8200573	-2.1392373	-0.0413784

[4]Pseudorotaxane **5·5**

C	2.4119834	-9.3038713	-6.5000179
C	1.6815605	-10.4078213	-7.2820378
C	0.2592349	-10.7007389	-6.7756420
C	-0.7154155	-9.5216852	-6.9062362
C	-2.0202403	-9.7203176	-6.1259242
C	-2.8796270	-8.4510396	-6.0701089
N	-3.6150358	-8.3431572	-4.8142472
C	-4.1042048	-7.1526646	-4.4015018
C	-4.5989034	-7.1374798	-2.9993948
C	-4.9735432	-5.9895903	-2.3996278
C	-5.1938652	-5.9668567	-0.9300081
N	-4.7430691	-4.8598445	-0.2631145
C	-4.9015612	-4.8052986	1.1935982
C	-3.9798514	-3.7861628	-0.9002846
O	-5.6798212	-6.9363896	-0.3138548
O	-4.0644971	-6.1255476	-5.1265147
C	-3.7312178	-5.4180295	1.9503943
C	-3.2701217	-4.8434641	3.1514576
C	-2.2065772	-5.4256654	3.8637345
C	-1.5928992	-6.5969855	3.3861957
C	-2.0584445	-7.1886289	2.1986992
C	-3.1149978	-6.6005452	1.4879096
C	-4.8061270	-2.5802648	-1.3156222
C	-4.1864202	-1.3187115	-1.4074386
C	-4.8900480	-0.2100646	-1.9047606
C	-6.2270181	-0.3546672	-2.3143288
C	-6.8578981	-1.6053337	-2.2098293
C	-6.1524594	-2.7118484	-1.7085754
C	6.9373398	-1.6208507	-0.3800387
C	8.0348983	-0.5269785	-0.5775981
C	7.5766287	0.5577787	-1.5452004
N	7.5721786	0.1523012	-2.8628817
C	6.8391848	0.8423516	-3.8991034
C	5.5745546	0.0610314	-4.2796537
N	5.2825615	0.0477850	-5.5981681
C	4.0387342	-0.4544003	-6.1368892
C	3.9625905	-1.9791805	-6.2369170
N	3.1207866	-2.4516830	-7.1807485
C	2.8051424	-3.8759441	-7.2375789
C	3.8692951	-4.6717102	-8.0095847
C	3.8365558	-6.1895665	-7.7878061
C	4.0670809	-6.6140542	-6.3230852
C	4.6439437	-8.0329821	-6.1731504
C	3.8789567	-9.1429788	-6.9146302
O	7.2259889	1.6856837	-1.1913096
O	4.8775141	-0.4711222	-3.3974438
O	4.5671248	-2.7190107	-5.4387624
C	7.6126217	-2.9435240	-0.0144171
C	7.9543041	-3.8320119	-1.0547840

C	8.6975627	-4.9963751	-0.8064960
C	9.1193303	-5.2856898	0.5021557
C	8.7636700	-4.4223808	1.5537565
C	8.0089012	-3.2640482	1.2992499
C	5.8004376	-1.2291765	0.5556945
C	5.9876535	-0.5834892	1.7956131
C	4.8961865	-0.3553689	2.6548621
C	3.5995319	-0.7546245	2.2842758
C	3.3950626	-1.3687871	1.0375346
C	4.4900022	-1.5988706	0.1936611
H	2.3632562	-9.5547211	-5.4191880
H	1.8809715	-8.3355279	-6.6291186
H	1.6542329	-10.1408749	-8.3640272
H	2.2802705	-11.3413521	-7.2069834
H	-0.1566482	-11.5839880	-7.3087690
H	0.3383351	-11.0021810	-5.7070990
H	-0.2310508	-8.6014138	-6.5137413
H	-0.9278809	-9.3158283	-7.9796095
H	-2.6009291	-10.5773387	-6.5293512
H	-1.7515913	-9.9984404	-5.0878231
H	-2.2371412	-7.5461586	-6.1485823
H	-3.5825751	-8.3929326	-6.9307074
H	-3.6268885	-9.1411120	-4.1494439
H	-4.5427812	-8.0714577	-2.4202347
H	-5.0371025	-5.0606270	-2.9824206
H	-5.0469318	-3.7457102	1.4847970
H	-5.8312882	-5.3547054	1.4455450
H	-3.4506518	-4.1964219	-1.7862129
H	-3.1696636	-3.4833377	-0.2055539
H	-3.7359100	-3.9183631	3.5258973
H	-1.8461148	-4.9518441	4.7895216
H	-0.7518165	-7.0430647	3.9388915
H	-1.6258061	-8.1240783	1.8186045
H	-3.4849896	-7.0879749	0.5762172
H	-3.1327213	-1.2228910	-1.1098689

H	-4.3870648	0.7681292	-1.9869427
H	-6.7786297	0.5061195	-2.7225249
H	-7.8987735	-1.7246929	-2.5444329
H	-6.6540845	-3.6882614	-1.6359456
H	6.4675383	-1.7791164	-1.3748518
H	8.9464840	-1.0132619	-0.9819025
H	8.3139090	-0.0510217	0.3806482
H	7.8128208	-0.8225747	-3.0559541
H	6.5115198	1.8190474	-3.4842100
H	7.4712126	1.0460282	-4.7870333
H	5.9329706	0.5208383	-6.2499106
H	3.1982522	-0.1565215	-5.4746881
H	3.8489973	0.0178803	-7.1214619
H	2.6052426	-1.7853536	-7.7802854
H	2.7440059	-4.2430824	-6.1917335
H	1.8041719	-3.9938179	-7.6990657
H	3.7910838	-4.4322733	-9.0916094
H	4.8563874	-4.2942575	-7.6742092
H	2.8811711	-6.6167876	-8.1656699
H	4.6331655	-6.6375259	-8.4221591
H	4.7606874	-5.8921225	-5.8390801
H	3.1228246	-6.5301296	-5.7416096
H	5.6978818	-8.0255656	-6.5287566
H	4.6997105	-8.2822539	-5.0910636
H	3.9323112	-8.9622303	-8.0120232
H	4.4038053	-10.1097081	-6.7487315
H	7.6234069	-3.6029065	-2.0799115
H	8.9171542	-5.6797610	-1.6395314
H	9.7016330	-6.1970098	0.7072100
H	9.0691286	-4.6561132	2.5855270
H	7.7287208	-2.6014826	2.1317443
H	6.9906419	-0.2504939	2.1040616
H	5.0618153	0.1474306	3.6202784
H	2.7483911	-0.5671104	2.9570752
H	2.3905599	-1.6731379	0.7076835

H	4.3380302	-2.0675755	-0.7839309
C	-4.9435920	-11.7668715	1.7691800
C	-6.2190471	-11.1839585	1.6810277
C	-6.5572885	-10.3914450	0.5657382
C	-5.6268093	-10.2394746	-0.4765178
C	-4.3556530	-10.8400699	-0.4084704
C	-4.0094416	-11.5955496	0.7329301
C	-3.4554454	-10.6660503	-1.5966170
O	-3.9251324	-10.3599957	-2.7146631
N	-2.1199645	-10.8501979	-1.3926800
C	-7.9317685	-9.7679710	0.4498227
O	-8.9613529	-10.3698628	0.7917068
N	-7.9030987	-8.5203193	-0.0881490
C	-1.1991581	-10.5282939	-2.4627411
C	-9.0632313	-7.7815870	-0.5564239
C	-0.9306619	-9.0402228	-2.6105686
C	-1.5537807	-8.0815247	-1.7889693
C	-0.0451138	-8.6030563	-3.6117275
C	-1.3260487	-6.7146109	-1.9864594
C	0.1723713	-7.2360054	-3.8197350
C	-0.4722624	-6.2752622	-3.0168078
C	-8.7152600	-7.1156563	-1.8776788
C	-8.7767583	-5.7173146	-2.0425020
C	-8.2480612	-7.8982808	-2.9537354
C	-8.4024887	-5.1203122	-3.2572912
C	-7.8647552	-7.3013609	-4.1642000
C	-7.9423353	-5.9055226	-4.3336630
C	-3.8217069	-0.2349729	-5.2855836
C	-5.0267548	-0.9454107	-5.4649646
C	-5.1149356	-2.3093110	-5.1304656
C	-4.0031490	-2.9469429	-4.5553706
C	-2.8291625	-2.2280902	-4.2887842
C	-2.7283894	-0.8802182	-4.6751030
C	-1.7260500	-2.8596059	-3.4982954
O	-1.1664085	-2.2543530	-2.5684225
N	-1.3851202	-4.1335973	-3.8522802
C	-6.4206069	-3.0328562	-5.3480020
O	-7.4929956	-2.4121501	-5.4397374
N	-6.3309581	-4.3920931	-5.4461774
C	-0.2436833	-4.8044418	-3.2426348
C	-7.5064914	-5.2349480	-5.6242929
C	-2.5614412	2.9768175	-6.9173481
C	-2.7484591	1.6112525	-6.6239207
C	-3.6978301	1.1998872	-5.6686371
C	-4.4675847	2.1774462	-5.0093165
C	-4.3224565	3.5396249	-5.3317081
C	-3.3979901	3.9330011	-6.3128034
C	-1.3544207	3.3535077	-7.7436358
C	-5.0766104	4.6006101	-4.5754083
N	-0.7490689	4.5070054	-7.3270138
N	-5.0788135	4.3918748	-3.2260686
C	0.5977201	4.8737573	-7.7680101
C	1.6247864	4.5111391	-6.7052298
C	-5.7674908	5.2737405	-2.2875774
C	-4.8041776	5.8707780	-1.2795648
C	1.6172501	5.2027945	-5.4743143
C	2.4332394	4.7962307	-4.4136314
C	3.2724661	3.6853117	-4.5876989
C	3.3629340	3.0308229	-5.8204277
C	2.5299033	3.4455973	-6.8748325
C	-4.1403625	5.0302871	-0.3601089
C	-3.1483262	5.5354109	0.4905679
C	-2.8366156	6.9016804	0.4319514
C	-3.5174462	7.7720373	-0.4314577
C	-4.4977587	7.2452583	-1.2884633
O	-0.9127934	2.6292804	-8.6544555
O	-5.6169490	5.5684164	-5.1396449
O	4.0239900	3.1838319	-3.5069222
O	-1.8031626	7.3783268	1.2479876
C	3.3043252	2.6821701	-2.4517943

O	2.0798005	2.6915797	-2.4431899	H	0.6616627	-4.6686560	-3.8767558
C	4.1678690	2.0971277	-1.3758036	H	-7.2845574	-5.9996813	-6.3958174
C	-0.5996194	7.6303729	0.6292060	H	-8.3171131	-4.5821576	-6.0067862
O	-0.4466630	7.5452915	-0.5769365	H	-2.1217984	0.8706429	-7.1372097
C	0.4691018	7.9333422	1.6477859	H	-5.1739709	1.8708479	-4.2244682
C	0.7774839	6.6917037	2.5168117	H	-3.3007940	5.0011072	-6.5495153
C	1.1357729	5.4461744	1.6944937	H	-1.0418220	4.8705240	-6.3959891
C	1.6671720	4.2832076	2.5446596	H	-4.3282954	3.7798633	-2.8456599
C	1.9724854	2.9999620	1.7523392	H	0.8006621	4.3418187	-8.7159633
C	2.9511881	3.2078761	0.5881154	H	0.6254068	5.9657459	-7.9618589
C	3.4103561	1.8966791	-0.0626171	H	-6.5575951	4.6973473	-1.7569735
H	-4.6774565	-12.3741074	2.6475907	H	-6.2670389	6.0642160	-2.8807397
H	-6.9645172	-11.3325408	2.4768658	H	0.9324067	6.0473775	-5.3261831
H	-5.8857603	-9.6630630	-1.3753163	H	2.3987023	5.3158180	-3.4460418
H	-3.0263657	-12.0847570	0.8002156	H	4.0692434	2.2001723	-5.9375353
H	-1.7220031	-10.7223860	-0.4416886	H	2.5657472	2.9099066	-7.8334112
H	-6.9941145	-8.0402080	-0.2260818	H	-4.3604994	3.9537108	-0.3395755
H	-1.6167812	-10.9216353	-3.4117038	H	-2.5937506	4.8780135	1.1758902
H	-0.2471266	-11.0670762	-2.2955423	H	-3.2539796	8.8387422	-0.4514905
H	-9.3847166	-7.0158667	0.1838024	H	-5.0118410	7.9134858	-1.9964105
H	-9.9029381	-8.5012334	-0.6615859	H	5.0846425	2.7073578	-1.2597753
H	-2.2174785	-8.4032044	-0.9747326	H	4.5352054	1.1231738	-1.7754876
H	0.4940160	-9.3443285	-4.2188837	H	0.1262518	8.7711173	2.2891388
H	-1.8158347	-5.9801275	-1.3281389	H	1.3676205	8.2593554	1.0894764
H	0.8768397	-6.9114164	-4.6024929	H	1.6198921	6.9500801	3.1931250
H	-9.1225765	-5.0868046	-1.2074496	H	-0.0950552	6.4766450	3.1695043
H	-8.1692606	-8.9903048	-2.8322812	H	0.2410289	5.1026345	1.1243004
H	-8.4613804	-4.0280230	-3.3843418	H	1.8867766	5.7280726	0.9253424
H	-7.4908707	-7.9300312	-4.9884832	H	2.5909228	4.6233422	3.0675123
H	-5.9143830	-0.4482368	-5.8835282	H	0.9307376	4.0542570	3.3463755
H	-4.0529224	-4.0076738	-4.2751795	H	2.3910371	2.2372786	2.4466381
H	-1.8013641	-0.3226068	-4.4829596	H	1.0271280	2.5655337	1.3563745
H	-1.8715450	-4.5903939	-4.6303613	H	2.4633096	3.8432337	-0.1817512
H	-5.4272760	-4.8905050	-5.3488989	H	3.8441077	3.7766116	0.9367067
H	-0.0411350	-4.2686090	-2.2938033	H	2.5293333	1.2438883	-0.2441837

H	4.0633016	1.3346482	0.6325447
C	3.1797826	-4.1957899	-2.1415844
C	2.6290240	-5.4382405	-1.7877935
C	3.2914024	-6.6468902	-2.0879971
C	4.5539029	-6.5755311	-2.7181709
C	5.1271239	-5.3371989	-3.0600050
C	4.4396997	-4.1480533	-2.7627063
C	2.3467711	-2.9663563	-1.9034012
C	6.4897367	-5.3042279	-3.7005910
N	2.6758254	-1.8764635	-2.6390750
N	6.7659104	-4.1698009	-4.4117166
C	1.8406254	-0.7000862	-2.7102481
C	1.2065354	-0.5828880	-4.0874849
C	8.0806893	-3.9504377	-5.0070190
C	8.0221610	-2.7975049	-5.9855569
C	0.9832291	0.6644094	-4.7065326
C	0.4702395	0.7311449	-6.0103023
C	0.1500840	-0.4577673	-6.6770506
C	0.3165960	-1.7062111	-6.0704768
C	0.8691582	-1.7572857	-4.7881229
C	7.2084041	-2.9059426	-7.1317823
C	7.0927749	-1.8514164	-8.0437996
C	7.8284812	-0.6786586	-7.8176117
C	8.6565547	-0.5410947	-6.6958646
C	8.7377346	-1.6024375	-5.7770123
O	1.3844375	-2.9804305	-1.1148021
O	7.3141862	-6.2229258	-3.5583808
O	-0.4145370	-0.3823346	-7.9531161
O	7.7614008	0.3317792	-8.7879978
C	0.4127703	-0.3702771	-9.0379857
O	1.6150664	-0.5930811	-8.9588555
C	-0.3865167	-0.1049908	-10.2869568
C	7.0996417	1.4908199	-8.4984138
O	6.5861567	1.7121747	-7.4095745
C	7.0038160	2.3790490	-9.7057241

C	5.8091743	1.9408025	-10.5909416
C	4.4288756	2.1987459	-9.9715730
C	3.2827072	1.7028424	-10.8661719
C	1.9043705	2.2699633	-10.5044923
C	0.7864390	1.8631099	-11.4781766
C	0.4135150	0.3678429	-11.5064232
C	10.1286602	-10.3879761	1.5102123
C	10.2646640	-11.6134557	0.8355620
C	9.6741957	-12.7836712	1.3626089
C	8.9899834	-12.7125322	2.5908730
C	8.7890288	-11.4770202	3.2331088
C	9.3778584	-10.3142942	2.6950686
C	7.8670446	-11.3209918	4.4187657
O	7.9274780	-10.3309050	5.1629902
N	6.9268014	-12.3035054	4.5587194
C	9.6638980	-14.0128046	0.4856105
O	10.4085655	-14.1019780	-0.5019291
N	8.7059734	-14.9511686	0.7791737
C	5.7868430	-12.1103306	5.4559338
C	8.3083432	-15.9450886	-0.2161563
C	4.7585610	-11.1914444	4.8153836
C	4.7955719	-9.7992991	5.0365902
C	3.8215486	-11.7015626	3.8956901
C	3.9186879	-8.9469816	4.3489596
C	2.9576945	-10.8508743	3.1908744
C	3.0019607	-9.4597725	3.4090220
C	7.0810392	-15.4803676	-0.9834121
C	5.8799196	-16.2097148	-0.9587149
C	7.1195252	-14.2622071	-1.7013311
C	4.7383145	-15.7258265	-1.6279637
C	5.9786278	-13.7748331	-2.3486507
C	4.7643732	-14.4948187	-2.3108028
C	2.6874259	-7.9530828	-1.7197751
C	2.9402310	-9.1103997	-2.4869172
C	2.4465295	-10.3601663	-2.0880333

C	1.6964861	-10.4701405	-0.9076076	H	4.3194535	3.2907101	-9.7836740
C	1.3621033	-9.3202222	-0.1744794	H	3.5070631	1.9747046	-11.9231844
C	1.8508361	-8.0677218	-0.5856586	H	3.2529084	0.5924224	-10.8312531
C	0.4443237	-9.4934810	1.0038280	H	1.6197555	1.9839573	-9.4707480
O	-0.6629440	-10.0595849	0.8809742	H	1.9689270	3.3808767	-10.4955544
N	0.8475323	-9.0310766	2.2176913	H	-0.1242098	2.4467520	-11.2267465
C	2.6405655	-11.5839716	-2.9481493	H	1.0868881	2.1693178	-12.5053377
O	2.2795706	-11.6125044	-4.1345698	H	1.3181878	-0.2657511	-11.6266261
N	3.1786136	-12.6415608	-2.2753043	H	-0.2117345	0.1809081	-12.4037235
C	2.1497312	-8.5038678	2.5961415	H	10.5863228	-9.4789813	1.0913602
C	3.5135799	-13.9056499	-2.9307773	H	10.8026016	-11.6851726	-0.1211925
H	1.6479601	-5.4445438	-1.2962881	H	8.5704595	-13.6240632	3.0382992
H	5.1352226	-7.4844528	-2.9252609	H	9.2151884	-9.3579590	3.2137004
H	4.9009275	-3.1808004	-3.0049587	H	6.8080546	-13.0268487	3.8287933
H	3.5240496	-1.8337225	-3.2238795	H	8.0140768	-14.7664371	1.5189027
H	5.9927504	-3.5450048	-4.7089484	H	6.1577033	-11.6808221	6.4061861
H	1.0691229	-0.8040089	-1.9222648	H	5.3512808	-13.1068415	5.6692051
H	2.4346567	0.2019445	-2.4833857	H	8.1056848	-16.9109549	0.2835853
H	8.4137821	-4.8842085	-5.5126604	H	9.1697449	-16.0781788	-0.8998403
H	8.8328001	-3.7522819	-4.2111449	H	5.5340817	-9.3876578	5.7396148
H	1.2419971	1.5978252	-4.1902946	H	3.7653488	-12.7858573	3.7208685
H	0.3330970	1.6935532	-6.5186859	H	3.9524440	-7.8619911	4.5386103
H	0.0326550	-2.6200198	-6.6101866	H	2.2428740	-11.2781875	2.4709290
H	1.0676340	-2.7300739	-4.3246861	H	5.8251315	-17.1560183	-0.3974033
H	6.6482262	-3.8355534	-7.3068151	H	8.0568977	-13.6862193	-1.7373428
H	6.4529300	-1.9310341	-8.9344447	H	3.8079037	-16.3149623	-1.6109617
H	9.2335748	0.3827643	-6.5504012	H	6.0191211	-12.8082844	-2.8739847
H	9.3877111	-1.4981576	-4.8938617	H	3.5165410	-9.0387787	-3.4198876
H	-1.1734898	0.6249760	-10.0093935	H	1.3433615	-11.4513196	-0.5669666
H	-0.9083012	-1.0642919	-10.5042911	H	1.5817376	-7.1694316	-0.0118727
H	6.8639313	3.4198700	-9.3552973	H	0.1944986	-9.2482715	2.9786811
H	7.9470066	2.3096798	-10.2814461	H	3.7093880	-12.3674671	-1.4242939
H	5.8847719	2.4906313	-11.5526584	H	2.0148150	-7.5458238	3.1401699
H	5.9225845	0.8636473	-10.8439559	H	2.7000606	-8.2494866	1.6716582
H	4.3595772	1.7185070	-8.9695900	H	3.6476814	-13.7038467	-4.0162822

H	2.6683669	-14.6233380	-2.8452761
C	4.5449384	-4.8762042	1.8897486
C	4.6252246	-6.3157106	1.3788781
C	5.8770087	-6.6122338	0.5563768
C	5.9511706	-8.0622047	0.0748062
C	7.1861507	-8.3487240	-0.7858400
C	7.1667226	-9.7424804	-1.4272655
N	7.0918817	-10.8298405	-0.4581393
C	5.9263403	-11.4490126	-0.1294318
C	6.0718462	-12.5571959	0.8452843
C	5.0255460	-13.3197875	1.2204585
C	5.2457890	-14.4603223	2.1431331
N	4.2449018	-15.3811904	2.2621040
C	4.4529539	-16.5296508	3.1669731
C	3.0881596	-15.4095495	1.3564024
O	6.3161376	-14.5706855	2.7985629
O	4.8299752	-11.1550696	-0.6427034
C	5.2111747	-17.6585733	2.4887081
C	4.5274240	-18.6259853	1.7213724
C	5.2351584	-19.6259728	1.0323796
C	6.6372882	-19.6831154	1.1207124
C	7.3248601	-18.7371884	1.9021200
C	6.6180747	-17.7247657	2.5728593
C	1.9419039	-14.4841852	1.7212769
C	1.1714985	-13.9093928	0.6886776
C	0.0728833	-13.0885100	0.9911241
C	-0.2526963	-12.8074758	2.3291953
C	0.5090772	-13.3790131	3.3630771
C	1.5943918	-14.2194503	3.0602541
C	-2.1607420	7.7447716	-5.4281801
C	-2.6245500	7.0787852	-4.1210046
C	-1.8201534	5.8339363	-3.7909432
N	-1.6052661	5.5923975	-2.4795450
C	-0.8383862	4.4483290	-2.0631362
C	-1.5837944	3.1122581	-2.1938671
N	-0.7453807	2.0485281	-2.2175619
C	-1.2073509	0.7311166	-1.8648662
C	-1.0067775	0.4952587	-0.3555326
N	-0.8590658	-0.8011732	0.0346082
C	-0.7458820	-1.1374947	1.4510605
C	-1.3203407	-2.5166358	1.7956681
C	-0.4187451	-3.6997293	1.4004171
C	0.8058194	-3.8089723	2.3251485
C	1.9820469	-4.6109536	1.7588626
C	3.2454638	-4.5425297	2.6355918
O	-1.3835915	5.0596284	-4.6629610
O	-2.8217750	3.0075388	-2.2178457
O	-1.0179377	1.4519278	0.4331346
C	-3.1017114	8.9001967	-5.7748712
C	-2.7058479	10.2487551	-5.6757471
C	-3.6115249	11.2859666	-5.9649691
C	-4.9265712	10.9870038	-6.3579485
C	-5.3316973	9.6423259	-6.4547059
C	-4.4284533	8.6078217	-6.1652850
C	-0.6862845	8.1301354	-5.4173919
C	0.0549358	8.3019645	-4.2283997
C	1.4221426	8.6328797	-4.2751335
C	2.0702334	8.8035575	-5.5089149
C	1.3386133	8.6491801	-6.7010544
C	-0.0243417	8.3188155	-6.6510745
H	4.6561609	-4.1758943	1.0319530
H	5.4204949	-4.6732021	2.5455285
H	4.5807397	-7.0169105	2.2429428
H	3.7346810	-6.5339575	0.7515442
H	5.9106452	-5.9266808	-0.3190555
H	6.7821479	-6.3664906	1.1508001
H	5.9331564	-8.7466337	0.9529318
H	5.0366806	-8.3163266	-0.5031818
H	7.2553995	-7.6056573	-1.6097677
H	8.1099447	-8.2276428	-0.1770600

H	6.2737599	-9.8437489	-2.0778077
H	8.0682731	-9.8992267	-2.0543058
H	7.9407581	-11.0717685	0.0634763
H	7.0724437	-12.7737293	1.2379477
H	4.0231860	-13.0823278	0.8364003
H	5.0086781	-16.1633493	4.0497833
H	3.4547238	-16.8721330	3.5013372
H	2.7228314	-16.4554809	1.3324808
H	3.4278537	-15.2033244	0.3238033
H	3.4271734	-18.6067617	1.6674183
H	4.6879501	-20.3700488	0.4336826
H	7.1933718	-20.4693347	0.5878029
H	8.4213069	-18.7864656	1.9881605
H	7.1544704	-16.9655308	3.1601789
H	1.4431521	-14.0953830	-0.3632829
H	-0.5204626	-12.6445461	0.1789871
H	-1.0817537	-12.1210569	2.5544990
H	0.2660807	-13.1605064	4.4138647
H	2.1869220	-14.6600232	3.8769067
H	-2.2761948	6.9818020	-6.2289644
H	-3.6846931	6.7711309	-4.2303852
H	-2.6053229	7.7984707	-3.2787721
H	-1.8258065	6.3103927	-1.7828743
H	0.1132592	4.3805424	-2.6321014
H	-0.5842122	4.5660724	-0.9901682
H	0.2623803	2.2355333	-2.1237779
H	-2.2899164	0.6783541	-2.0870596
H	-0.7371282	-0.0524743	-2.4804793
H	-0.7341131	-1.5309674	-0.6832784
H	0.3201310	-1.0735354	1.7698426
H	-1.2730578	-0.3369477	2.0060032
H	-1.5000618	-2.5501122	2.8916603
H	-2.3261904	-2.6068745	1.3310492
H	-1.0011431	-4.6433909	1.4327623
H	-0.0749265	-3.5771243	0.3542469
H	1.1840572	-2.7859734	2.5472457
H	0.4881419	-4.2244202	3.3087568
H	1.6678999	-5.6665160	1.5932704
H	2.2214011	-4.2067492	0.7535868
H	3.3314694	-3.5096511	3.0375111
H	3.1344063	-5.2074009	3.5238977
H	-1.6756240	10.4908455	-5.3733712
H	-3.2835425	12.3341791	-5.8835799
H	-5.6350713	11.7977122	-6.5888897
H	-6.3611856	9.3964250	-6.7592055
H	-4.7633961	7.5587666	-6.2198622
H	-0.4119161	8.1543736	-3.2437841
H	1.9828007	8.7441068	-3.3343797
H	3.1425447	9.0491907	-5.5439699
H	1.8356826	8.7758518	-7.6751952
H	-0.5938052	8.1948181	-7.5862644

15. References.

[1] A. Martinez-Cuezva, A. Saura-Sanmartin, T. Nicolas-Garcia, C. Navarro, R.A. Orenes, M. Alajarin, J. Berna, Chem. Sci. **2017**, *8*, 3775-3780.
[2] D. González Cabrera, B. D. Koivisto, D. A. Leigh, *Chem. Commun*. **2007**, 4218–4220.
[3] M. Chen, S. Han, L. Jiang, S. Zhou, F. Jiang, Z. Xu, J. Liang, S. Zhang, *Chem. Commun*. **2010**, *46*, 3932-3934.
[4] E. O. Stejskal, J. E. Tanner, *J. Chem. Phys.* **1965**, *42*, 288-292.
[5] M. Holz, H. Weingärtner, *J. Magn. Reson*. **1991**, *92*, 115–125.
[6] a) A. Saura-Sanmartin, A. Martinez-Cuezva, A. Pastor, D. Bautista, J. Berna, *Org. Biomol. Chem.* **2018**, *16*, 6980-6987; b) A. Saura-Sanmartin, A. Pastor, A. Martinez-Cuezva, J. Berna, *Chem. Commun.* **2022**, *58*, 290-293.
[7] (a) T. A. Halgren, *J. Comput. Chem.* **1996**, *17*, 490-519; (b) T. A. Halgren, *J. Comput. Chem.* **1996**, *17*, 520-5552.
[8] A. Schäfer, H. Horn, R. Ahlrichs, *J. Chem. Phys.* **1992**, *97*, 2571–2577.
[9] A. Klamt, G. Schüürmann. *J. Chem. Soc. Perkin Trans.2* **1993**, *5*, 799–805.
[10] S. Grimme, J. Antony, S. Ehrlich, H. Krieg, *J. Chem. Phys.* **2010**, *132*, 154104–154119.
[11] G. Mahmoudi, S. K. Seth, F. I. Zubkov, E. López-Torres, A. Bacchi, V. Stilinović, A. Frontera, *Crystals* **2019**, *9*, 323.
[12] TURBOMOLE V7.2 2017, a development of University of Karlsruhe and Forschungszentrum Karlsruhe GmbH, 1989–2007, TURBOMOLE GmbH, since 2007. 2007; available from http://www.turbomole.com.
